# Commonality of 25 component themes of integrated care for children: rapid review of 170 models

**DOI:** 10.1186/s12913-025-13345-w

**Published:** 2025-10-08

**Authors:** Evgenia Stepanova, Frances Hillier-Brown, Emily Owen-Boukra, Steven Hope, Steph Scott, Dougal Hargreaves, Dasha Nicholls, Russell M. Viner, Carolyn Summerbell

**Affiliations:** 1 Department of Sport and Exercise Sciences, Durham University, Durham, UK; 2 Fuse - Centre for Translational Research in Public Health, Newcastle Upon Tyne, UK; 3 Population Health Sciences Institute, Newcastle University, Newcastle Upon Tyne, UK; 4 Nuffield Department of Primary Care Health Sciences, University of Oxford, Oxford, UK; 5 Mohn Centre for Children’s Health & Wellbeing, School of Public Health, Imperial College London, London, UK; 6 Division of Psychiatry, Department of Brain Sciences, Imperial College London, London, UK; 7 Population, Policy and Practice Research and Teaching Department, UCL Great Ormond Street Institute of Child Health, UCL, London, UK

**Keywords:** Integrated care, Integration, Components, Children and young people, Rapid review, Framework analysis, Guidance, Public health

## Abstract

**Introduction:**

The components of integrated care for children, young people and families (CYPF) listed across existing authoritative guidance is generally consistent; the guidance suggests a list of components that should ideally be considered for implementation. Local system managers report specific challenges around integrating system-wide funding, trusted workforce relationships, and CYPF engagement. We aimed to systematically generate a list of components of integrated care from existing systems and models for CYPF, assess their commonality, intended target(s) of impact, and compare these findings with guidance and local system managers’ concerns.

**Methods:**

PubMed, CINHAL and Cochrane CENTRAL were systematically searched (01/01/2016 to 31/12/2023) for studies of any design, conducted in high-income countries, describing components of an integrated care system or model for CYPF. Following data extraction, individual components within studies were coded a) using the framework method to generate Component Themes b) for their intended target(s) of impact; system (S), users (U) and/or workforce (W). Simple analytic methods were used to rank and map the commonality of Component Themes and their intended target(s) of impact. Subgroup analysis was conducted for four public health priorities: mental health, learning disabilities and autism, obesity, and early years.

**Results:**

From 170 studies, 1057 components nested in one or more 25 Component Themes of integration were identified. None of the studies identified all Component Themes (median 5, range 1–16). Most commonly identified were ‘shared professional responsibility and practices’ (in 58% of studies; intended targets of impact S and W), ‘stronger connections and partnerships’ (52%;SUW), ‘empowerment of service users’ (36%;U), ‘early detection and prevention’ (32%;SUW) and ‘training of parents’ (32%;SUW). Those not commonly identified were ‘finance/budgeting’ (8%;S), ‘family engagement’ (12%;U), 'leadership’ (11%;W), ‘empowering staff’ (8%;SUW), and ‘role of language and culture’ (5%;SW). The commonality of Component Themes for all studies combined and for each of the four subgroups was very similar and is described in visual representations. Alignment with guidance and local system managers’ concerns is discussed.

**Conclusion:**

We suggest this list of Component Themes and their intended target(s) of impact be considered when updating guidance on integrated care for CYPF. Existing guidance may benefit from additional implementation support around the integration of finance across the system; leadership, empowerment, language and culture across the workforce; and embedding meaningful CYPF engagement.

**Supplementary Information:**

The online version contains supplementary material available at 10.1186/s12913-025-13345-w.

## Introduction

Integrated care has become a central feature of health system reform worldwide, with the aim of tackling the impact of increased demand and improving care through reducing the fragmentation of services [1, 2]. However, this is challenging because health and social care services are provided by various sectors and organisations, including the wider public health providers, and arrangements for delivering and funding them are complicated [1]. The World Health Organization (WHO) framework on integrated care proposes that a move towards greater integration provides opportunities to strengthen governance and co-ordination of services, empower individuals and communities, and improve the quality and efficiency of healthcare and population health [3]. Joining up pathways of care, through integrated care systems and models, has been argued as an essential goal for those who regulate, deliver and receive care worldwide [4]. While systems and models of integrated care for CYPF have been developed around the world, replication can be challenging due to variations in national, local and health service contexts, together with differences in integrated care provision for specific health conditions [5]. Whilst trust and goodwill between services and providers will be required for novel models of care to be implemented, evaluation of these new models and incorporation of young people’s healthcare preferences is needed [5].

Ideally, one would develop an integrated care system or model based on evidence for effectiveness. However, there is limited evidence for the success of integrated care for CYPF [6]. As such, integrated systems and models for CYPF have been largely designed around the health care needs for adults. There is a risk in taking this approach because there are distinct characteristics of integrated care for CYPF, due to the significant roles of family and education, and changing needs across childhood and adolescence. For CYPF, integrated care will encompass vertical integration (between primary and secondary care), horizontal integration (between health, education and social sectors, and in some cases the Voluntary, Charity and Social Enterprise (VCSE) sector), longitudinal integration (developmentally appropriate co-ordination of health and non-health services), and population integration (taking a public health focus, including health promotion strategies and preventative measures alongside clinical care) [7, 8].

To improve the evidence base for England, national programmes of local evaluations of integrated care were funded to provide answers to a number of questions including *“What are the ‘active ingredients’ (the aspects that could be replicated elsewhere to give similar results) for success?”* [9]. However, producing a robust national evidence-base, constructed from numerous locally-produced evaluations, has proven difficult [10]. Evaluators are experiencing multiple complications in pulling the evidence together in a way that will produce learning about replicable models of integrated care. Some local system managers have questioned whether, given the fact that successful integration requires tailoring to local needs, replicable models are useful [11]. Integrated care in England has progressed “in fits and starts” [12], with a lack of evidence for the generalisability and transferability of earlier examples of integrated care in England [8].

In response to this, authoritative organisations and agencies have developed generic guidance on integrated care alongside implementation resources (e.g [3, 13–18]) to support those responsible for implementing integrated care. Examples considered in this paper are those relevant to England, UK, and include general guidance for all ages (children and adults) produced by WHO [3], National Health Service for England (NHS England) [13], and the Social Care Institute for Excellence (SCIE) [14], and general guidance specifically for CYPF produced by the Royal College of Paediatrics and Child Health (RCPCH) [15]. We also considered guidance with a particular focus on CYPF engagement produced by the RCPCH [16] and guidance with a particular focus on workforce planning produced by the Local Government Association [17]. Guidance specifically for CYPF living with certain health conditions is emerging, for example WHO’s forthcoming guideline on integrated management of children in all their diversity with obesity [18]. The evidence base for these guidance documents is a combination of academic theory-based research, best practice and local system managers’ advice. A key feature of this type of guidance is the inclusion of a list of components for integrated care (variously labelled in the guidance as active ingredients, activities, best practice actions, building blocks, checklist, components, elements, enablers, guiding principles, ingredients and recipe) alongside implementation resources. Although the list of components included in general guidance on integrated care varies a little, most components are common to all. The guidance suggests all components should ideally be considered for implementation. Guidance produced by the SCIE [14] describes components as ‘enablers’ *(collaborative leadership and shared governance; people and communities as partners; population approaches; integrated workforce; digital records, data, and shared information; joint commissioning and integrated resource allocation; sustainable care provider market)* and ‘activities’ *(prevention and independence support services; multidisciplinary teams; joint assessment and care planning; community-based integrated services; care and support in a crisis; safe and timely transitions of care; integrated carer support)*. In all guidance mentioned above [3, 13–18], the importance of ‘context’ (local, i.e. place-based, and health condition) is highlighted in relation to the complexity of developing and delivering integrated care services. In some of this guidance, the issue of ‘interactions’ between components and complexity is mentioned. However, there is limited meaningful discussion in the guidance on the ‘weighting’ (or relative importance) of components by, for example, their impact on outcomes and assume this is purposeful.

Alongside this policy and practice effort to support those responsible for implementing integrated care there has been a proliferation of academic activity around the development and refinement of theories, logic models and frameworks [19]. An output of this type of activity is often a list of generic key components of integration. In a recent review of this literature [20], a list of 11 components from 36 publications were identified: *stakeholder management; adequate funding; technological connectivity; roles; governance; communication; shared vision, values and goals; context; culture; community engagement; colocation*. Although none of the included studies in this review focussed on CYPF, many of the key components map onto those which are commonly found in the guidance mentioned above. The academic literature also discusses the importance and challenges of context and complexity when trying to characterise a generic list of components of integrated care. Hughes et al [20] conclude their review with a number of policy points including a) Policies to integrate care that facilitate person-centered, relationship-based care can potentially contribute to (but not determine) improved patient experiences, and b) There can be an association between improved patient experiences and system benefits, but these outcomes of integrated care are of different orders and do not necessarily align.

The study described in this paper was carried out as part of a larger project within the NIHR School of Public Health Research that used a variety of research approaches to explore how developing Integrated Care Systems in England considered the needs of CYPF [21], to review measurement instruments for integration [22], and to understand components of integrated care for this age group. Local system managers in England, using guidance such as that mentioned above, reported specific challenges around integrating system-wide funding, trusted relationships across the workforce, and CYPF engagement [22]; these are components of integration which were commonly identified in the guidance that was available to them and which are commonly identified in the academic literature. We aimed to systematically generate Component Themes of integrated care, alongside examples of implementation, from existing systems and models for CYPF in published studies.

We aimed to assess the commonality and intended primary direct target(s) of impact (the system, users and/or workforce) of these Component Themes across included studies. We also aimed to explore whether this varied by four health conditions and one age group. We argue that the subgroups we have chosen are particularly important for integrated care as they have more direct implications for which professionals and sectors need to be integrated, both within and beyond mainstream health care services. The extended multidisciplinary team (MDT) in this context includes a wider workforce. Greater benefits are realised when neighbourhood MDTs are also integrated with wider local services, especially with education, social care and VCSE partners, to provide holistic, targeted support [23]. The subgroups we chose map well to commissioning approaches at national policy level in England. For example, NHS England have funded a number of specific integrated care pilots for early years care and are considering something similar for mental health. Learning disability and autism services, and obesity services, for CYPF are also a high priority in integrated care plans which focus on neighbourhood multidisciplinary teams [23].

In our discussion we compare our findings with that provided within authoritative guidance on integrated care for those responsible for implementing integrated care. We offer suggestions as to why local systems managers report greater challenges around implementing certain components of integration. For clarity, we did not aim to develop a new theory, logic model or framework of integrated care for CYPF. Our primary focus was on providing information to support authoritative organisations and agencies in their ongoing development of guidance on integrated care for CYPF. For example, we aimed to identify components of integration that are commonly listed in guidance but which were often missing in the existing systems and models included in this review.

## Methods

The aim of this rapid review was to identify descriptions of components of Integrated Care Systems for children, young people and their families (CYPF) from the literature. We did not set out to develop a new theory, logic model or framework of integrated care for CYPF.

A hierarchy of study designs relating to quality of description of components of an intervention, model of care or system (in this case, of integrated care) does not exist. After discussions between members of the review team, many of whom have significant expertise in reviewing, we agreed that we were not able to justify the inclusion of certain study designs over others. Therefore, we opted for an inclusive approach to study designs for this rapid review.

The identification of included studies was carried out following the Cochrane guidance for conducting a rapid review [24]. Rapid reviews have been described as a type of knowledge synthesis in which systematic review methods are streamlined and processes are accelerated to complete the review more quickly, and most efficiently, whilst retaining an acceptable degree of rigour. Rapid reviews have emerged as an efficient tool to get evidence to decision makers more quickly and are now considered part of the.

knowledge synthesis family. For this rapid review, areas we chose to streamline included the a) range of databases searched, b) use of double screening of all titles and abstracts, and c) use of double data extraction for studies identified in the update search. This is described in more detail below.

The application of this rapid review approach allowed us to identify, appraise and synthesise the available evidence in a relatively short space of time whilst considering a broad spectrum of complexities that occur due to diversities in population, health condition, implementation and contextual factors. In line with Cochrane guidance, the rapid review was registered on PROSPERO (PROSPERO record CRD42021223687) [25].

### Search strategy

We searched three electronic databases (PubMed, CINAHL and Cochrane CENTRAL) using the search terms listed in Table S1. Searches were run in two phases, first to March 2022 and then an update search to the end of 2023.

### Inclusion/exclusion criteria

Studies written in English language, conducted in high-income countries, and published from 1st January 2016 to the 31 st December 2023 were included. We used the pragmatic start date of 01/01/2016 since it was in this year that NHS England first asked all parts of England to begin planning together in new partnership. It included forming of all NHS organisations, local government and others, setting out their early thinking around Integrated Care Systems and working with partners to develop them [26]. We were also particularly interested in contemporary evidence (within last 10 years), which will be relevant to the development of new models of care.

Studies that had a focus on (a) an integrated care system or model; (b) CYP under 19 years of age but not those with a sole focus on birth centres or neonatal intensive care units; (c) health condition (general or specific); and (d) included a description of the components of an integrated care system or model were eligible for inclusion. We included peer-reviewed publications of any study design, including systematic and literature reviews.

Studies which focused on a non-integrated care system or model and/or an isolated pathway within an integrated care system or model were excluded. We did not include brief reports, commentaries or letters, conference materials, or overviews of a paper or workshop. We did not include protocols except those for randomised controlled trials where the components of the integrated care model were described in sufficient detail.

### Screening

Initially, 10% of the search results (titles and abstracts) from the first phase of searching (up to March 2022) were double screened independently by two of four members of the research team (FH-B, SH, EO-B or ES). Before we started screening, the members of the review team who were going to be involved in screening and data collection were provided with a selection of general reading on integrated care, including the WHO framework on integrated care [3], to give them a general understanding of the relevant literature. There was a 94% agreement rate between reviewers. All four reviewers met to resolve any disagreements and a short guidance document was prepared to support the screening process. The remainder of the search results from the first phase of searching were screened by EO-B or ES. Where it was unclear whether a paper should progress to the next stage based on title and abstract, it was progressed.

Following the screening of titles and abstract, full papers were retrieved where possible. Full papers were assessed for inclusion independently against the inclusion/exclusion criteria by two reviewers (EO-B or ES). Where there was disagreement or uncertainty, studies were reviewed through consensus discussion between four members of the research team (FH-B, SH, EO-B and ES). The screening of titles and abstract, and assessment of full papers, from the update search was conducted by ES and, where there was uncertainty, studies were reviewed through consensus discussion between ES and CS.

### Data extraction

Data extraction was carried out using a form piloted on a sample of five of the included studies with different study designs identified from the first phase of searching and then revised to improve usability. Extracted data included a description of integrated care components; geographical (country) location; study setting(s) (e.g. primary care and schools); target age range; target health condition or disorder; study design. There is no existing tool that critically appraises or assesses the quality of studies for describing components of interventions, models of care or systems. That said, we argue that by aiming to demonstrate the transparency of the methodology of this review, not performing critical appraisal did not jeopardise the quality of the review [27].

### Coding of components into component themes

The terminology used to describe the same type of component of integrated care can vary between studies. Furthermore, components can be grouped together under themes. In this paper, we term these Component Themes. Indeed, in the guidance on integrated care that we consider in this paper, this guidance offers recommendations under such themes, although they label them differently. For example, the guidance produced by the SCIE [14] describes these themes as ‘enablers’ and ‘activities’. We aimed to code the components of integrated care that were described in the included studies in this rapid review into one of a number of Component Themes. We did not start this process with an *a prior* list of Component Themes or a target for the number of Component Themes we would report.

The first 50 studies were divided across the reviewers and we familiarised ourselves with the completed data extraction forms. We then identified individual components of integrated care within each study and coded these under themes inductively, using codes which made sense to the individual reviewer. For example, where a component identified in a study was termed ‘training of staff’ this may have been initially coded by one of the reviewers into a theme they called ‘staff training’. During this coding period, which lasted approximately six months, the reviewers met together at weekly reflexive meetings. During these meetings, we talked about potential names of the themes we were identifying in the literature with the aim of agreeing on a set on names that we could all use. A thematic analysis approach was used to develop the list of themes for this review [28] led by ES, supported by FH-B, and conducted by one of four reviewers (ES, EO-B, FH-B and SH). Specifically, the Framework method was used [29]. We began the thematic analysis by tabulating and grouping the individual components into one of a number of prominent and recurring themes. In doing so, components from different studies that were very similar in nature were merged under a theme.

After the first 50 studies were coded, each theme was assigned an agreed name and a code number based on the number of components identified for each theme; the theme with the most components was coded as one, etc. [Note: at this early stage, we identified only 23 themes. Also, their order was slightly different to the order for the complete dataset of 170 studies]. From this point, the themes were called Component Themes. The coding process was then continued for the remaining studies (first those from our original search and then for those identified in the update search). The list of Component Themes was continually assessed at the reflexive meetings and, where deemed appropriate, adapted and expanded and differences resolved. A list of all of the identified components and The Component Themes is provided in Table S4 in the Supplementary file.

At the end of the coding period, and as a final check, the coding of components into Component Themes for all included studies identified from the first phase of searching were reviewed again by FH-B or ES. Although this was a time-consuming process, and perhaps at odds with a rapid review approach, it was considered a critical step in our methodology. This additional checking was particularly useful for components in studies that were described in little detail. As such, we were confident in having used a consistent approach to coding. The coding of studies from the update search was conducted by ES and, where there was uncertainty, studies were reviewed through consensus discussion between ES and CS.

### Coding of components for their intended target of impact

Alongside the coding of components into Component Themes, components were also coded for their intended *direct primary* target(s) of impact in terms of the system (S), users (U) and/or workforce (W). These three broad target categories were selected based on the way in which current guidance for local system managers is often presented [3, 13–18]. Indeed, the focus of one of the guidance documents we consider in this rapid review is specifically on engagement with CYPF [16] and another is specifically on workforce planning [18]. The systems category included components that directly targeted the planning and delivery of the services within and across systems of care, including health systems, education systems and the VCSE sector. As well as within and across financial and IT systems or other technical systems that enable communication. The users category included components that directly targeted service users (children and/or their families) aiming to improve their experiences across the continuum of care. It also included their (patient and public) involvement and engagement in actively improving the care they received. The workforce category included components that directly targeted the workforce involved with planning and delivery, including front-line staff. This impact could include training and support around skills and knowledge in relation to the operations of the system or model or behavioural skills which promote implementation and job satisfaction. Workforce, in this context, includes the wider workforce across different sectors including healthcare, social care, education, and also the VCSE sector.

The process of categorisation of components for their intended target(s) of impact was discussed by the reviewers during their weekly reflexive meetings during the coding period. At the end of the coding period, and as a final check, the coding of components for their intended target(s) of impact were reviewed again by FH-B or ES. The coding of studies from the update search was conducted by ES and, where there was uncertainty, studies were reviewed through consensus discussion between ES and CS.

### Quality assessment

Our initial plan was to quality assess the included studies. However, given the mixture of study designs included in this rapid review, and the fact that we did not judge one type of study design a stronger level of evidence about components of integrated care than another type of study design for this rapid review, we deemed that it would not be helpful to conduct a quality assessment.

### Subgroup analysis

Subgroup analysis was planned for five pre-specified subgroups; mental health, learning disability and autism, obesity, early years, and dental health. These subgroups were selected based on public health priorities for CYPF in England, described above and promoted by the Academy of Medical Sciences [30]. However, subgroup analysis was not conducted for dental health because only two studies were identified which focussed on this public health priority.

#### Mapping of components identified in six authoritative guidance documents on integrated care onto the 25 Component Themes identified in this rapid review

Two members of the research team (FHB and CS) assessed six authoritative guidance documents [3, 13–17] for inclusion of components of integrated care and mapped these onto the 25 Component Themes identified in this rapid review. Any disagreements were resolved through discussion.

### Changes from protocol

We made three changes to the protocol during the project. First, we did not search the grey literature as planned because we found an unexpectedly large number of peer reviewed studies from the electronic database searches. Second, we did not assess the risk of bias (quality) of included studies. Finally, we did conduct subgroup analysis for four of the five pre-specified public health priority groups given the greater than expected number of studies we identified for these subgroups.

## Results

### Study selection

**Fig. 1:**
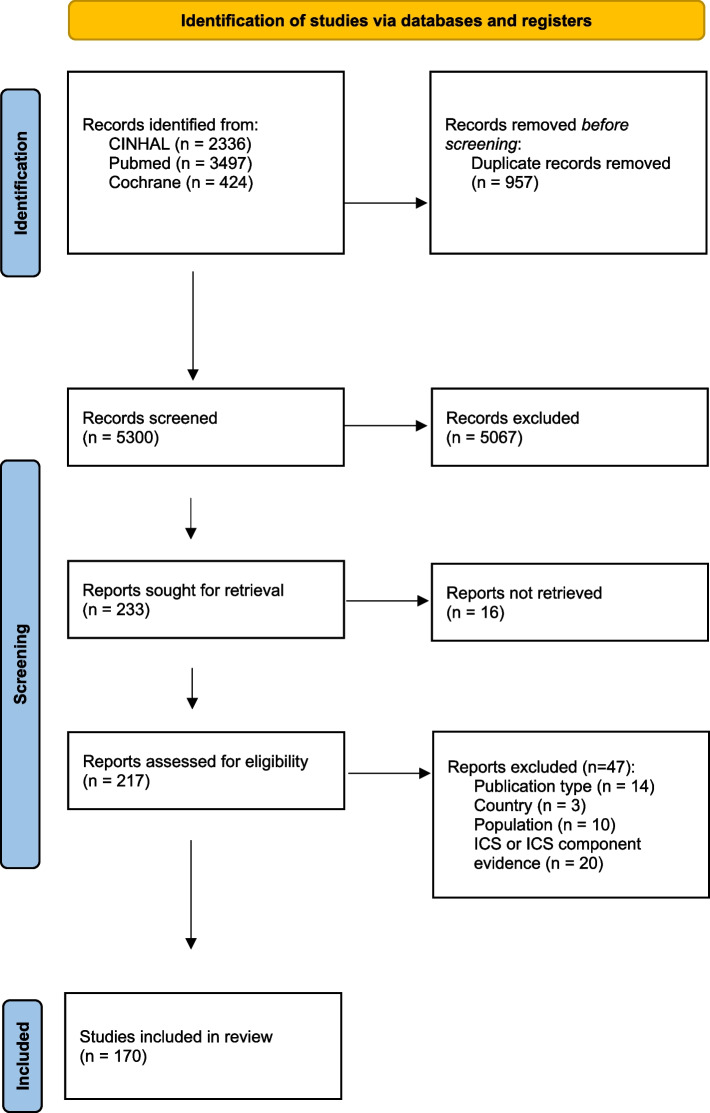
PRISMA diagram of the study selection process for components of integration for CYPF

A total of 5,300 records were screened and 217 full-texts were assessed potentially eligible based on the title and abstract. Of these, 170 studies [31–200] met the inclusion criteria and were included in the review. The characteristics of the integrated care systems and models described in these 170 studies is presented in Table S2; a list of the component(s) of integration they contained and their intended target(s) of impact, country where the study was conducted, study setting, target population, and target health condition/disorder. The list of studies excluded at the full paper selection stage (*n* = 47), and reasons for their exclusion, is presented in Table S3. A flow diagram of the study selection process is shown in Fig. 1, in line with PRISMA guidance [201].

### Study characteristics

#### Country of study

Included studies were conducted in the US (*n* = 99), Canada (*n* = 16), Australia (*n* = 12), UK (*n* = 12), multiple countries (*n* = 9), The Netherlands (*n* = 4), Germany (*n* = 3), Norway (*n* = 3), Belgium (*n* = 2), Estonia (*n* = 2), Italy (*n* = 2), Austria (*n* = 1), Ireland (*n* = 1), New Zealand (*n* = 1), Poland (*n* = 1), Spain (*n* = 1) and Sweden (*n* = 1) (Table S3).

#### Study setting

Most studies described an integrated healthcare system or model which included more than one setting or which was set solely across primary care settings. Two studies, both of which focused on the early years, took place across public health settings in the local community [34, 52]. Four studies took place solely across school educational settings [62, 78, 105, 149] and a further study took place solely across early childhood educational settings [76]. Six studies took place across a mix of settings including school educational settings [39, 55, 103, 141, 172, 186]. Two studies, both of which focussed on mental health, took place across a mix of settings including social services settings [92, 94] and one included social rehabilitation services settings [169]. Two studies took place across a mix of settings including alcohol and drug/substance abuse services [80, 94]. One study included youth welfare services [39] and one study included sexual and reproductive health services [80]. A number of other studies described integrated care systems or models that were cross-sectoral or inter-sectoral although it was unclear as to the details of the sectors involved.

#### Age range targeted

The age range of children included varied by study, but most studies had an upper limit of 18 years; a few studies included individuals up to 21 or 25 years of age. Twenty-two studies were solely focussed on the early years (defined here as 0 to 5 years old) and a further 15 studies that involved children of a wider age range included an early years focus as part of their analysis.

#### Health condition/disorder targeted

The health condition/disorder targeted by each study ranged from general (*n* = 25) to specific health conditions including mental health (*n* = 79); long-term complex needs (*n* = 32); learning disabilities and autism (*n* = 17); obesity (*n* = 9); asthma (*n* = 4); dentistry (*n* = 3); special needs (*n* = 3); cardiology (*n* = 2); cerebral palsy (*n* = 2); diabetes (*n* = 2); eating disorder (*n* = 2); oncology (*n* = 2); post-intensive care syndrome (PICS) (*n* = 2); and other conditions which only one study focussed on. For some studies, more than one health condition/disorder was targeted, most commonly ‘mental health’ plus ‘learning disabilities and autism’, ‘mental health’ plus ‘eating disorders’ and ‘mental health’ and ‘long-term complex needs’.

#### Study design

The majority of included studies did not include an evaluation of the integrated care system or model described. Seven studies conducted an evaluation [37, 50, 94, 130, 164, 187, 200]. These evaluations were qualitative and/or quantitative in nature and focussed on a selection of process measures. Only one study [196] reported the results of a randomised controlled trial (RCT) which included outcome measures of effectiveness. In addition, two of the included studies were protocols for RCTs that were fully described in a journal article [118, 132]; these trials will provide important evidence on effectiveness in due course.

##### Overview of component themes

We identified 25 high-level Component Themes for integrated care (Table 1), each consisting of between four to 179 different components. A total of 1,057 components were identified; some of these contributed to two or more Component Themes. The individual components included in each Component Theme are presented in Table S4 and a brief description of the Component Themes is provided in Table S5.

**Table 1:**
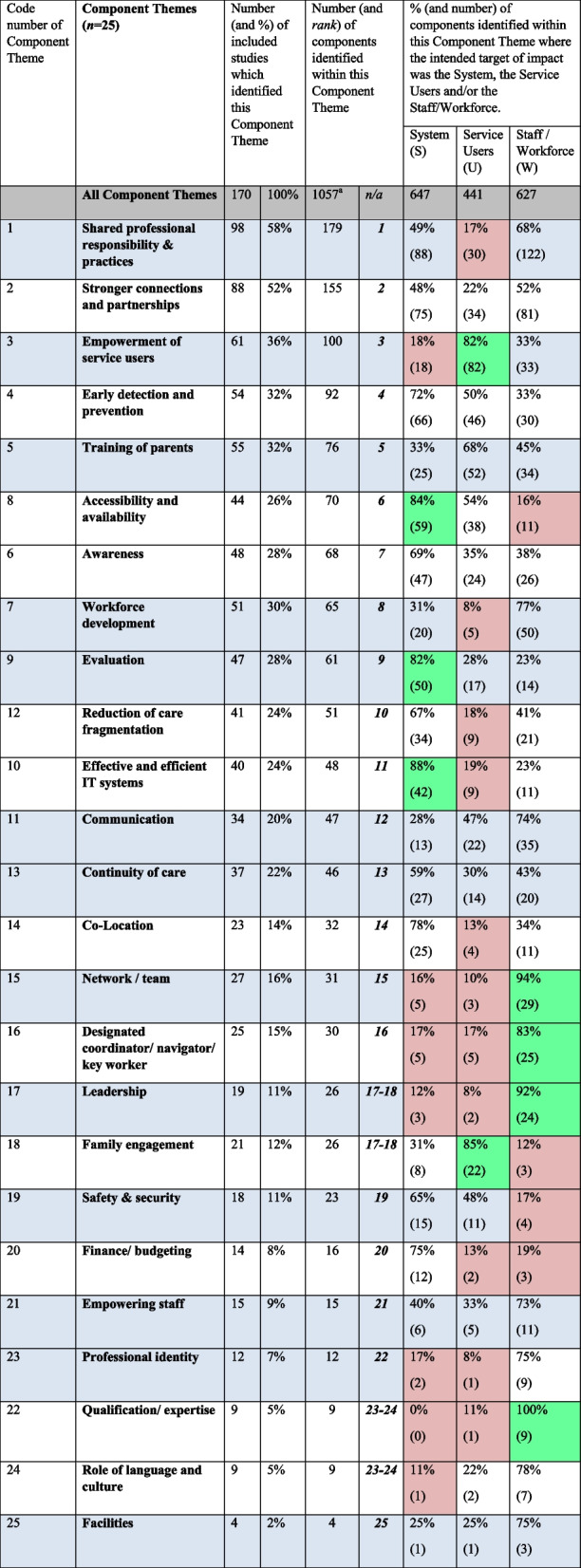
The 25 Component Themes of integration for CYP identified from 170 studies, the number (and rank) of their components of integration and their intended target(s) of impact (system, user and/or workforce)

^a^Some components contributed to more than one Component Theme (234 cases, see Table S2)

None of the studies identified all Component Themes (median 5, range 1–16). Table 1 presents a) the number (and %) of included studies which identified specific Component Themes and b) the number of different components identified within each Component Theme (and rank). Table 1 also includes information on the % (and number) of components in each Component Theme where the intended primary direct target of impact (simply called intended target of impact from this point) was the System, User and/or Workforce. For each intended target of impact, where the % of components in a Component Theme contributed a high proportion (≥ 80%), these cells are highlighted in green. Conversely, where the % of components contributed a low proportion (≤ 20%), these cells are highlighted in brown.

##### Commonality of component themes

The most commonly identified Component Themes were ‘shared professional responsibility and practices’ (identified in 58% of studies and from 179 components; intended targets of impact were primarily S and W), ‘stronger connections and partnerships’ (52%, 155, S, U and W), ‘empowerment of service users’ (36%, 100, U), ‘early detection and prevention’ (32%, 92, S, U and W) and ‘Training of parents (32%, 76, S, U and W). 63% of studies included Component Theme number 1 and/or number 2; 91% of studies included one or more Component Themes numbers 1 to 5. Component Themes that were not commonly identified were ‘finance/budgeting’ (8%; S), ‘family engagement’ (12%; U),'leadership’ (11%; W), ‘empowering staff’ (8%, S, U, W), and ‘role of language and culture’ (5%, S, W).

None of the studies included components which mapped onto all 25 Component Themes. As mentioned above, a brief description of these Component Themes is presented in Table S5 and a list of the individual components from included studies that contributed to each of these Component Themes is presented in Table S4. The components identified for each study are included in Table S2.

##### Intended target(s) of impact of component themes

**System**

The intended target of impact for around half of the components in the ‘Shared professional responsibility & practices’ and ‘Stronger connections and partnerships’ Component Themes was the system. The three Component Themes which included a high proportion of components where the intended target of impact was the system were ‘Effective and efficient IT systems’ (88% of components identified from 24% of studies), ‘Accessibility and availability’ (84% of components identified from 26% of studies) and ‘Evaluation’ (82% of components identified from 28% of studies).

**Users**

The intended target of impact for a low proportion of the components in the ‘Shared professional responsibility & practices’ and ‘Stronger connections and partnerships’ Component Themes was the users. Two Component Themes which included a high proportion of components where the intended target of impact was the users were ‘Family engagement’ (85% of components identified from 12% of studies) and ‘Empowerment of service users’ (82% of components identified from 9% of studies). Almost half (*n* = 11) of the Component Themes included a low proportion of components where the intended target of impact was the users.

**Workforce**

The intended target of impact for around two thirds of the components in the ‘Shared professional responsibility & practices’ Component Theme, and around half of the components in ‘Stronger connections and partnerships’, was the workforce. Four Component Themes which included a high proportion of components where the intended target of impact was the workforce were ‘Qualification/expertise’ (100% of components identified from 5% of studies), ‘Network/team’ (94% of components identified from 16% of studies), ‘Leadership’ (92% of components identified from 11% of studies), ‘Designated coordinator/navigator/key worker’ (83% of components identified from 15% of studies).

Visualization was used to map data onto visual dimensions to create a pictorial representation.

##### Subgroup analysis: mental health, learning disabilities and autism, obesity and the early years

We conducted the same type of analysis as we have presented above (for all studies) for subgroups of studies that focussed on mental health (*n* = 79 studies), learning disabilities and autism (*n* = 17 studies), and obesity (*n* = 9 studies) and the early years (*n* = 37 studies). These studies are colour coded in Table S2, the reference list, and Table 2 to improve readability. The results are presented in Table 2, alongside those for all studies combined for reference. Our ambition to include dental health as one of our pre-selected subgroups was not realised as we only identified two relevant studies.

**Table 2:**
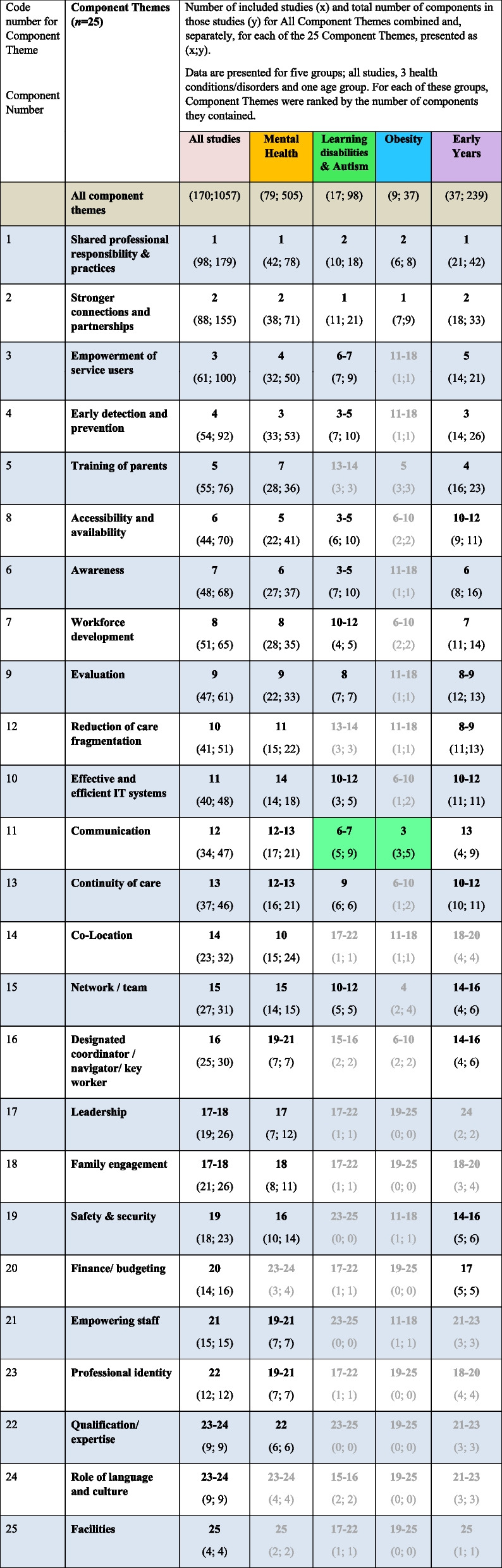
Rank of number of components that drove integration for each Component Theme for all included studies (for reference) and for 3 health conditions/disorders and one age category

Cells with less than 5 components were ranked but are greyed and were not considered for further reporting. Where the ranking was ≥ or ≤ 5 ranking points from the ranking for all studies, cells are highlighted green or brown, respectively

##### Commonality of component themes by subgroup

The results for commonality of Component Themes by subgroup are presented in Table 2; cells with less than 5 components were ranked but are greyed and were not considered for further reporting. Where the ranking was ≥ or ≤ 5 ranking points from the ranking for all studies, cells are highlighted green or brown, respectively.

With just two exceptions, the rank order was similar across health condition/disorder and age group, compared with all studies. The Component Theme ‘communication’ was ranked higher for integrated care systems and models focussing on obesity and on learning disabilities & autism. Given the relatively low number of components for many Component Themes by subgroup we resisted commenting on cross subgroup results.

##### Intended target(s) of impact of Component Themes by subgroup

The intended target of impact (system, users and workforce) for the components in each Component Theme, by subgroup, is presented in Supplementary Tables 6a-6c. The primary intended target(s) of impact of the Component Themes for the four subgroups was the same as for all studies. The same analysis for all studies is included for reference and the order of Component Themes in these tables follows the rank order for all studies. As in Table 2, cells with less than 5 components were ranked but are greyed and were not considered for further reporting. Where the ranking of Component Themes for a subgroup was ≥ or ≤ 5 ranking points away from the ranking of Component Themes for all studies, cells are highlighted green or brown, respectively.

**System**

There was no difference in the rank order of all studies compared with those that had a focus on mental health. For those studies with a focus on learning disabilities & Autism, the ‘Network/team’ Component Theme was ranked higher (2 of 5 components) and the ‘Effective and efficient IT systems’ Component Theme was ranked lower (3 of 5 components). The data for studies which focussed on obesity was mostly greyed out due to low numbers of components in cells. For studies with a focus on the early years, the ‘Awareness’ (14 of 16 components) and ‘Communication’ (4 of 9 components) Component Themes were ranked higher and the ‘Safety & security’ Component Theme was ranked lower (1 of 6 components).

**Users**

For those studies with a focus on mental health, the ‘Designated coordinator/navigator/key worker’ (2 of 7 components) and ‘Qualification/expertise’ (1 of 5 components) were ranked higher. For studies with a focus on learning disabilities & autism, the ‘Evaluation’ (3 of 7 components), ‘Effective and efficient IT systems’ (2 of 5 components) and ‘Shared professional responsibility & practices’ (2 of 18 components) were ranked higher. Where obesity was the focus of studies, the ‘Shared professional responsibility & practices’ was ranked higher (2 of 8 components). For studies with a focus on the early years, the ‘Finance/budgeting’ Component Theme was ranked higher (2 of 5 components) and the ‘Evaluation’ component Theme was ranked lower (2 of 13 components).

**Workforce**

For those studies with a focus on mental health, the ‘Empowering staff’ (6 of 7 components) was ranked higher and ‘Communication’ (15 of 21 components) was ranked lower. For studies with a focus on learning disabilities & autism, ‘Early detection and prevention’ (5 of 10 components), ‘Effective and efficient IT systems’ (2 of 5 components) and ‘Accessibility and availability’ (3 of 10 components) were ranked higher. The data for studies which focussed on obesity was most greyed out due to low numbers of components in cells. For studies with a focus on the early years, ‘Effective and efficient IT systems’ (4 of 11 components) and ‘Safety and security’ (2 of 6 components) were ranked higher; ‘Continuity of care’ (3 of 11 components), ‘Reduction of care fragmentation’ (3 of 13 components) and ‘Awareness’ (3 of 16 components) were ranked lower.

**Fig. 2:**
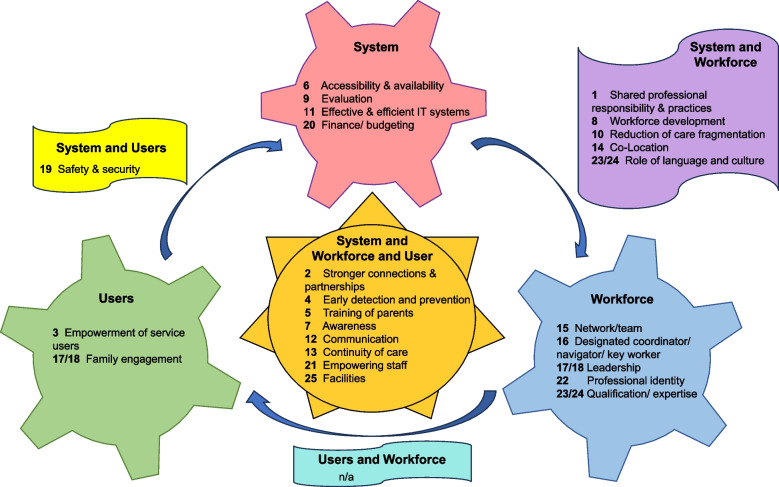
Visual representation of the commonality of Component Themes of integrated care by rank order (1 to 25) and their primary target of intended impact (system, workforce and/or users) reported in 170 studies: Children of all ages and for all health conditions

Visualization was used to map data onto visual dimensions to create a pictorial representation for mental Health (Fig. S1), learning disabilities and autism (Fig. S2), obesity (Fig. S3) and the early years (Fig. S4). Given that the primary intended target(s) of impact of the Component Themes for the four subgroups was the same as for all studies, Component themes are represented in the same colour shapes in Fig. 2 and Figs. S1- S4.

The list of components included in the four examples of general guidance documents on integrated care we considered in this paper [3, 13–15] mapped very well onto the 25 Component Themes identified in this rapid review. The only Component Themes that were not included were the role of language and culture in two of the four documents, finance/budgeting in one document, and evaluation in one document (Table 3).

**Table 3:**
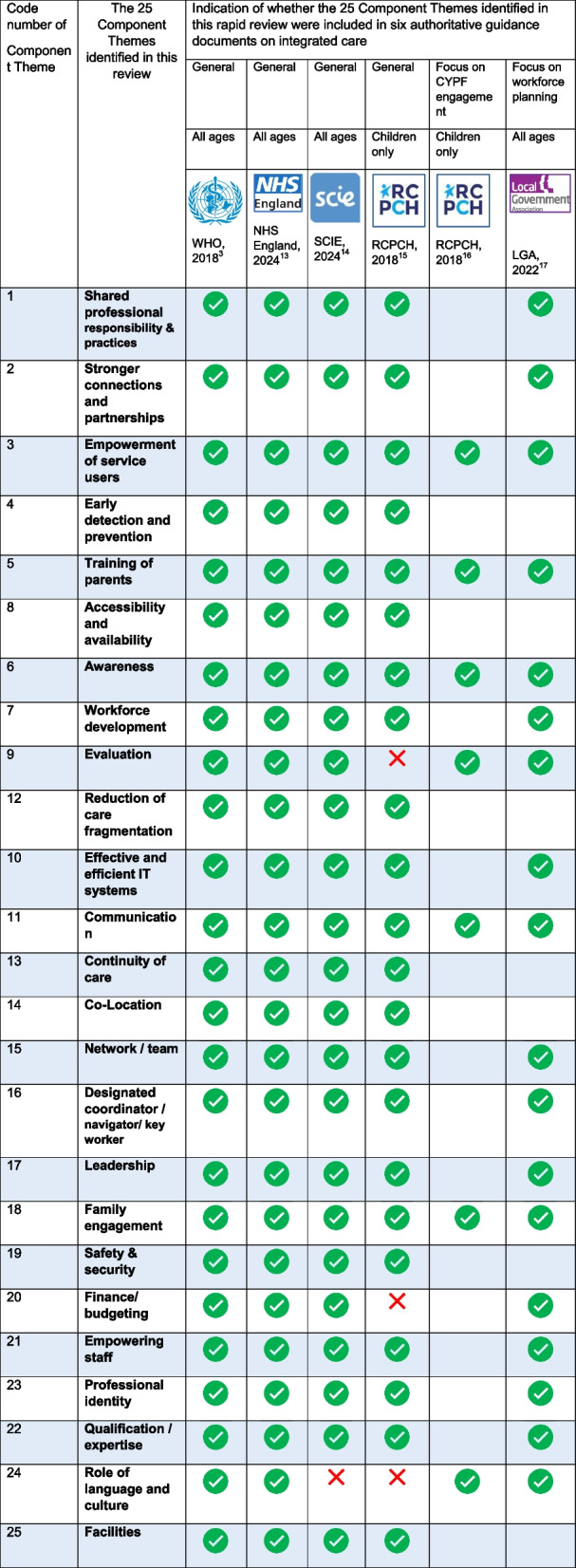
Indication of whether the 25 Component Themes identified in this rapid review were included in seven authoritative guidance documents on integrated care

The list of components included in the two examples of guidance documents we considered in this paper with a particular focus on CYPF engagement [16] and workforce planning [17] mapped very well onto the relevant Component Themes identified in this rapid review (Table 3).

## Discussion

This systematic and in-depth review of existing integrated care systems and models for CYPF has generated a comprehensive list of Component Themes and their intended target(s) of impact. This review expands and strengthens the body of evidence on CYPF integrated care components described in the existing literature, minimising gaps in knowledge that may occur when using single system and models and providing a more comprehensive set of components described with more granularity, particularly in relation to the workforce and users. This review provides practical policy-relevant information to support authoritative organisations and agencies who are responsible for producing guidance on integrated care for CYPF and for those delivering integrated care within local systems, allowing flexibility for context-specific priorities.

Support in the form of guidance resources produced by authoritative organisations and agencies is available for those responsible for implementing integrated care for CYPF at a local level e.g. [3, 13–17]. A key feature of this type of guidance is the inclusion of a list of components of integrated care alongside implementation resources. Although the list of components included in these guidance resources varies, most components are common to all. This rapid review and thematic analysis systematically generated 25 Component Themes of integration, and their intended target(s) of impact, from 170 existing integrated care systems and models for CYPF. Although these Component Themes generally map well onto the components listed in the guidance we considered in this paper, we generated a slightly longer list. We generated five Component Themes with a focus on the workforce (‘Network/team’, ‘Leadership’, ‘Empowering staff’, ‘Professional identity’ and ‘Qualification/expertise’) and two Component Themes with a focus on the workforce alongside the system (‘Workforce development’ and ‘Role of language and culture’) which are usually categorised under an ‘umbrella’ component of ‘Workforce development and training’ in guidance. Where these Component Themes were identified in studies, they were considered important individual components of integrated care for CYPF. The reason for this may relate to one of the distinct characteristics of integrated care for CYPF which is the important role (or potentially important role) of the wider health workforce in sectors beyond mainstream health care services.

Staff working in the education sector (such as schools, nurseries and other early years childcare settings), and the social care, youth justice, youth welfare (including alcohol and drug/substance abuse services) and VSCE sectors, can be considered part of the wider health workforce to support integrated care for CYPF. Local authority/government staff, and staff in the VSCE sector, who work within community settings/programmes which target CYPF who are at greater risk of disadvantage and/or work in places of relative high deprivation should also be considered part of the wider health workforce team; examples for England include ‘Family Hubs’ and ‘Start for Life programme’ [202]. With this context, those working with CYP who are generally disengaged with school (but may be engaged with local youth clubs and groups) or those working with CYP programmes during school holidays (an example for England is the ‘Holiday Activity and Food programme’ [203]) may have a particularly important role, if only with regard to reporting and signposting. There is some evidence that integration of different sectors across the wider health workforce is particularly challenging, in part due to their different use of ‘language’ and cultures [204, 205]. In addition, there are ethical challenges when integrating care because of the blurring of boundaries of care domains [206]. This can risk undermining the locus of responsibility for care decisions via confusion about who has ownership of specialist knowledge where domains overlap. We suggest there may be a risk of the individual workforce-related Component Themes that we identified in this review being underrated or lost when considered under an umbrella workforce component for integrated care for CYPF, particularly ‘Leadership’, ‘Empowering staff’ and ‘Language and culture’.

Similarly, we generated two Component Themes with a focus on users (‘Empowerment of service users’ and ‘Family engagement’) and one Component Theme with a focus on users alongside the workforce and the system (‘Training of parents’) which are usually categorised under an umbrella component of ‘Service Users’ in guidance. One of the distinct characteristics of integrated care for CYPF is the significant role of family members. Implementation plans should consider family perspectives about the quality of integrated care for children’s services and understand the essential components that are most important to them [172]. This qualitative study [172] found four essential components: that the key health-worker understood the health needs of the family in context; that professionals involved children and caregivers in treatment; that holistic care that supported the family unit was provided; and that families experienced coordination across health, social, and education systems. Meaningful ongoing engagement with children and young people, and also with their families, during the planning of services as part of the process of continuous improvement should be embedded in integrated care for CYPF [207, 208]. Moving forward, embedding CYPF involvement (not just engagement) in the planning of services will most likely improve the quality of care [209]. We suggest there may be a risk of the individual user-related Component Themes that we identified in this review being underrated or lost when considered under an umbrella service user component for integrated care for CYPF, particularly CYPF involvement and engagement.

None of the studies in our review identified all 25 Component Themes; the median number was five with a range of one to 16. Guidance is purposively high-level and suggests all components should ideally be considered for implementation with no indication that some are more important than others and no indication of any important interaction effects between components. The main reason for this approach is the fact that there is limited evidence (of moderate quality) of effectiveness of individual components, and their interactions, due to the complexity of integrated care [6, 195, 210] and also challenges with measurement tools to assess outcomes [23]. However, theory and logic models, and general clinical consensus, which underscore the rationale for integrated care, generally agree that all components listed in guidance are important and require translation for the local context [11]. These components have been called ‘truisms’ by Goodwin [211] who suggests that although they give you an understanding of the outcome domains that matter when evaluating the difference between success and failure, they don’t solve the problem of the practical tools and approaches that might be used in specific contexts to build or replicate such capabilities. Indeed, one of goals of the study presented in this paper (and the wider project within which it sits) was to make a useful contribution to the work of those who are responsible for updating guidance on integrated care for CYPF.

The commonality of Component Themes and their intended target(s) of impact for all 170 included studies are described in a visual mapping (Fig. 2); ‘Shared Professional Responsibility’ and ‘Stronger Connections and Partnerships’ were most commonly identified. This is reflected in their prominence in guidance alongside other Component Themes, with a small number of exceptions worthy of discussion. The Component Theme ‘Evaluation’ was ranked nine (of 25) in the number of components we identified. Ongoing assessment of the impact of integrated care systems or models, using routinely collected administrative and clinically generated healthcare data, enables healthcare teams to learn and to improve services as part of continuous improvement [212]. Although ‘evaluation’ is not a specific component of integrated care as such, we suggest it is worthy of inclusion in guidance as a mechanism to drive improvements in the quality of integrated care for CYPF.

Some specific Component Themes which are included in guidance were not commonly identified in the existing integrated care systems and models for CYPF that we reviewed. Three individual workforce-related Component Themes (‘Leadership’, ‘Empowering staff’ and ‘Language and culture’) and one user-related Component Theme (‘Family engagement’) were not commonly identified; these Components Themes are discussed above. In addition, the integration of ‘Finance and budgeting’ across the system was not commonly identified. The Audit Scotland Report framework [213] stressed the need for integrated finances and financial planning to support integration of the service. An evidence review of integrating funds for health and social care found that the primary barrier was the difficulty of implementing financial integration, despite the existence of statutory and regulatory support; even where funds were successfully pooled, budget holders’ control over access to services remained limited [214]. We do not suggest that the integration of finance and budgeting is more challenging for CYPF services compared with adult healthcare. However, we do suggest that guidance considers the different set of wider health sectors beyond mainstream health services that can contribute to successful integrated care for CYPF at different ages. One potential challenge for individual organisations of successful integrated care, assuming a theoretical overall cross sector budget envelope, is a shift in funding to meet the needs of CYPF from hospital to primary care to public health services. Addressing this dilemma to the satisfaction of all organisations involved, regardless of their power [215], will be important for the successful integration of finance and budgeting. We suggest future research that focusses on power analysis in integrated care for CYPF would provide useful insights in this regard.

The commonality of Component Themes and their intended target(s) of impact for included studies in four subgroups, two health conditions (mental health and obesity), one disorder (learning disabilities & autism) and one age group (the early years), compared with all studies, was generally the same. However, there was some indication that ‘Communication’ was more commonly identified in studies with a focus on Learning disabilities & Autism and Obesity compared with all studies. Our findings for all studies compared with subgroups should be viewed with a degree of caution because the number of components was variable across subgroups and in some cases (particularly for obesity studies) very small. However, readers with a specific interest in one of these subgroups may find the results of interest.

A number of Component Themes of integrated care that are included in guidance considered in this paper [3, 13–17] and were found to be of particular concern in terms of their adoption for local system managers in England [22] were the same as those not commonly identified in this rapid review, specifically integrating system-wide funding (‘Finance/budgeting’), trusted workforce relationships ('Leadership’; ‘Empowering staff’; ‘role of language and culture’), and CYPF engagement (‘Family engagement’). Similar findings around the challenges faced by local system managers when integrating finance and budgets (in general, not specifically for CYPF) have been reported from two focus groups conducted in the US [216] and a workshop that was part of the European Healthcare Design Conference 2022 [217]. The focus groups conducted in the US [216] also highlighted the need for education of the current and future workforce on the principles of culturally and linguistically competent integrated care to help advance health equity alongside the need to focus on paediatric care integration (especially in partnership with schools).

With regard to trustful workforce relationships, we did not identify trust or trusted relationships as a Component Theme in our review. However, when we examined the individual coded components we noted 16 components from 12 studies that mentioned trust as an important factor, and specifically mutual trust between different parties (highlighted in Table S2). These components were identified across a range of Component Themes. Reference to trust referred to trust between different members of the workforce in six components and trust between users and the workforce in another six components. Two components targeting users were described as ‘Feeling safe and trusted’. ‘Trustful’ was also identified as a key value (or ingredient) on the personal level (100% relevance score) and professional level (94% on relevance score) for integrated health in a Delphi qualitative study [218]. In this study, ‘trustful’ was described as *Enabling mutual trusting between users, informal carers, communities, professionals and organizations, in and across teams*. We suggest future research that focusses on trust in integrated care for CYPF, and particularly the importance of trusted adults who provide healthcare support for youths, would provide useful insights.

### Implications for policy and practice

We suggest that authoritative organisations and agencies, when updating their guidance on integrated care for CYPF, consider the list of 25 Component Themes and their intended target(s) of impact identified in this review and also the following suggestions:

1. Consider how they label components and work towards an agreed core set of terminology.
2. Provide an indication of whether all components of integration they list are equally important and whether there are any important interactions between components;
3. Include clarity around whether subcomponents that are included under an ‘umbrella’ component should be considered independently or as a ‘toolbox’ of options;
4. Describe in more detail the range (or set) of sectors beyond healthcare and schools, including the VSCE sector and public health programmes run by (or for) local authorities, who may play an important role in integrated care for CYPF;
5. Consider additional implementation support (perhaps coaching and mentoring) around the integration of finance across the system; leadership, empowerment, language and culture across the workforce; and embedding meaningful CYPF engagement.

Integrated care is high on the health policy agenda for many countries. Recently (July 2025), the UK Government published a landmark policy document ‘Fit for the Future: 10-year health plan for England’ [219]. It focuses on three fundamental shifts in health care, including ‘from hospital to community’ and ‘from sickness to prevention’. Central to the implementation of these shifts is better integrated care, both within the NHS and between the NHS, local government and voluntary sector, and creating a new workforce model. The findings and recommendations presented in this paper provide new information which could support the proposed reforms in integrated care for children, young people and families.

### Strengths and limitations

One of the strengths of this study is the systematic and in-depth methods used to generate integrated care components and their intended target(s) of impact for CYPF. It adds value by expanding the body of knowledge on CYPF integrated care components described in current literature. The study is purposefully focussed on providing support for authoritative organisations and agencies as they produce and update their guidance on integrated care for CYPF. As such, another strength of our study was the approach we took to provide practical policy-relevant information to those who produce this guidance and, in turn, support local system managers who are responsible for delivering integrated care for CYPF.

There are a number of limitations of this study. First, our source of data was information we identified in published studies and authors may not have included a description of all components in their published articles. Different terminology used within included studies regarding what was meant by integrated care and integration was challenging in some cases; this is one example of uncertainty which was discussed at the weekly reflexive meetings. Second, our research team may have overlooked a description of a component in a published article or may have misinterpreted a description of a component incorrectly during data extraction. Third, we acknowledge that there was an inevitable degree of subjectivity to our coding process (of Component Themes and intended target(s) of impact) process. We anticipated these limitations at the outset and developed methods to limit errors and inconsistencies. Fourth, a potential limitation was the use of statistical analysis and the presentation of ‘significant’ findings, given the nature of the data under review. We chose instead to look at trends in rankings of Component Themes for all studies. When we did this analysis by their intended target(s) of impact, we were cautious by only labelling Component Themes as containing a ‘high’ or low’ proportion of components if they were in the top or bottom 20%. When we conducted this analysis for subgroups by health condition or age group we were cautious when identifying differences between a subgroup compared with all studies (the reference) by a) ignoring cells containing < 5 components b) only labelling Component Themes as containing a ‘higher’ or lower’ proportion of components if they were ranked at least 5 ranking points away from the ranking for all studies.

Other limitations of this study relate to the risk of readers misinterpreting its findings. First, it is important to understand that the commonality of Component Themes presented is not indicative of their relative ranking/weighting in terms of impact or effectiveness. Indeed, Component Themes (variously labelled) in guidance are not ranked or weighted. Second, it is likely that the impact and effectiveness associated with the successful integration of a Component Theme may vary depending on its interaction with other Component Themes and also the context (local area where implemented). Again, interaction effects between Component Themes (variously labelled) in guidance are not discussed. Rather, guidance presents Component Themes as being individually important and suggest integrated care systems should aim to include all of them.

## Conclusion

We suggest the list of 25 Component Themes and their intended target(s) of impact identified in this review of existing integrated care systems and models for CYPF (from 170 studies) be considered when authoritative organisations and agencies update their guidance for local system managers. Although this list aligns well with current guidance that we considered in this paper [3, 13–18], it includes some individual workforce and user-related Component Themes which are usually cited as subcomponents under a single workforce or user umbrella component. Also, compared with the general guidance for all age groups (children and adults), specific Component Themes relating to CYPF were identified, including ‘Family engagement’.

Compared with the guidance we considered [3, 13–17], our findings highlight the fact that none of the studies identified all Component Themes (median 5, range 1–16). We suggest that an indication of which components (and subcomponents) are more critical than others (if that is true) and whether the successful integration of some components requires the integration of other components (i.e. interaction effects, if that is true) would be helpful. We recommend that future investigation explores the impact of interaction effects and transition care which may be more than the sum of it’s parts. We appreciate the fine balance between usability of guidance and overly complicated messages but our findings suggest that local system managers may be misinterpreting the guidance as a toolbox of optional components that can be implemented in different combinations and with different degrees of effort. We suggest than one of the messages from our findings is that there is a need to breakdown service user involvement/engagement in order to do justice to the various components within this and that there is a risk of underutilising the power of the service user voice in planning and the continuous improvement of integrated care.

Some specific Component Themes that are commonly included in the guidance we considered [3, 13–17] were not commonly identified in the existing integrated care systems and models for CYPF we reviewed. First, the integration of ‘Finance and budgeting’ across the system. Second, three individual workforce-related Component Themes; ‘Leadership’, ‘Empowering staff’ and ‘Language and culture’. Third, meaningful ‘Family engagement’. These aspects of integration were also identified as particular challenges by local system managers. We suggest updated guidance may benefit from additional implementation support, perhaps through coaching and mentoring, around these three aspects of integration.

Our findings for subgroups based on health condition/disorder (mental health, learning disabilities & autism, obesity) or age group (early years) compared with all studies should be viewed with a degree of caution because the number of components was variable across subgroups and sometimes (particularly for obesity) very small. In general, there were no differences between the findings for subgroups compared with all studies. However, readers with a specific interest in one of these subgroups may find these results of interest.

## Supplementary Information

Supplementary Material 1.

Supplementary Material 2.

## Data Availability

No datasets were generated or analysed during the current study.

## Abbreviations

ICS: Integrated Care System
CYPF: Children and Young People and Families
NHS: National Health Service
WHO: World Health Organisation
