# Supplementary material for: Commonality of 25 component themes of integrated care for children: rapid review of 170 models
Source: BMC Health Serv Res. 2025 Oct 8;25:1332. doi: 10.1186/s12913-025-13345-w (PMC12505997; doi:10.1186/s12913-025-13345-w)
Supplement: Supplementary file 1 — Supplementary Material 1. [file 12913_2025_13345_MOESM1_ESM.pptx]

## Slide 1
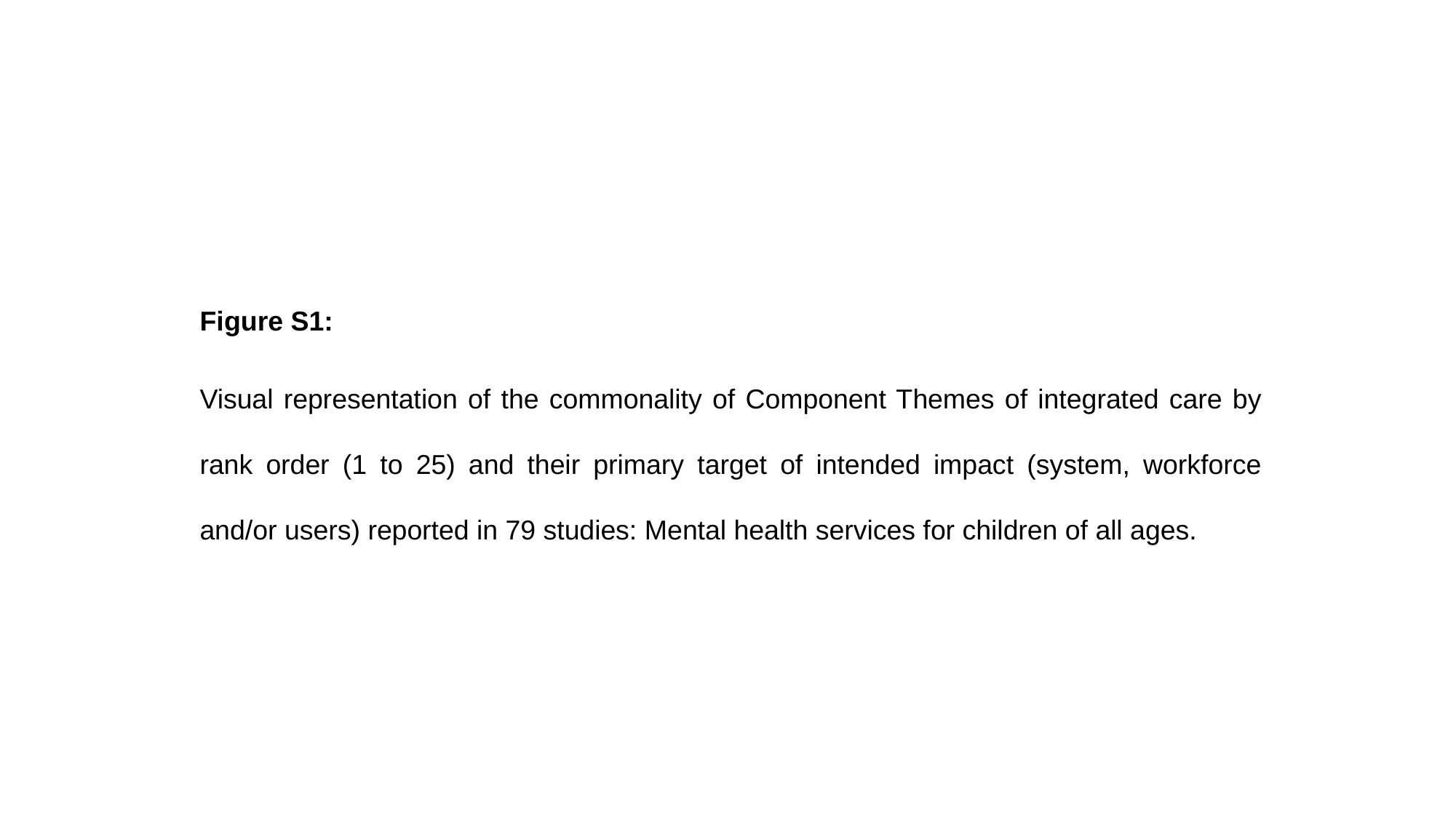

Figure S1:
Visual representation of the commonality of Component Themes of integrated care by rank order (1 to 25) and their primary target of intended impact (system, workforce and/or users) reported in 79 studies: Mental health services for children of all ages.

## Slide 2
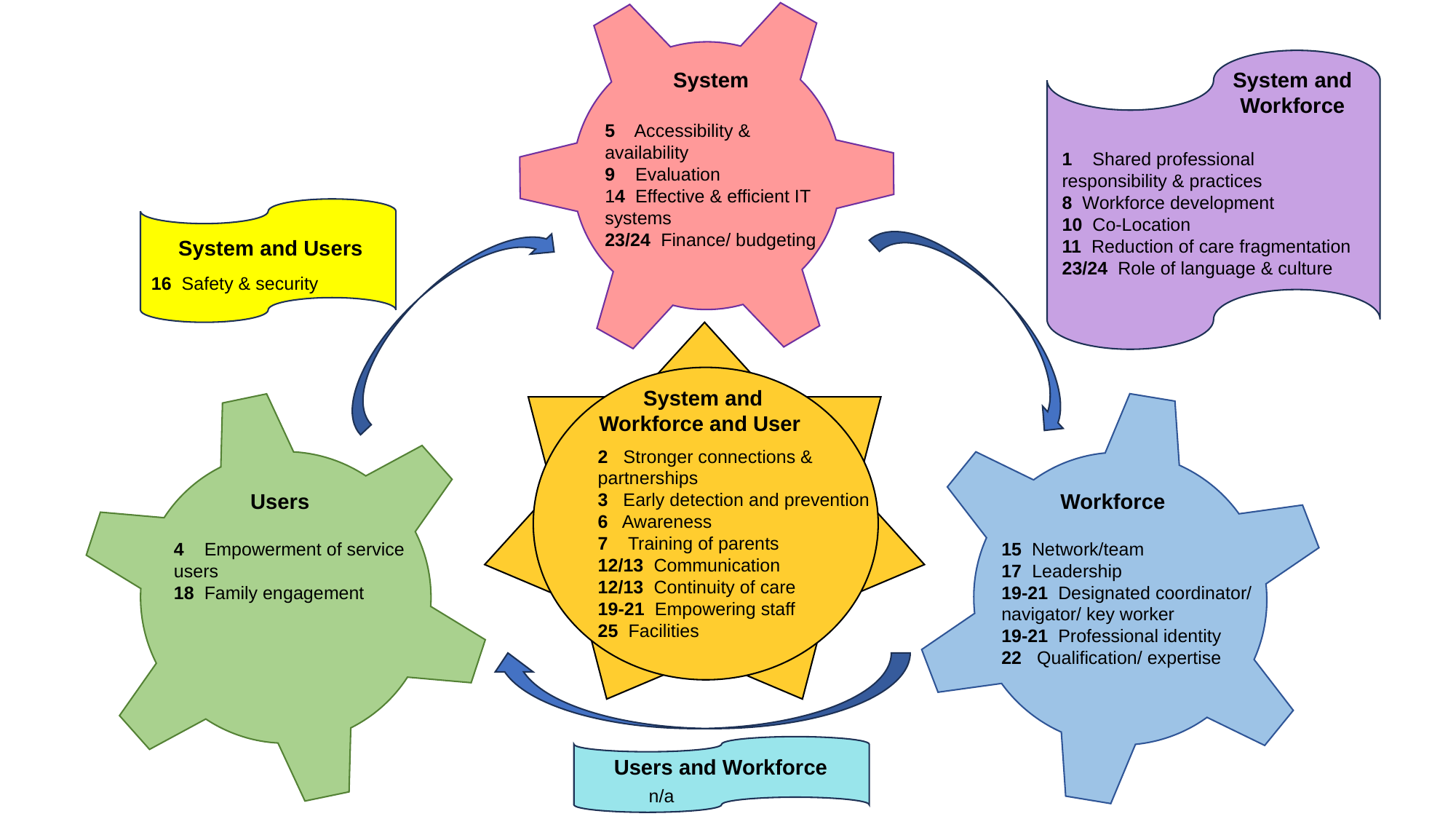

System
System and Workforce
5 Accessibility & availability
9 Evaluation
14 Effective & efficient IT systems
23/24 Finance/ budgeting
1 Shared professional responsibility & practices
8 Workforce development
10 Co-Location
11 Reduction of care fragmentation
23/24 Role of language & culture
System and Users
16 Safety & security
System and Workforce and User
2 Stronger connections &
partnerships
3 Early detection and prevention
6 Awareness
7 Training of parents
12/13 Communication
12/13 Continuity of care
19-21 Empowering staff
25 Facilities
Users
Workforce
4 Empowerment of service users
18 Family engagement
15 Network/team
17 Leadership
19-21 Designated coordinator/ navigator/ key worker
19-21 Professional identity
22 Qualification/ expertise
Users and Workforce
n/a

## Slide 3
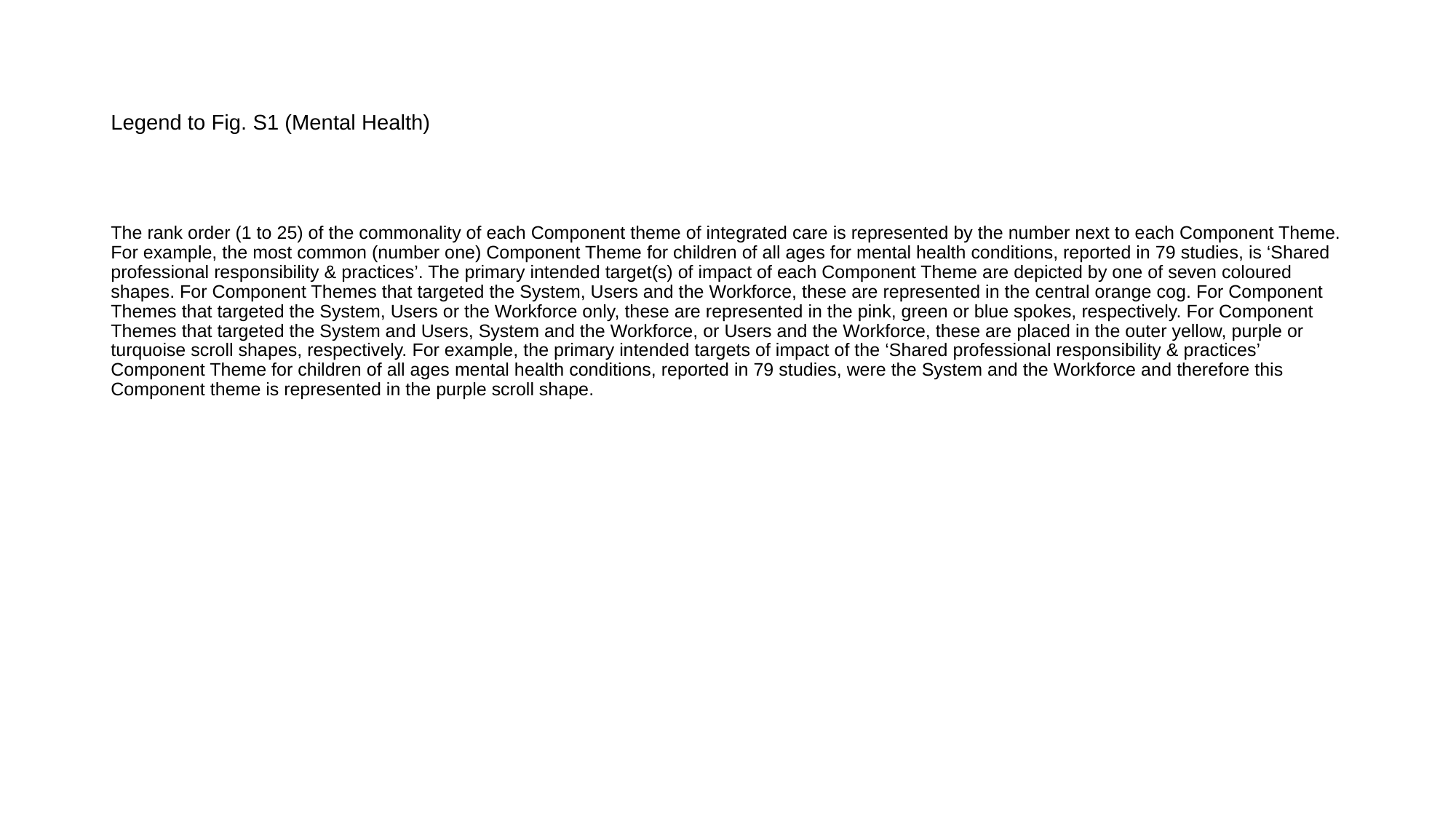

# Legend to Fig. S1 (Mental Health)
The rank order (1 to 25) of the commonality of each Component theme of integrated care is represented by the number next to each Component Theme. For example, the most common (number one) Component Theme for children of all ages for mental health conditions, reported in 79 studies, is ‘Shared professional responsibility & practices’. The primary intended target(s) of impact of each Component Theme are depicted by one of seven coloured shapes. For Component Themes that targeted the System, Users and the Workforce, these are represented in the central orange cog. For Component Themes that targeted the System, Users or the Workforce only, these are represented in the pink, green or blue spokes, respectively. For Component Themes that targeted the System and Users, System and the Workforce, or Users and the Workforce, these are placed in the outer yellow, purple or turquoise scroll shapes, respectively. For example, the primary intended targets of impact of the ‘Shared professional responsibility & practices’ Component Theme for children of all ages mental health conditions, reported in 79 studies, were the System and the Workforce and therefore this Component theme is represented in the purple scroll shape.

## Slide 4
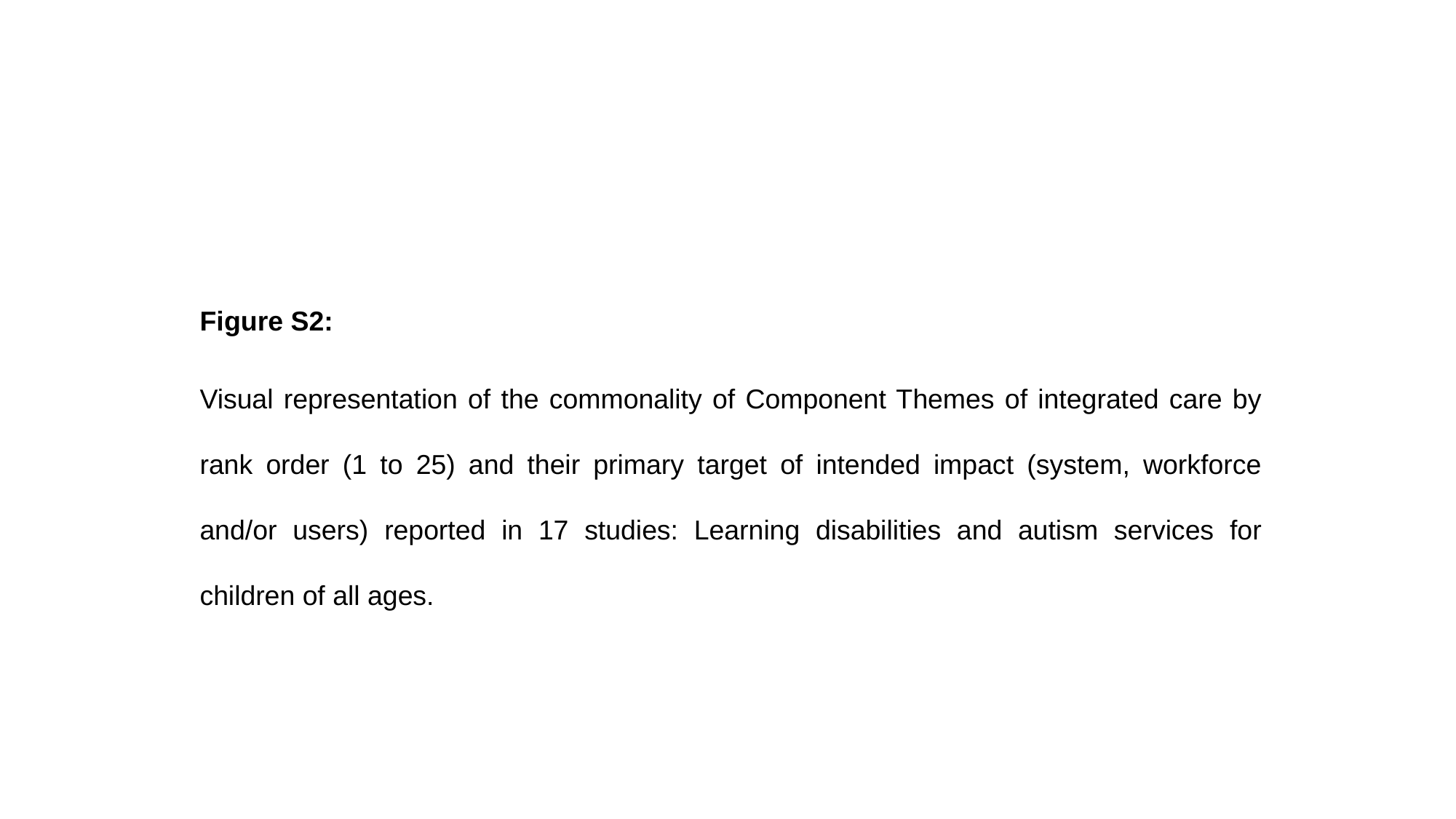

Figure S2:
Visual representation of the commonality of Component Themes of integrated care by rank order (1 to 25) and their primary target of intended impact (system, workforce and/or users) reported in 17 studies: Learning disabilities and autism services for children of all ages.

## Slide 5
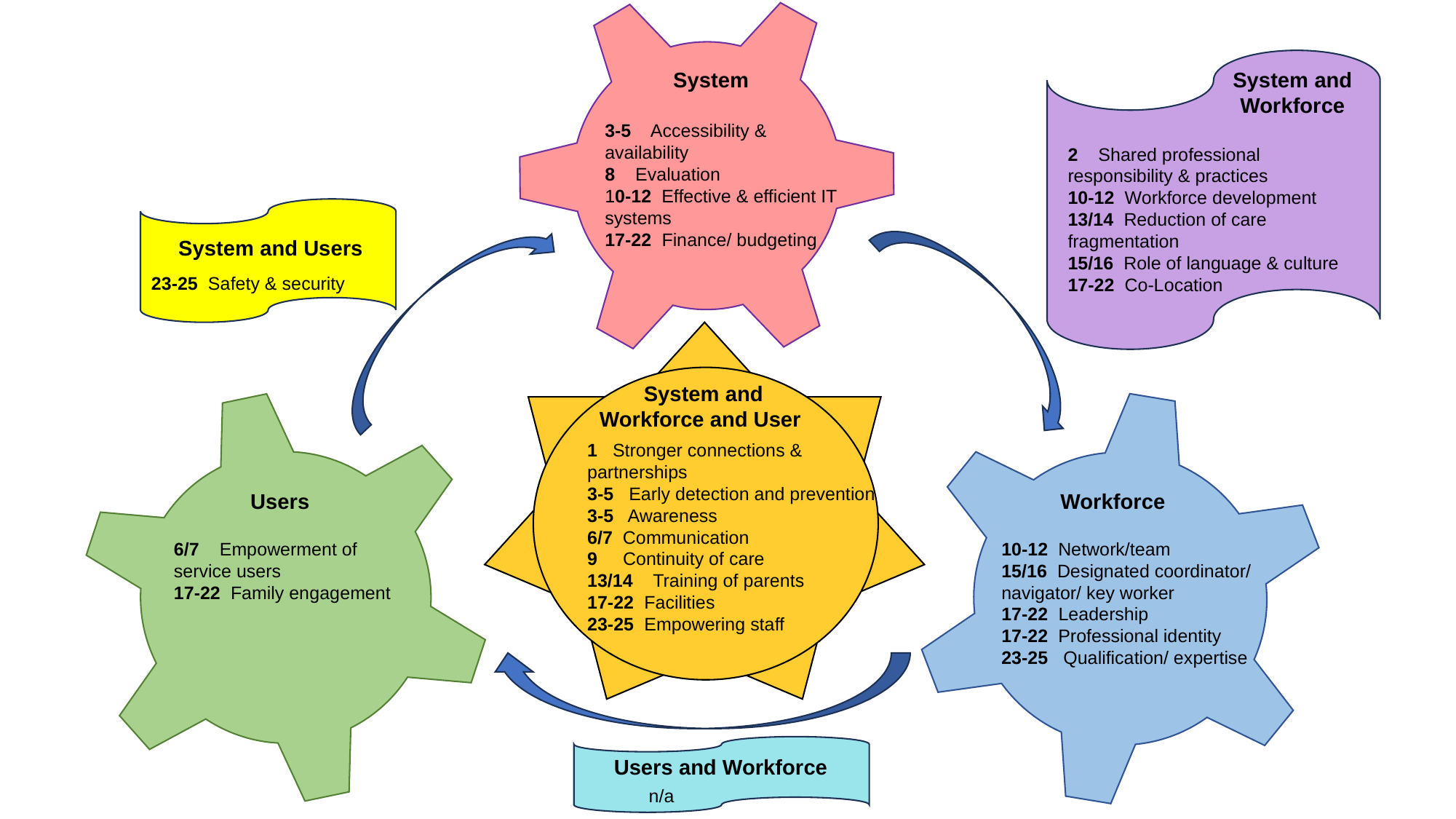

System
System and Workforce
3-5 Accessibility & availability
8 Evaluation
10-12 Effective & efficient IT systems
17-22 Finance/ budgeting
2 Shared professional responsibility & practices
10-12 Workforce development
13/14 Reduction of care fragmentation
15/16 Role of language & culture
17-22 Co-Location
System and Users
23-25 Safety & security
System and Workforce and User
1 Stronger connections &
partnerships
3-5 Early detection and prevention
3-5 Awareness
6/7 Communication
9 Continuity of care
13/14 Training of parents
17-22 Facilities
23-25 Empowering staff
Users
Workforce
6/7 Empowerment of service users
17-22 Family engagement
10-12 Network/team
15/16 Designated coordinator/ navigator/ key worker
17-22 Leadership
17-22 Professional identity
23-25 Qualification/ expertise
Users and Workforce
n/a

## Slide 6
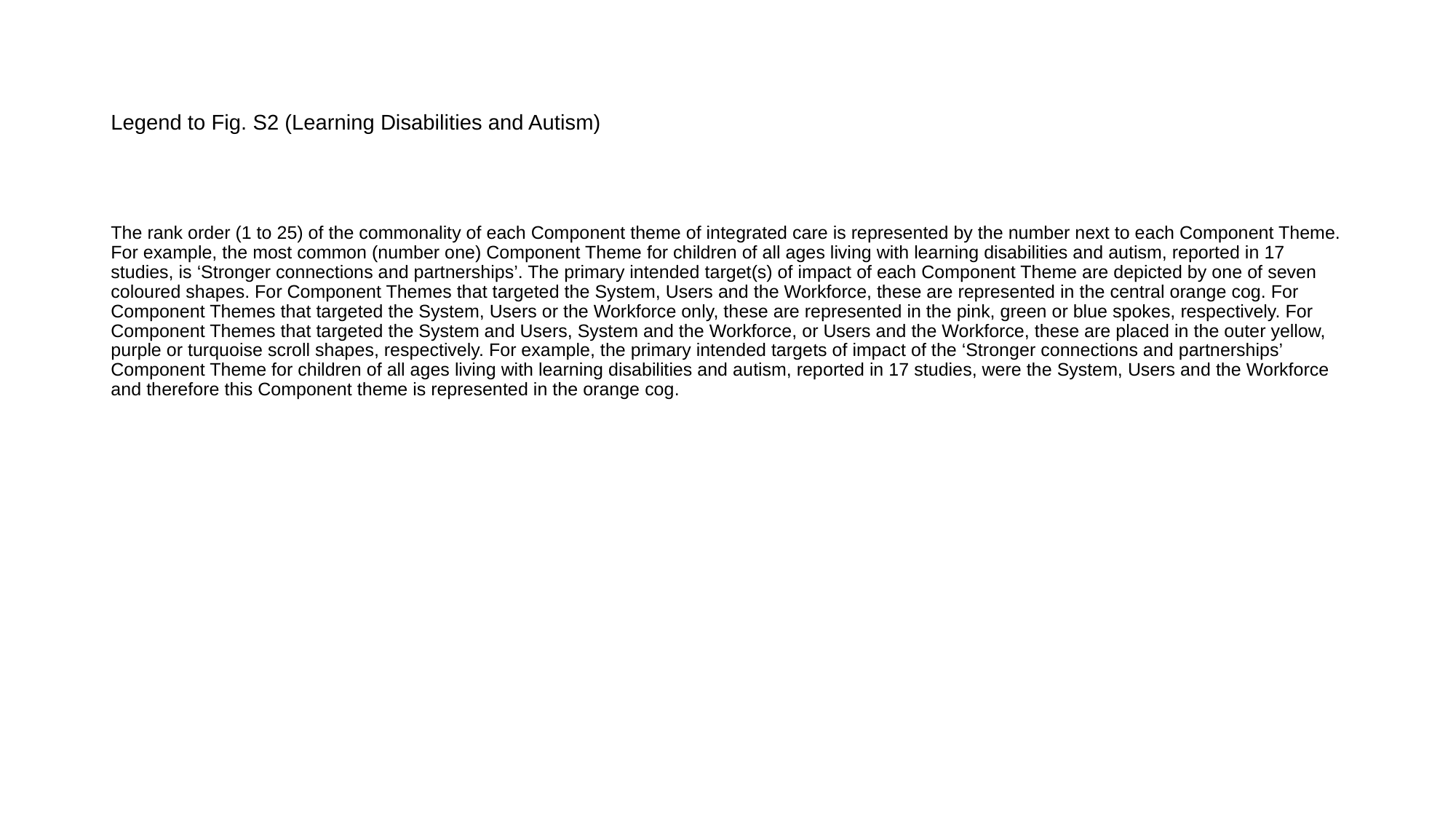

# Legend to Fig. S2 (Learning Disabilities and Autism)
The rank order (1 to 25) of the commonality of each Component theme of integrated care is represented by the number next to each Component Theme. For example, the most common (number one) Component Theme for children of all ages living with learning disabilities and autism, reported in 17 studies, is ‘Stronger connections and partnerships’. The primary intended target(s) of impact of each Component Theme are depicted by one of seven coloured shapes. For Component Themes that targeted the System, Users and the Workforce, these are represented in the central orange cog. For Component Themes that targeted the System, Users or the Workforce only, these are represented in the pink, green or blue spokes, respectively. For Component Themes that targeted the System and Users, System and the Workforce, or Users and the Workforce, these are placed in the outer yellow, purple or turquoise scroll shapes, respectively. For example, the primary intended targets of impact of the ‘Stronger connections and partnerships’ Component Theme for children of all ages living with learning disabilities and autism, reported in 17 studies, were the System, Users and the Workforce and therefore this Component theme is represented in the orange cog.

## Slide 7
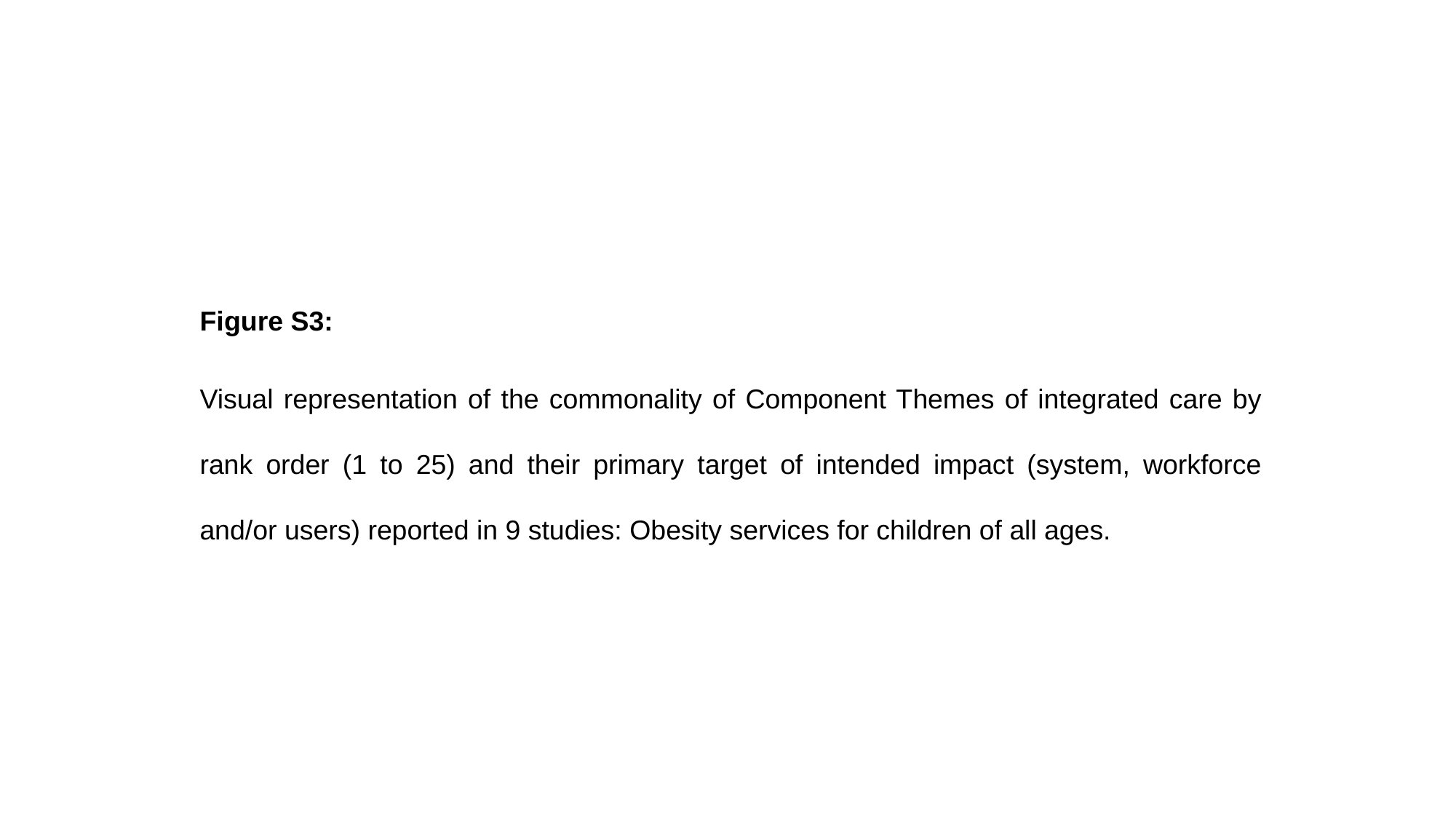

Figure S3:
Visual representation of the commonality of Component Themes of integrated care by rank order (1 to 25) and their primary target of intended impact (system, workforce and/or users) reported in 9 studies: Obesity services for children of all ages.

## Slide 8
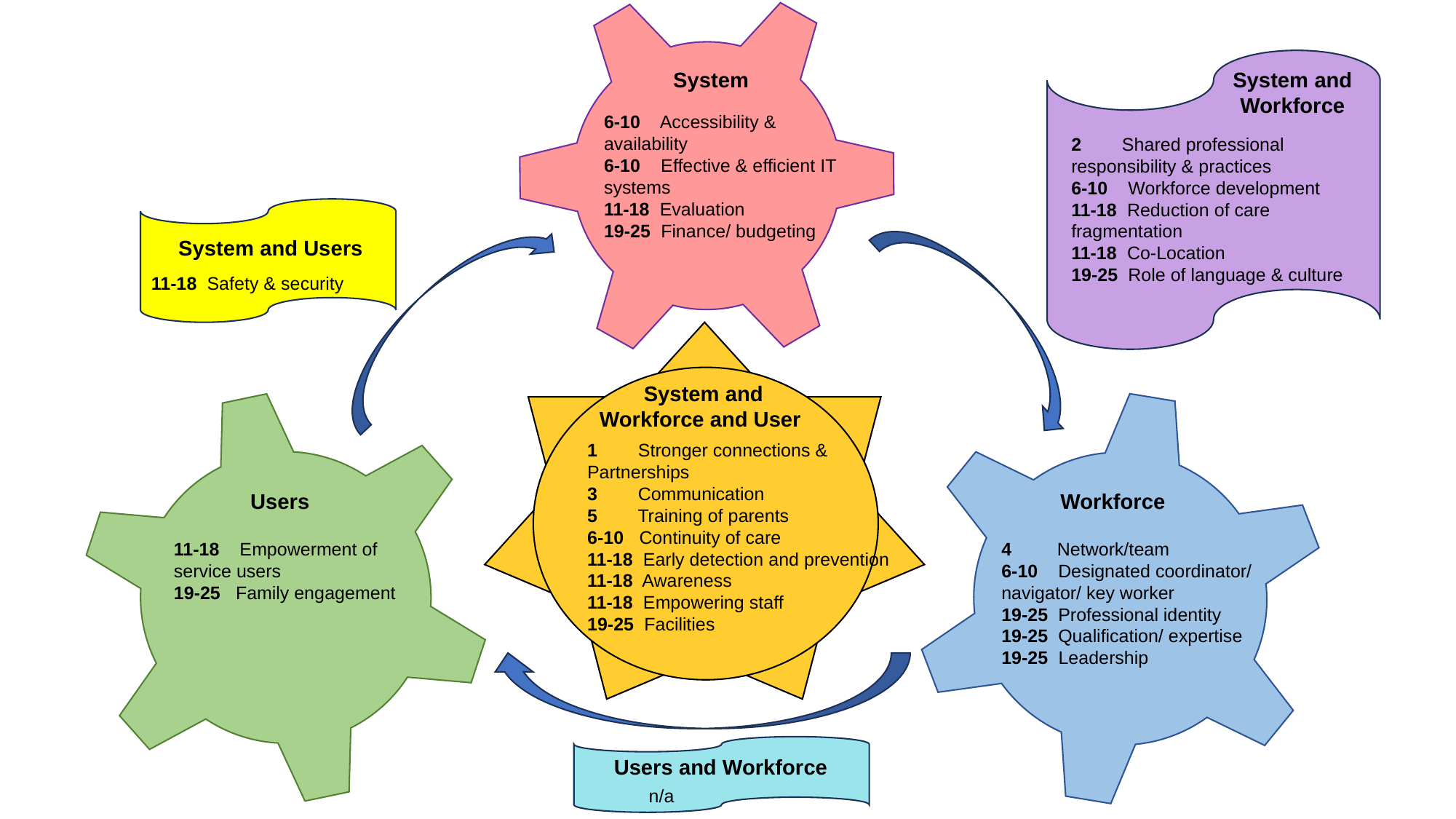

System
System and Workforce
6-10 Accessibility & availability
6-10 Effective & efficient IT systems
11-18 Evaluation
19-25 Finance/ budgeting
2 Shared professional responsibility & practices
6-10 Workforce development
11-18 Reduction of care fragmentation
11-18 Co-Location
19-25 Role of language & culture
System and Users
11-18 Safety & security
System and Workforce and User
1 Stronger connections &
Partnerships
3 Communication
5 Training of parents
6-10 Continuity of care
11-18 Early detection and prevention
11-18 Awareness
11-18 Empowering staff
19-25 Facilities
Users
Workforce
11-18 Empowerment of service users
19-25 Family engagement
4 Network/team
6-10 Designated coordinator/ navigator/ key worker
19-25 Professional identity
19-25 Qualification/ expertise
19-25 Leadership
Users and Workforce
n/a

## Slide 9
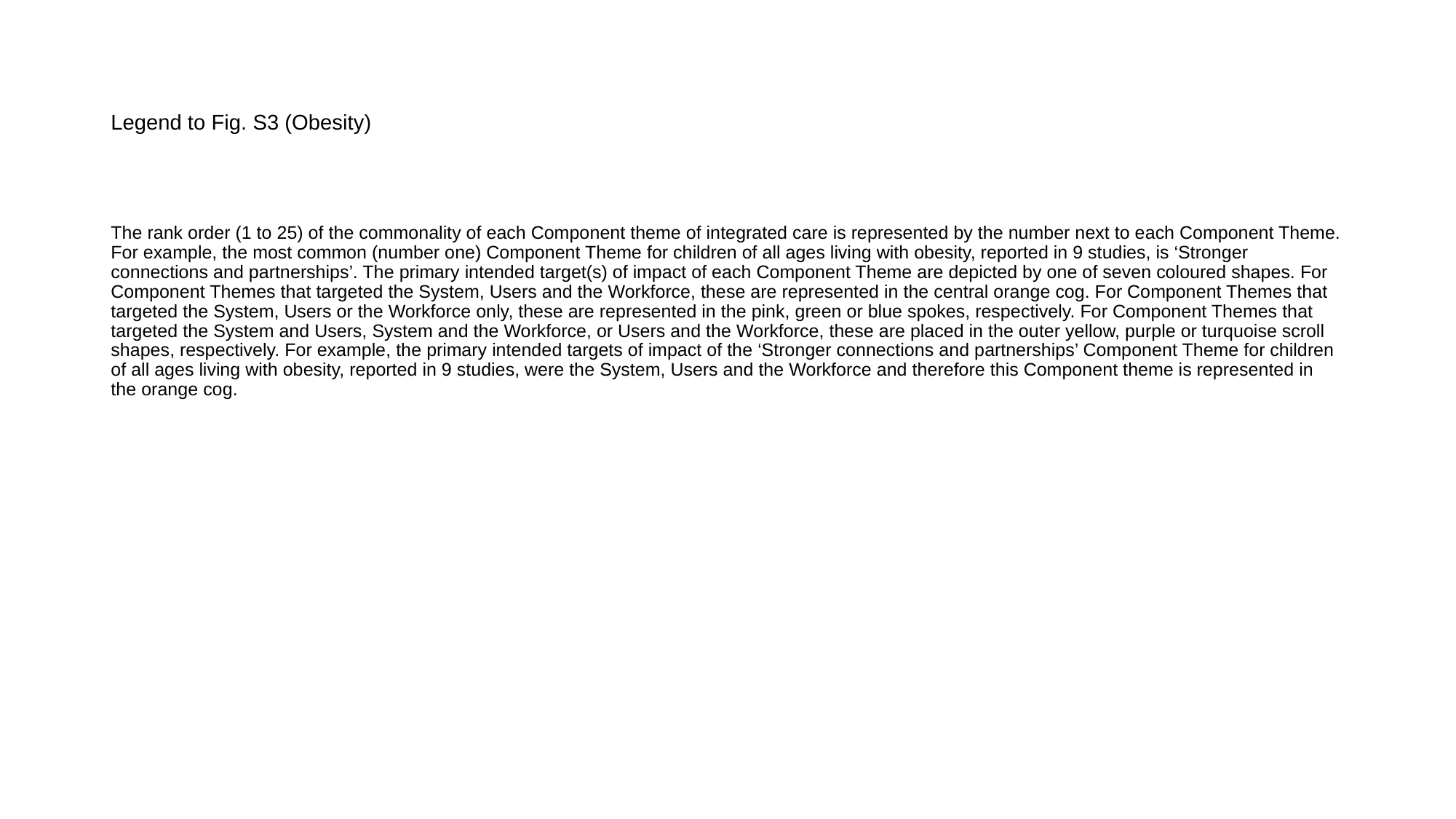

# Legend to Fig. S3 (Obesity)
The rank order (1 to 25) of the commonality of each Component theme of integrated care is represented by the number next to each Component Theme. For example, the most common (number one) Component Theme for children of all ages living with obesity, reported in 9 studies, is ‘Stronger connections and partnerships’. The primary intended target(s) of impact of each Component Theme are depicted by one of seven coloured shapes. For Component Themes that targeted the System, Users and the Workforce, these are represented in the central orange cog. For Component Themes that targeted the System, Users or the Workforce only, these are represented in the pink, green or blue spokes, respectively. For Component Themes that targeted the System and Users, System and the Workforce, or Users and the Workforce, these are placed in the outer yellow, purple or turquoise scroll shapes, respectively. For example, the primary intended targets of impact of the ‘Stronger connections and partnerships’ Component Theme for children of all ages living with obesity, reported in 9 studies, were the System, Users and the Workforce and therefore this Component theme is represented in the orange cog.

## Slide 10
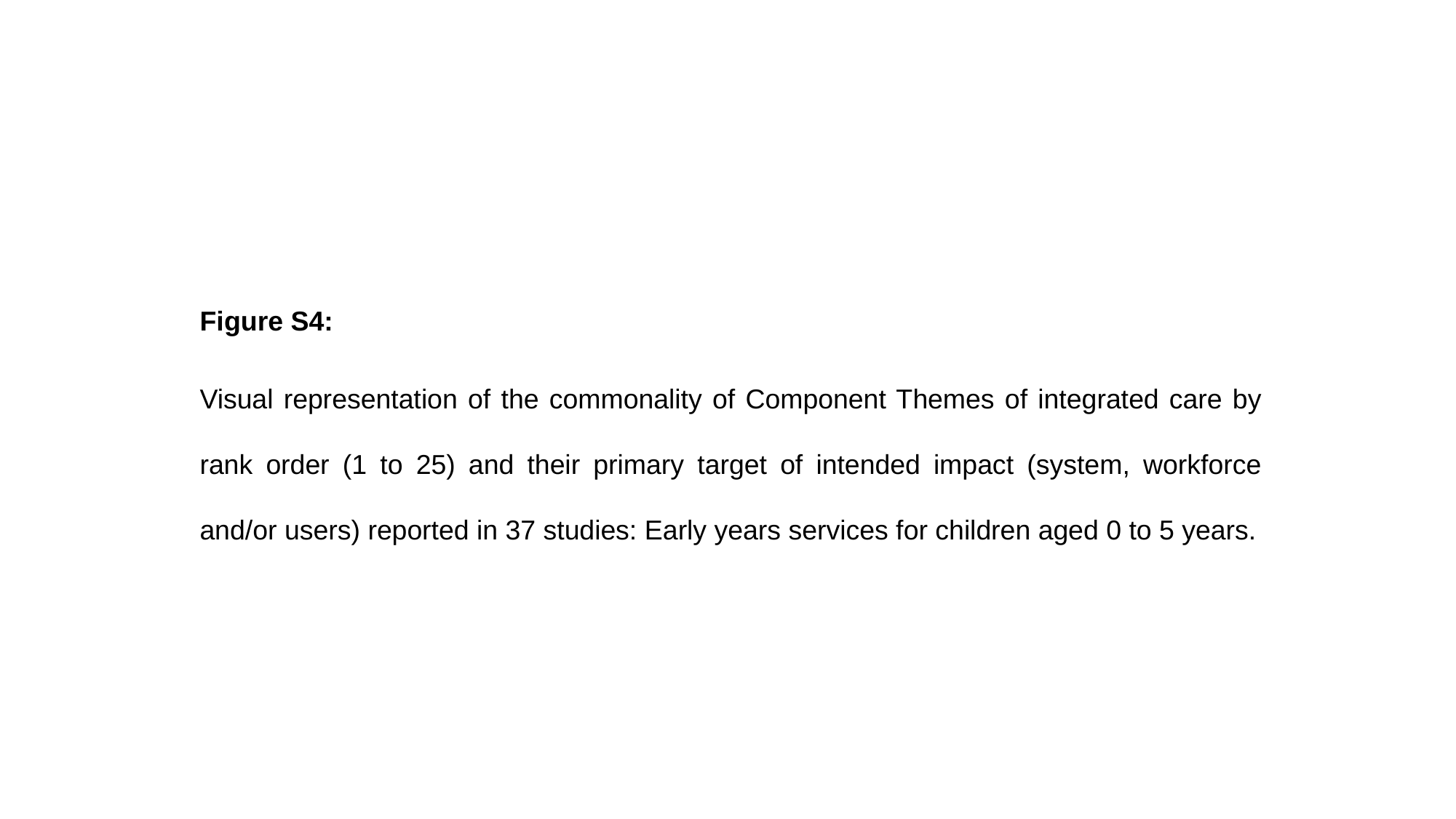

Figure S4:
Visual representation of the commonality of Component Themes of integrated care by rank order (1 to 25) and their primary target of intended impact (system, workforce and/or users) reported in 37 studies: Early years services for children aged 0 to 5 years.

## Slide 11
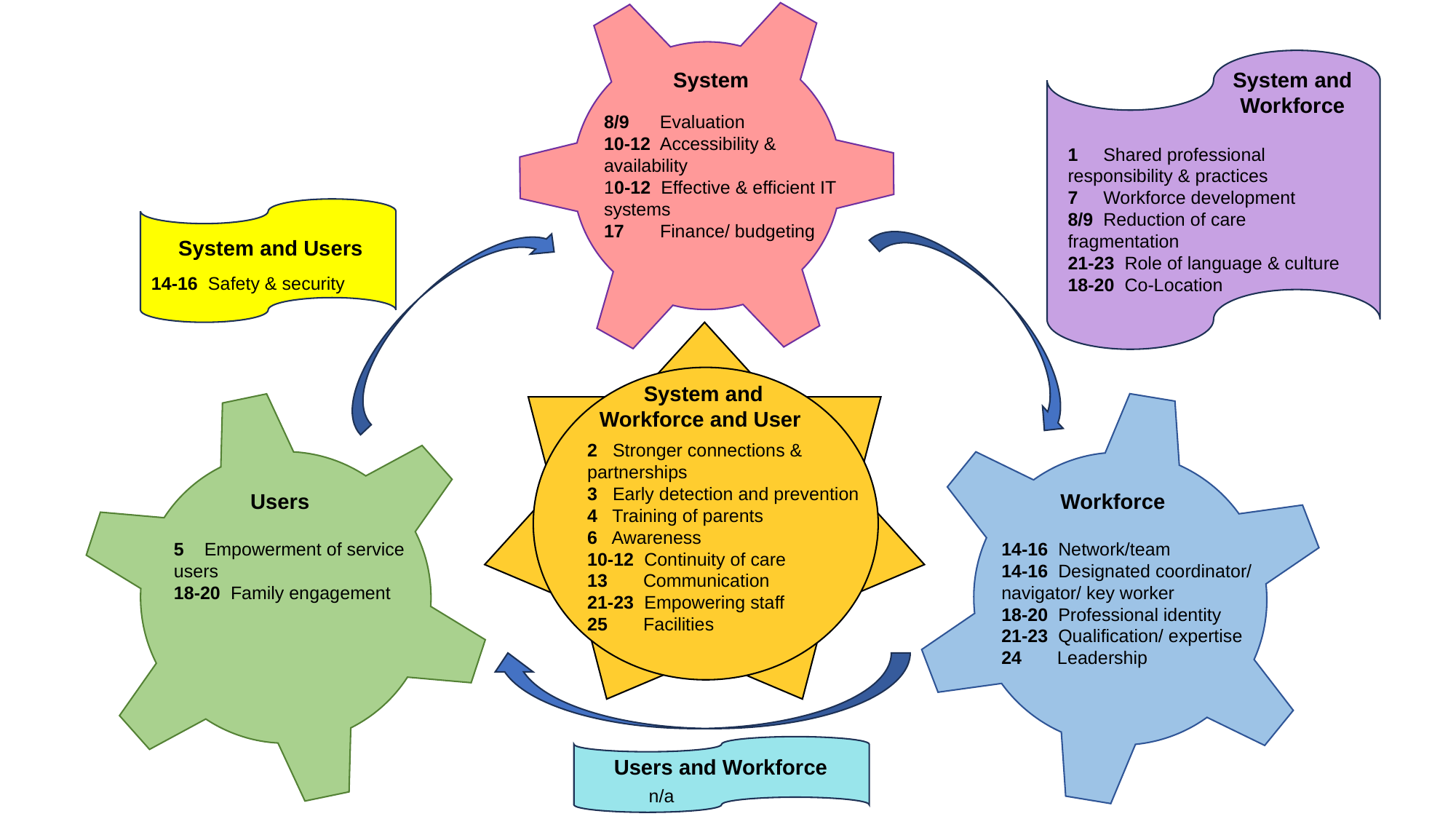

System
System and Workforce
8/9 Evaluation
10-12 Accessibility & availability
10-12 Effective & efficient IT systems
17 Finance/ budgeting
1 Shared professional responsibility & practices
7 Workforce development
8/9 Reduction of care fragmentation
21-23 Role of language & culture
18-20 Co-Location
System and Users
14-16 Safety & security
System and Workforce and User
2 Stronger connections &
partnerships
3 Early detection and prevention
4 Training of parents
6 Awareness
10-12 Continuity of care
13 Communication
21-23 Empowering staff
25 Facilities
Users
Workforce
5 Empowerment of service users
18-20 Family engagement
14-16 Network/team
14-16 Designated coordinator/ navigator/ key worker
18-20 Professional identity
21-23 Qualification/ expertise
24 Leadership
Users and Workforce
n/a

## Slide 12
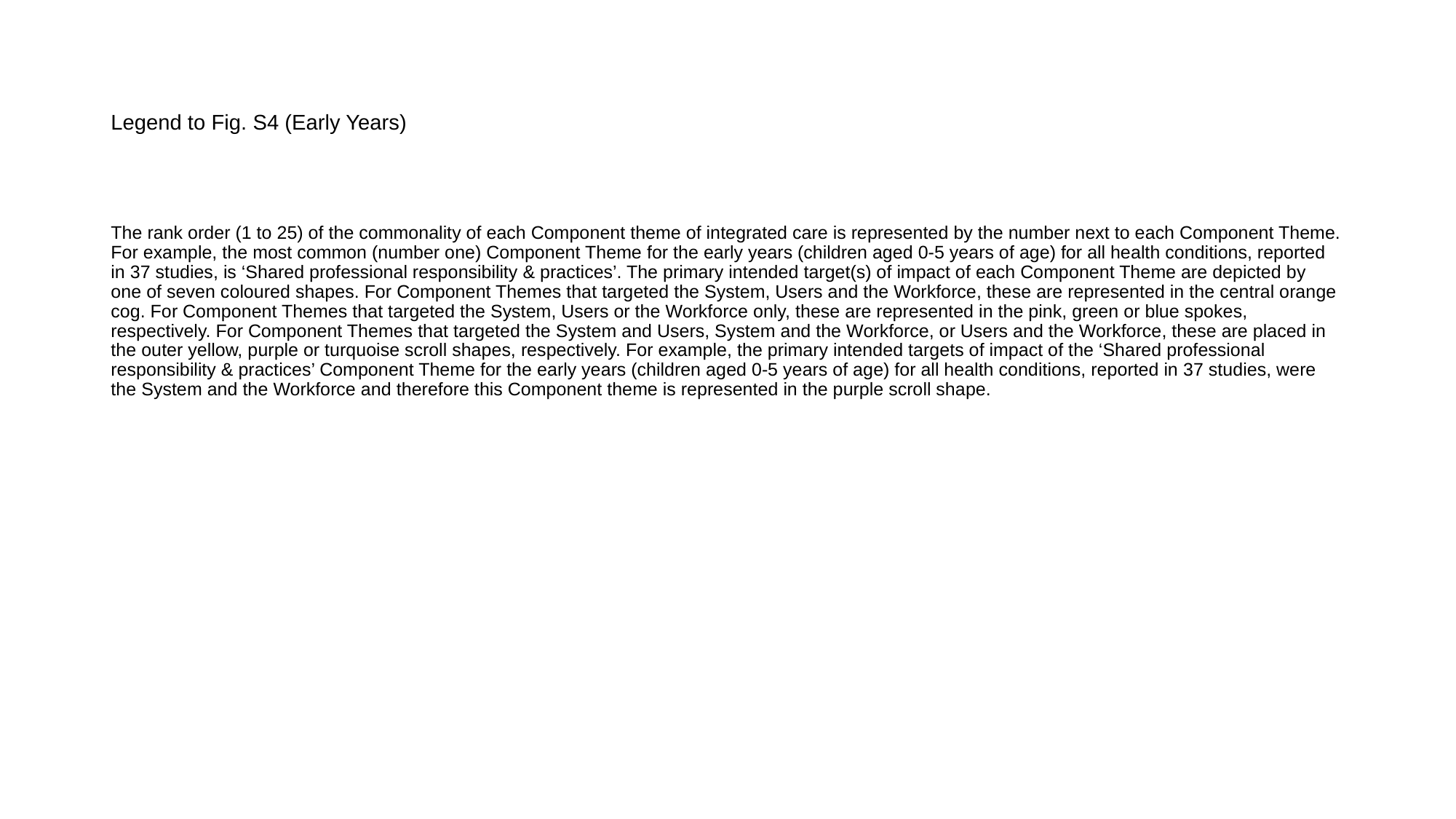

# Legend to Fig. S4 (Early Years)
The rank order (1 to 25) of the commonality of each Component theme of integrated care is represented by the number next to each Component Theme. For example, the most common (number one) Component Theme for the early years (children aged 0-5 years of age) for all health conditions, reported in 37 studies, is ‘Shared professional responsibility & practices’. The primary intended target(s) of impact of each Component Theme are depicted by one of seven coloured shapes. For Component Themes that targeted the System, Users and the Workforce, these are represented in the central orange cog. For Component Themes that targeted the System, Users or the Workforce only, these are represented in the pink, green or blue spokes, respectively. For Component Themes that targeted the System and Users, System and the Workforce, or Users and the Workforce, these are placed in the outer yellow, purple or turquoise scroll shapes, respectively. For example, the primary intended targets of impact of the ‘Shared professional responsibility & practices’ Component Theme for the early years (children aged 0-5 years of age) for all health conditions, reported in 37 studies, were the System and the Workforce and therefore this Component theme is represented in the purple scroll shape.
