# Supplementary material for: Commonality of 25 component themes of integrated care for children: rapid review of 170 models
Source: BMC Health Serv Res. 2025 Oct 8;25:1332. doi: 10.1186/s12913-025-13345-w (PMC12505997; doi:10.1186/s12913-025-13345-w)
Supplement: Supplementary file 2 — Supplementary Material 2. [file 12913_2025_13345_MOESM2_ESM.docx]

**Supplementary File**

**Table S1: Search words used to identify studies for this review.**

| Concept 1  **Integrated care** | Concept 2  **Child population** | Concept 3  **Components** |
| --- | --- | --- |
| "collaborative care" OR "cooperative care" OR "coordinated care" OR "coordination of care" OR "cross sectoral care" OR "functional integration" OR "horizontal integration" OR "integrated care" OR "integrated service network*" OR "integration of care" OR "intersectoral care" OR "linked care" OR "seamless care" OR "service network*" OR "shared care" OR "vertical integration" OR "continuity of care" OR "care continuity" | child* OR "young people" OR youth* OR pediatric* OR paediatric* | Component* OR feature* OR “building block*” OR strateg* OR mechanism* OR approach* OR model* OR implement* OR framework* OR Function* OR pathway* OR constituent* |

**Table S2: Description of the 170 included studies: their coded components of integration, setting, target population, target health condition and country of location.**

| **Author, Year (and reference number)** | **Description of components identified by the study and the Component Theme code number it was coded to, and the target(s) of impact of this component (S, U and/or W).** | **Setting** | **Main Target Population, including Early Years** | **Target Health condition(s) as reported by authors** | **Main Health Condition, including Mental Health, Learning disabilities and Autism   (LD& Autism) and Obesity** | **Country** |
| --- | --- | --- | --- | --- | --- | --- |
| Ablard 2020  (31) | Service users empowerment (3) (S; U);  Individual care record system (5) (S);  Single-point of access (5) (S);  ‘Joined-up’ UEC system (12) (S) | Primary Care | General; Early years focus | general | general | UK |
| Acri 2016  (32) | Clinical sustainability of the model (5) (S);  Training of staff (7) (W);  “Bottom up” approach (12) (S);  Linking integrated health model to pediatric centre (decreasing fragmentation) embedded a screener and care coordinator into the practice to reduce potential time-related issues related to assessment and care coordination (12) (S; W);  Screening instrument (12) (S) | Primary care; Mental health service (MH) | Vulnerable Groups | Disruptive behaviour disorder, Mental Health | Mental Health | US |
| Altman 2018  (33) | Care coordination between patients and family-centred team (1) (U; W).  Shared decision making and responsibility among providers (1) (W).  Support through networking, education and partnership empowerment (1) (S; U; W);  Active care (1, 18) (S; W);  Establishing communication channels (11) (U; W);  Coordination across multiple systems of care (12) (S)  Leadership (community lead, family lead, clinician lead) (17) (W) | Primary care; Hospital care | General | Long-term complex medical conditions | Long-term complex medical conditions | Australia |
| Bailey-Davis 2018  (34) | Shared care records (2) (S);  Family education (5) (U);  Coordination of preventative care with evidence-based strategies (4) (S); | Public Health: Special Supplemental Program for Women, Infants and Children (WIC) | Early years | Obesity | Obesity | US |
| Bali 2016  (35) | Shared electronic record (1) (S);  Individualised care plans (5) (S);  A named paediatrician with appropriate expertise as a central person (leader) (5) (W);  Multi-sectoral economic evaluation (whole system strategy) (9) (S)  Co-created CYP networks (Tailored care) (10) (U);  Enhanced professional networks (24) (W); | Primary, Secondary, Tertiary and Quaternary healthcare.  Multi-sectoral | General | Epilepsy | Epilepsy, disability | UK |
| Beers 2017  (36) | A systems-based and collaborative approach (2) (S);  The promotion of routine annual mental health screening in primary care (4) (W);  Meaningful involvement of parents and families (5) (W);  Involvement of cross-sector and multidisciplinary teams (2) (W);  An investment of resources (7) (S);  A culture of evaluation and improvement (7) (S);  Clearly defined goals (24) (W) | MH; Primary care | General, CYP with MH problems | MH, developmental/ behavioural/ mental health | Mental Health | US |
| Berntsen 2018  (37) | Goal dictates the forming of a multidisciplinary team needed to assess the patient’s health issues (2) (W);  Patient involvement and self-management (3) (U);  Specify and focus on the overarching goal identified by a patient (4) (U);  The treatment plan should support health literacy (4) (U);  Goal evaluation (patient-driven evaluation of goal-attainment) (9) (U);  Timeline map: “individualized Patient Pathway” (12) (U);  The team is responsible for care delivery (15) (W); | General practice; Nursing services; Hospital | CYP with long-term conditions | Multiple long-term conditions and complex healthcare needs; MH | Long-term complex medical conditions; Mental health | Norway |
| Bethell 2022  (38) | Collaboration between professionals and parents (5) (S; W);  Training of staff (7) (W);  Teams and teamwork (15) (W);  Payment and performance measurement based on child health outcomes (20) (S) | Primary care | General; Early years focus | General | General | US |
| Boege 2018  (39) | Early diagnostics (school-based assessment and school-based intervention) (4) (W);  Early screening (4) (U);  Therapists to receive e-learning training (7) (W); | School; Mental health care services; Youth welfare services | School years | Psychiatric disorders; MH | Mental health | Germany |
| Breen 2018  (40) | Care coordination between patients and family-centred team (1) (U; W);  Clear division of responsibilities (1) (W);  Support through networking, education and partnership (1; 16) (W);  Consistent communication (2) (W);  Leadership (community lead, family lead, clinician lead) (17) (W) | tertiary hospital, primary care | CYP with long-term conditions | Medical complexity, long-term conditions | Long-term complex medical conditions | Australia |
| Brenner 2018a  (41) | Coordinated pathways to specialist care and legal and governance structures (1) (S);  Quality of care (2) (S);  Integrated services (2) (S);  Safeguarding (4) (U);  Nurse preparation (7) (W);  Standardising specialist training (7) (W);  Accessible care: equitable availability of consistently high-quality, prompt, and accessible services (8) (S);  Geographical variation (8) (S);  Referral-discharge interface (examination of structures and processes) (10; 12) (S);  Social care interface (10) (S);  Accessible care: equitable availability of consistently high-quality, prompt, and accessible services (8) (S);  Geographical variation (8) (S);  Co-creation of care with parents (18) (U); | Acute–community care | CYP with complex care needs | Complex care needs | Long-term complex medical conditions | European Union (EU) and European Economic Area (EEA) |
| Brenner 2018b  (42) | Family–professional partnership (2) (U; W);  Community-based services and support (8) (S; U);  Quality assurance: service providers and parents or guardians (9) (S; U; W) | Acute–community care | CYP with complex care needs | Complex care needs | Long-term complex medical conditions | European Union (EU) and European Economic Area (EEA) |
| Brenner 2020  (43) | Coordinated pathway to specialist care legal and governance structures (1) (S);  Family preparedness for transitioning to home (13) (U) | Community and acute care | CYP with complex care needs; CYP with long-term conditions | Children assisted with long-term home ventilation, complex care needs | Long-term complex medical conditions | European Union (EU) and European Economic Area (EEA) |
| Brenner 2021  (44) | Ongoing hospital/community interface (2) (S);  Effective health and multi-agency agreements and funding arrangements (2; 20) (S);  Accessible accommodation, equipment and transport (8) (S);  Appropriate and effective care package (12) (W);  Effective discharge planning procedures (13) (W);  Key worker delivered individually tailored family support and education (16) (W);  Robust clinical governance, quality and safety policies (19) (S) | Community health units | CYP with complex care needs | Complex healthcare needs | Long-term complex medical conditions | Ireland |
| Briggs 2016  (45) | Co-location in community health centres (1; 8; 14) (S);  Flexible model (3) (S);  Early identification: proactive and preventive assessment of prenatal problems (4) (S; W);  Additional behavioural consultations (4) (W);  Screening and early short-term treatment (4) (U; W);  Early response based on screening (4) (U; W);  Warm hand-off (5) (W);  Training and on-going supervision (7) (W) | Primary care | Early years | Behavioural health care, MH | long-term complex medical conditions; Mental health | US |
| Bruni 2021  (46) | Care coordination between patients and family-centred team (1) (U; W);  Co-location of multidisciplinary and/or interagency staff (1, 14) (S);  Parents involved in child’s treatment (psycholeducation) (5) (U);  Meeting the needs of parents (6) (U; W);  Evidence –based behavioural treatment (7) (S; W);  Confluence of physical and mental health (14) (S);  Privacy to parents and families (19) (S; U) | Primary care | CYP with MH problems | MH | Mental health | US |
| Bui 2019  (47) | Coordinated care (1) (S; W);  Addressing the social determinants of health (6) (S);  Value-based payment models (20) (S) | Prenatal and natal care | Early years | Early years | General | US |
| Burkhart 2020  (48) | Co-location (e.g., a separate behavioural health provider receives a facilitated referral to treat a patient from a partnered primary care practice down the hall) (1; 14) (S);  Person-centred and holistic (5) (U; W);  The formation of partnerships between primary care clinicians and child and adolescent psychiatrists (13) (W);  Team-based care (15) (W) | Primary care | CYP with MH problems | Psychiatric disorders (e.g., anxiety, depression, ADHD) MH | Mental health | US |
| Cady 2020  (49) | Partnerships with families (1; 2) (U; W);  Family-centred care coordination (3; 5) (U; W);  Effective information exchange between primary care, speciality care, and patients/families (10; 11) (U; W);  Single point of contact that communicates and coordinates with patients, families, clinicians, and services (16) (U) | Emergency department | CYP with complex care needs; CYP with long-term conditions | Multiple chronic conditions, medical complexity | Long-term complex medical conditions | US |
| Casher 2021  (50) | Integrated psychology trainees in a safety net hospital's pediatric ED and urgent care (4; 13) (W) | Emergency Department and Urgent Care | CYP with MH problems | MH | Mental Health | US |
| Chasson 2021  (51) | Information exchange and individualised care plans (decentralised decision-making) (1) (S);  Access to and input from specialised care (2) (S);  Building families agency (3) (U) | Autism‑Specific Medicaid Waive | CYP with ASD | ASD (Autistic Spectrum Disorder) | LD& Autism | US |
| Choi 2018  (52) | Patient-centred care (3; 5) (U);  Qualification of staff (22) (W) | Early Head Start | Vulnerable Groups; Early years | General | General | US |
| Christiansen 2019  (53) | Addressing the child’s and family’s needs (1; 3) (U; W);  Child-centred approach (3) (U; W);  Capturing the “child’s voice”(3) (U);  Co-development and co-design (5) (U; W) | Mental health care units | CYP with MH problems | Children of parents with a mental illness (COPMI), MH | Mental Health | Austria |
| Christison 2018  (54) | Sharing the care of the patient and patient/family inclusion in care planning and delivery (1) (U);  Coordination of providers with defined roles (1) (W);  PCP considering consultant recommendations and implementing them when appropriate (2) (W);  Self-management support (5) (U);  Access to primary and tertiary care (8) (U);  Facilitating communication among all healthcare- and community-based treatment team members (11) (W);  Communication between families (11) (U);  Communication between PCPs and specialists (11) (W);  Reduction of fragmentation for children through assisting child/family with communicating clinical issues (12) (U; W);  Supporting and revising care plans (longitudinal plans toward transition from specialty back to primary care and pediatric to adult care) (13) (W);  Ensuring successful linkages with community-based resources (13) (W) | Tertiary care; primary care | CYP with Obesity | Obesity | Obesity | US |
| Cicutto 2018  (55) | Formation of partnerships between schools, families, and community health-care providers (2; 13) (S);  Evidenced-based care (9) (S);  Continuity of care (12) (S);  The need for ‘champions’ (23) (W) | School and community health-care providers | CYP with Asthma | Asthma | Asthma | US |
| Conners 2017  (56) | Safe transitions of care (5) (U);  Continuous improvement (5) (S);  Scope of the familiar emergency response role (5; 23) (W);  Pediatric-trained staff (7) (W);  Telemedicine (10) (S);  School-based illness clinics (12) (S);  A defined scope of services (24) (S) | Non-emergency Acute Care | General; Early years | General | General | US |
| Connor 2018  (57) | Patient education and advice (3) (U);  Coordination included education for patients and guidance on monitoring symptoms, scheduling a clinic visit (4) (U);  Logistical medical support (8) (S);  Coordination is driven by patient needs for the interaction on clinical management issues such as questions and concerns related to symptoms and medications (12) (S; U) | Paediatric Cardiology Ambulatory Setting | CYP with Cardiology | Cardiology | Cardiology | US |
| Contreras 2018  (58) | Collaboration (e.g., the primary care practice has its own integrated behavioural health provider who collaborates with practice staff to serve all patients) (1) (S);  Active and on-going partnerships (1) (W);  Phone screening (10) (S);  A multi-stepped intervention that addressed the major gaps in the referral system (12) (S);  Intake visit (13) (W) | Community-based mental health settings | CYP with MH problems | MH | Mental Health | US |
| Cooper 2022  (59) | Better access to care (8) (S; U);  Continuity of care (13) (S) | Primary care | CYP with Asthma | Asthma | Asthma | Canada |
| Courtney 2020  (60) | Multiple stakeholder involvement (1) (W);  Clinicians and administrators involved as co-developers (1) (W);  Contextualising materials to youth culture (3) (U);  Iterative and reflective development and implementation cycles (6) (S; W);  Involving the voices of young people (18) (U);  Youth partners engaged to bring “patient” voice (18) (U) | Centre for Addiction and Mental Health (CAMH) | CYP with MH problems | Major depressive disorder; MH | Mental health | Canada |
| Crowe 2017  (61) | Providing a single point of contact (5) (S);  “Step-down care” (12) (S; U);  Additional training and support to non-English speaking families (24) (U) | community, primary, secondary and tertiary care | Early years | Congenital heart disease (CHD) | Cardiology | UK |
| Dale 2021  (62) | Interprofessional communication (i.e. communicating with patients, families, communities, and professionals in health and other fields in a responsive and responsible manner that supports a team approach) (1; 11) (U; W);  Collaboration: values and ethics (i.e. working with individuals of other professions to maintain a climate of mutual respect and shared values) (2) (S);  Roles and responsibilities (i.e., using the knowledge of one’s own role and those of other professions to appropriately assess and address healthcare needs) (7; 21) (S; W);  Teams and teamwork (i.e. applying relationship-building values and the principles of team dynamics to perform effectively in different team roles (15) (W) | School-Based Settings | School years | General health | General | US |
| Davies 2017  (63) | Early identification of severe injury (early identification of severe injury) (4) (S);  Provision of close supervision of family (4) (S; U) | Non-major trauma centres | Vulnerable Groups | Major trauma from suspected child abuse | Mental health | UK |
| Dayton 2016  (64) | Influence recruitment (opt-out and opt-in recruitment for participants) (3) (W);  Re-traumatizing family (5) (U; W);  Guidelines regarding confidential information (7; 19) (S);  Leadership buy-in (17) (W);  Involving families (18) (U);  Role clarity (23) (W) | Primary care | CYP with MH problems | MH | Mental Health | US |
| De Laat 2022  (65) | personal approach and broad perspective to each family (avoiding 'one size fits all' approach) (3) (S; U); specialist training for a nurse as a coordinator (7) (W); nurse as a coordinated professional to provide stepped and matched care (16) (S; W) | Primary Care | Children with overweight or obesity, children ≥12 | Overweight or obesity | Obesity | Netherlands |
| de Voursney and Huang 2016  (66) | Decision support (2) (W);  Community resources and policies (2) (S);  Self-management support (5) (U);  A specific role of a psychologist (7) (W);  The CCM delivery system design (The CCM model urges practices to define roles for members of the care team with doctors and other highly trained staff addressing acute problems, and training other team members who do more patient engagement and routine work) (7; 23) (S);  Clinical information systems (10) (S);  Primary care case management (in which primary care providers receive an enhanced payment to coordinate services, and integrated managed care) (12; 20) (W);  Health care organization (12) (W);  Coordinated services by a team of professionals (family partners and peer supports can also be an integral part of the care team) (15) (U; W) | Behavioural health services for children and youth | CYP with MH problems | Mental health disorders | Mental Health | US |
| Dela Merced 2022  (67) | Care coordination between patients and family-centred team (1) (W);  Evaluation (audit of relevant outcomes) (9) (S);  Integration of services/reducing care fragmentation (active care coordination and management) (12) (S) | Preoperative and postoperative care | CYP with pre and post operative care | Idiopathic scoliosis | Idiopathic scoliosis | US |
| Dillon-Naftolin 2017  (68) | Using a team approach that prioritizes shared goals (1) (S);  Mutual trust (1) (W);  Effective communication (1; 11) (S; W);  Clear division of responsibilities (1) (S; W);  Creating partnerships between primary care providers (PCPs) and mental health specialists (1) (W);  Stepped-care treatment interventions (direct consultation with a child and adolescent psychiatrist) (2) (U);  Safe and nurturing environment for families (3) (U);  Early screening (4) (U);  Coordination across multiple systems of care (12) (W);  Centred on working with families around their needs (12) (U);  Measurement-based treatment to target (12) (S);  Ongoing follow-up (13) (U; W) | Primary care | CYP with MH problems, Early years focus | MH | Mental health | US |
| Doucet 2019  (69) | Matching unmet needs to appropriate resources (1) (S);  Navigation of system (for patients) (3) (U);  Proactive guidance and support (3) (U; W);  Single point of contact (5) (W);  Training of lay public and peers (5) (U);  Capacity building (5) (S);  Improving access (8) (S; U);  Decreasing fragmentation (12) (S, U);  Promoting/facilitating integration of care (12) (S) | All | General | General | General | Canada |
| Eastwood 2019  (70) | Place-based neighbourhood initiatives (2) (S);  Collaborative planning (2) (W);  Evidence-informed interventions (6; 7; 9) (S);  Improving the flow of information between hospitals, specialists, community and primary care providers (10) (S);  Interagency system change (12) (S);  Developing new ways of working (21) (S) | Hospital and primary care services | Vulnerable Groups | Complex health and social care needs | Long-term complex medical conditions | Australia |
| Eastwood 2020a  (71) | Strengths-based, whole-of-family-involvement (1) (U);  High levels of trust between parties (2) (U; W);  Programme Flexibility (3) (S);  Increased interaction (“crossing of paths”) (3) (U);  Co-location (Located in the same physical space) (14) (S);  Building connections (15) (U; W); | Primary care, social services and hospitals, and community services | Vulnerable Groups; CYP with complex care needs | Complex health and social care needs | Long-term complex medical conditions | Australia |
| Eastwood 2020b  (72) | Multi-agency wraparound care (1) (S);  Care coordination at a client level (1) (W);  Partnerships with families (1; 2) (U);  Emphasis on rapport building (2) (W);  Trauma-informed care (3) (U; W);  Shared decision-making between clinicians and family members (3) (U; W);  Services and practitioners working together in a coordinated and client-focused way (5) (W);  Culturally appropriate care (5) (U);  Home visiting and a place-based service (8) (W);  Accessibility, flexibility, and service navigation (8) (S);  Effective inter- and intra- service cooperation (11) (W);  Service integration at a systemic level, trust and favourable interpersonal relations (12) (S);  Preparedness to share power and information (17) (W);  A whole of family care approach (18) (U);  Experienced and skilful clinicians (22) (W) | Healthy Homes and Neighbourhoods (HHAN) | Vulnerable Groups; CYP with complex care needs | Complex health and social care needs | long-term complex medical conditions | Australia |
| Enlow 2017  (73) | Increased ambulatory visits (4) (S);  Comprehensive access to ambulatory-based urgent visits is associated with better coordination (12) (S);  Improved receipt of preventive care leads to continuity of care (13) (W); | Primary care | Early years | General | General | US |
| Fleischman 2016  (74) | Delivery of health information and services via electronic communication technologies (tele-visits) (10) (S);  Tele-consultations (10) (S);  Promotion of communication between obesity specialists and PCPs (11) (W);  Tele-visits overcome the barriers of co-location (virtual co-location) (14) (S) | Primary care | CYP with Obesity | Obesity | Obesity | US |
| Friedel 2018  (75) | Coordination (between home care services, hospital services, schools and respite care services (1) (S);  Care (complex individualised, patient-centred curative and palliative care at home or in hospital settings) (1) (S; U; W);  Early referral (4) (U; W);  Training and on-going supervision (7) (W);  A culture of evaluation and improvement (7) (W);  Interdisciplinary meetings (11) (W);  Fundraising (lack of resources) (20) (S);  Communication (rising awareness among hospital teams) (24) (W) | Paediatric Palliative Care | CYP with oncology | Oncological disease | Oncology | Belgium |
| Fukkink & Verseveld 2020  (76) | Shared governance and collective decision-making (1) (W);  Information exchange between professionals within interprofessional groups (1) (W);  High level of density (1) (W);  Positive interpersonal relations and group reflection (1) (W);  Clearly defined goals (24) (W) | Early childhood education | Early years with special health care needs (SHCN) | Special needs | Special needs | Netherlands |
| Gajaria 2023  (77) | support in recovery by staff (3) (U; W);  Support from caregivers through communication, information provision (3) (U; W);  individualised care with considering complexities (3, 6) (U; W);  Gaining perspective on their illness through completing measures (6) (S; U);  group treatment (6) (U; W) | Secondary Care, Mental Health Centre | 14-18, CYP seeking services for a depressive disorder at the Centre for Addiction and Mental Health Depressive disorder | Mental health | Mental Health, Depression | Canada |
| Garber 2021  (78) | Nurse as a tele-presenter (1) (W);  Interpersonal collaboration (2) (W);  Advanced interprofessional training and education in integrated care (7) (W);  Telehealth (10) (S);  Electronic scheduling platform (10) (S);  Hub-and-spoke model (12) (U; W);  Acute and chronic care management (13) (S; W);  Interprofessional leadership (17) (W) | School-based programme | School years | General | General | US |
| Garg 2019  (79) | Inter-sectoral co-ordination (1) (S);  Social services/welfare services (1) (S);  Community health (2) (S; U);  Immunisation (4) (S; U);  Physical examination and specific screening activity (4) (U);  Health supervision (4) (U; W);  Reinforce anticipatory guidance (4) (U);  Developmental surveillance (4) (U; W);  Early childhood education (5) (S; U);  Local level non-governmental organisations (8) (S);  Child and family psychosocial assessment (9) (U);  Care coordination (12) (S) | Primary care | Early years | General | General | Australia |
| Garrett 2020  (80) | Establishing and maintaining good relationships with staff from a broad range of agencies (1) (W);  Sharing common electronic health records (1; 10) (S);  Complex/involved interactions (2) (W);  Service flexibility (8) (S);  Facilitating access to internal/external services (8) (S);  Information sharing (detailed information exchange between organisations/staff members) (12) (S) | Primary care, Sexual and reproductive health, mental health services, and alcohol and drug services | CYP with MH problems | MH | Mental Health | New Zeeland |
| Gauger 2018  (81) | Collaborative models (2) (S) | Primary care, oral health care | CYP | Dentistry | Dentistry | US |
| Geist 2020  (82) | Shared care plans (1) (W);  Flexibility and adaptiveness of staff (3) (W);  The need for team members to be available and work differently with each other and with the patient (3) (W);  Ongoing training and monitoring (7) (W);  Evidenced-based care (7; 9) (S; W);  Ongoing quality assurance (9) (S);  Evidence-based protocols (9) (S);  Active care coordination and management (12) (W) | paediatric hospital-based outpatient clinic | CYP with MH problems, CYP with Diabetes | MH and type 1 diabetes | Mental Health; Diabetes | Canada |
| Gore 2016  (83) | Evaluation of care for future care provision (9) (S);  Report system by patients on medical journey not outcomes (tool PREM) (10) (S; U) | The Paediatric Critical Care and Neurotrauma Recovery Program (PCCNRP) | Early years | Post-intensive care syndrome (PICS) | Post-intensive care syndrome (PICS) | UK |
| Goyal 2016  (84) | Early Detection and Prevention (Early assessment and intervention to prevent crises) (4) (S; U);  Linking families with services (8) (U);  Proactive telephone outreach to socially high-risk newborns (10) (S; U);  Systematic approach to newborn care (12) (S) | Primary care | Early years | General | General | US |
| Gratale 2022  (85) | Primary care is ideally positioned to promote wellness and a holistic approach to health for the paediatric population (1) (S);  Address social drivers (2) (S);  Care coordination and connection to social services (2) (S);  Focus on primary care (4) (S);  Health sector collaborate with other sectors (4) (S);  Early Detection and Prevention (Early assessment and intervention to prevent crises) (4) (S; U);  Primary care is important to transformative paediatric payment and delivery models (4) (S);  (Two-generation approaches) - transformative models (13) (S);  Value-based payment models (defined as those in which providers are paid based on patient outcomes) (20) (S);  Implementation of cost measures: a ‘net present value of care’ measure that includes actual short term healthcare savings and predicted savings over a specified set of years on the basis of intermediate health outcomes achieved (20) (S) | Primary Care | CYP | General | General | US |
| Greene 2016  (86) | The engagement of clinical and professional leads in the process (1, 17) (W);  The synthesis of outcomes in joint planning (1) (W);  Need for shared vision across multiple professional disciplines (2) (W);  Relationship-based approach (2) (W);  Empowerment through ‘bottom-up’ approaches in the development and improvement of health and social care services in their local area (3) (S; W);  Learning of staff (7) (W);  Evaluation (annual and performance reporting) (9) (S; W);  Communication and language with service users (11) (U);  Leadership (community lead, family lead, clinician lead (17) (W);  Strategic planning (17) (W);  Leadership is in relation to the management of budgets, and the ability to realise financial (rather than just structural) integration health and social care integration (17) (S);  Health and social care integration (17) (S);  The terms and conditions of existing staff in newly integrated structures (19) (W);  Motivation of professionals (21) (W) | Freestanding Practices | CYP with MH problems | MH | Mental Health | US |
| Griffin 2019  (87) | Personal and professional training (7) (W);  Leadership (community lead, family lead, clinitian lead (17) (W) | All | CYP | General | General | UK |
| Grimes 2018  (88) | Patient-centred delivery system for at-risk youths (3) (U);  Development of individualized treatment recommendations (3) (U; W);  Clarifying the right diagnosis for youths (3; 24) (U; W);  Brief interventions onsite at the primary care clinic (4; 14) (U; W);  Individualized hand-offs to treatment resources (reduce the need for families to start over) (5) (U; W);  Achievement of a high level of diagnostic clarity sooner (6) (S);  Outcome-based reimbursement (9; 20) (W) | Primary care | CYP with MH problems | MH | Mental health | US |
| Haberger & Venable 2018  (89) | Telephone consultation line for pediatric medical providers across the state (11; 21; 22) (U; W);  Co-located services in the pediatric primary care office (14) (S);  Resource mapping; training for primary care providers, and a collocated MSW intern training program (14) (S; W);  Assessment through A5 application: "assess-advise-agree-assist-arrange"-"Gather info"-"Screening"-"Telephonic outreach " - "Change motivation" (14) (S);  MD BHIPP has four components: a child psychiatry access program (CPAP) (14) (S) | primary care | CYP with MH problems | MH | Mental Health | US |
| Halberstadt 2023  (90) | four-component model (vision; process; partners; finance) (1) (S; U; W); Setting up a learning community of professionals to exchange knowledge, experiences and tools (1; 2; 6; 7; 11; 15) (S; W); Collaboration between municipalities and Care for obesity programme though developing a national model (2) (S; W); Creating commitment (by presentations, conferences, etc) (8; 19) (S; U); coordinated professionals (16) (S; W) | Primary Care | CYP 0-19, with overweight and obesity | Overweight and Obesity | Obesity | Netherlands |
| Hall 2020  (91) | Multiple stakeholder involvement (1) (S);  Access to and input from specialised care (2) (S);  Evidenced-based interventions and behavioural strategies (9) (S) | The Paediatric Critical Care and Neurotrauma Recovery Program (PCCNRP) | Early years | Post-intensive care syndrome (PICS) | Post-intensive care syndrome (PICS) | US |
| Hanson 2019  (92) | Trauma-focused training and learning (7) (W) | Mental health care and social services | CYP with MH problems | MH | Mental Health | US |
| Hart 2019  (93) | Team-based approach (1) (W);  Longer appointment times may be a necessary component of high-quality transitional care (6) (S; U);  Division of care coordination tasks based on care coordinators’ training (7) (W);  Learning from practice and learning from families (7) (U; W);  Focus on the whole patient (12) (U);  Gradual transition model: taking time for patient care (13) (S);  Frequent return visits (13) (S; U);  Care coordination: leadership and key coordinator (16) (W) | Primary care | CYP with long-term conditions | Childhood-onset chronic illnesses | Long-term complex medical conditions | US |
| Henderson 2019  (94) | Effective collaboration and communication between providers (1) (W);  Decentralised decision-making opportunities (1) (S);  Access to a wide range of coordinated services in one place (1) (S);  Relationship trust (1; 2) (U; W);  Previous positive working relationships (2) (W);  Respectful and open atmosphere to reduce power imbalances (3) (S; U; W);  Youth as co-investigators and co-creators of the initiative (3; 18) (U);  Co-design and co-production of the initiative in partnership with families and service partners (5) (S; U; W);  Co-development and co-design (5) (S; U; W);  Comprehensiveness in implementation planning (6; 7) (S);  Adaptability of the model (8) (S);  One step access to walk-in services (8) (S);  Leadership (community lead, family lead, clinician lead) (17) (W);  The need for a ‘champion’ (17; 23) (W);  Investment of resources from partnering organisations (20) (S);  Re-structuring existing services rather than building new (20) (S);  Complementary expertise (22) (W) | Mental health and substance use services; Primary health care; Social and vocational services. | CYP with MH problems | MH | Mental Health | Canada |
| Hetrick 2017  (95) | Integrated in community (4) (S);  Timely response (6) (S);  Extended hours of operation and cost free (8) (S);  Accessibility, flexibility, and service navigation (8) (S; U);  Multiple entry points (self-referral, drop-in) (8) (S; U);  Ongoing evaluation (9) (S);  Safe and youth friendly environment (19) (S; U);  Recreational and hang-out space (19) (S; U);  Ensure privacy (19) (S; U) | Primary mental and physical health care services | CYP with MH problems | MH | Mental Health | UK, Australia, Canada, New Zeeland, US |
| Hillis 2016  (96) | Bringing pediatric psychology to the “front lines” of community care (2; 6; 22); (W);  Screening of risks (4; 9) (S);  Prevention in the form of anticipatory guidance and identifying mothers at risk for depression (4) (S; U) | Primary care | CYP with complex care needs | Complex needs | Long-term complex medical conditions | UK |
| Hine 2017  (97) | Improved quality of care (2) (S);  Integration of behavioral health into primary care (2) (S);  Routine assessment (4) (S; U);  Training of behavioral health providers (7) (W);  Knowledge of psychological principles (7) (W);  Better access to care (8) (S; W);  Telemedicine (10) (S) | Paediatric primary care clinic | Early Years focus; CYP with MH problems | MH, Mental behavioural health (BH) | Mental health | US |
| Hoff 2020  (98) | Early assessment and intervention to prevent crises (4) (S; U);  Face-to-face interactions (5) (U; W);  On-going follow-up (13) (U; W);  Co-location (14) (S) | Primary care | CYP with MH problems | MH (Anxiety, depression, attention deficit/ hyperactivity disorder, disruptive behaviour) | Mental Health | US |
| Holder-Niles 2017  (99) | Referral to and coordination with social services (1) (S; W);  Community-based agencies (1) (W);  Improved follow-up care in-depth diagnostic and family needs assessment (4; 6; 9) (S; U; W);  Specialized asthma education to families (5) (U);  Incorporate community health workers and provide in-home asthma education (5) (U);  Home visiting programs (8) (S; U) | Urgent care, emergency department care | Vulnerable groups; CYP with Asthma | Asthma | Asthma | US |
| Holmen 2023  (100) | Care provision through intersectoral teams (1; 2; 12; 15) (S; W); Establishing basic structures for cooperation across interdisciplinary teams (1; 2; 12; 15) (S; W); Integrates mental health services in general services (1; 4; 6; 12) (S; W); Approaches youth in their environment to improve understanding about their context (3; 8) (S; U); Enhances early intervention in municipalities and supports prioritisation of care for the specialist sector (4) (S); Easy access provision (phone, short waiting time, limited need for assessment forms to fill out) (8) (S; U); care includes housing; shared responsibilities across different levels of care by avoid unequal access to services (8) (S; U; W); systematic care delivery provision (8) (S); flexible and low threshold care (8) (S); tailored care to children and youth who need it most (13) (S; U); Centralised model (16) (S; W); care reaches beyond the age of 18 (8) (S; U) | Primary Care | CYP | Mental Health | Mental health | Norway |
| Hostutler 2021  (101) | Community-hospital – accountable care organisation partnerships (2) (S);  Measurement-based care (4) (S);  Evaluation of outcomes (7) (S);  Creation of their own electronic health records –registry using REDCap (10) (S);  Ongoing operational and clinical consultation (11) (U; W);  Provide integrated behavioural health consultants (11) (U; W);  Tailored to the developmental needs of children (21) (S; U; W) | Paediatric primary care | CYP with MH problems | MH (behavioural health) | Mental Health | US |
| Howard 2017  (102) | Care coordination (2) (S);  Family-based care capacity building (3) (U);  Interprofessional approaches (14) (S; W);  A central role of nurses (16) (W) | Acute care paediatric hospitals | CYP with complex needs; Early years focus | Cerebral palsy | Cerebral palsy, disability | US |
| Hughes 2017  (103) | Knowledge and decision-making sharing (1) (S);  Horizontal integration (2) (S);  School and medical collaboration (BMI screening) and vending machine removal (2) (S) | School; Paediatric primary care | CYP with complex needs | Obesity | Obesity | US |
| Huryk 2021  (104) | Knowledge-sharing practices (1) (W);  Access to and input from specialised care (2) (S);  Consistent communication (2) (W);  Caregivers can participate in programming (3) (W);  Parental empowerment (3) (U);  Family involvement (5) (U);  Partial hospitalization (PHP) (5) (S; W);  Intensive outpatient programs (IOP) (5) (S; U) | Partial hospitalization (PHP) and intensive outpatient programs (IOP) | CYP with MH problems, CYP with Compulsive Eating Disorders | Anorexia nervosa; MH | Mental Health; Compulsive Eating Disorder | US |
| Hynek 2020  (105) | Information exchange between professionals within interprofessional groups (1) (W);  Knowledge transfer (individuals with different professional backgrounds and knowledge and skills working together (1; 7) (W);  Dialogue conferences (7) (W);  Involvement of staff in the development of policies and procedures (7; 21) (W);  Equality (6) (S);  Guidelines regarding confidential information (7; 19) (S);  Clear professional roles and responsibilities (17) (W);  Leaders supporting interprofessional collaboration in the organisation (17) (W);  Strong and supportive management (17) (W) | Primary schools | School years | General | General | Norway |
| Iachini 2016  (106) | Collaboration through learning (2; 5) (W) | Community services, primary care | CYP with obesity | obesity | Obesity | US |
| James 2023  (107) | shared action plan for children (1) (S; W); screening for housing instability and food insecurity (4) (U; W); introduction of performance measures: screening for clinical depression (4, 9) (S; U; W); introduction of performance measures: kindergarten readiness promotion bundle (4, 9) (S; U; W); increasing awareness measures (6) (S; W) | Primary Care | CYP | General | General | US |
| Johnson 2020  (108) | Individual and organisational values (1) (S; W);  Organisational workplace culture (1) (S);  Access to and input from specialised care (2) (S; U);  Person-centredness (3) (S);  Families/carers must be empowered and engaged (3) (U);  Providing a single point of contact (5) (S; U; W);  Effective organisational systems, support and structure (10) (S) | Tertiary Paediatric Hospital Setting | CYP with complex health needs | Complex health needs/ children with medical complexity | Long-term complex medical conditions | Australia |
| Johnston 2016  (109) | Reliable team interactions between physicians and staff (1; 15) (W);  Accountability (2) (S);  Provision of all patients with a dedicated oncologist (16) (S; U);  Governance and workload balance (19) (S; W) | Paediatric Oncology Clinic Care Model | CYP with Oncology | Oncology | Oncology | Canada |
| Jones 2023  (110) | Early Identification (4) (S); Risk stratification (6) (S; W);  Service integration (12) (S) | Primary Care | 0-21 with complex care needs | Mental Health, special health care needs, subtance use disorder | Mental health | US |
| Jorina 2016  (111) | Medical and behavioural care providers (1) (S; W);  Community organizations and health policy experts (2) (U; W);  The care coordination key elements framework based on triple aim (2) (S);  Creation of a state-wide partnership including parents and family advocates (2) (S; U; W) | Mental health care; Primary care | CYP with MH problems | Behavioural health, MH | Mental Health | US |
| Kaehne 2016  (112) | Maintaining a sense of common purpose (1) (W);  Commitment and willingness to change (2) (W);  Perceptions of shared vision and goals (2) (W);  Information exchange between organisations (10) (S) | All | CYP | General | General | UK |
| Karwacki 2021  (113) | Multispecialty consultation or morbidity-oriented care (1) (S);  Holistic management of disease (1) (S; W);  Supervised monitoring (1) (W);  Longitudinal coordinated care (1) (S; W);  Regular clinical visits: ambulatory consultation and consultations both “on demand” and through electronic media (3) (S; W);  Psychological support to family (3) (U);  Education (6) (W);  Telemedicine “medical reviewer NE-coordinator” which supervises patient-oriented care (10) (S; W) | Primary care | CYP with neuro-fibromatosis | Neuro- fibromatosis type 1 and related RAS opathies | Neuro-fibromatosis | Poland |
| Kearney 2019  (114) | Knowledge and decision-making sharing (1) (S; W);  Horizontal integration (2) (S);  School and medical collaboration (BMI screening) and vending machine removal (2) (S); | Primary care; Secondary care | CYP with MH problems | Behavioural health, MH | Mental health | US |
| Kirby 2021  (115) | Shared professional responsibility and practices: governance – leadership group (1) (W);  Mutual trust (1) (W);  Perceptions of shared vision, goals, and a common value base (2) (W);  High levels of trust between parties (2) (W);  Embedded in role descriptions (2) (W);  Stronger connections and partnerships: involvement of cross-sector and multidisciplinary team (2) (W);  Stakeholders working collaboratively across disciplines, settings, levels of care, and sectors (2) (S);  Data sharing (10) (S; W);  Empowerment of service users’ information (3) (U);  Exchange and individualised care plans: individualised care plans, developed with family (family centred) (3; 5) (U; W);  Early detection and prevention: screening for health needs (4) (S; U; W);  Early assessment and intervention to prevent crises (4) (S; U; W);  Accessibility: better access to care (7) (S; U; W);  Building cooperation in the multidisciplinary cross-sectoral team (11) (S; W);  Important information about families - picture of family life (11) (U);  Case management (12) (W);  Continuity of staff/leadership (13) (W);  Co-location in certain services (14) (S);  Designated Coordinator/Navigator/Key Worker/Case Manager: help guide patients/caregivers navigate through the healthcare system (16) (W);  Trusted member of the community (16; 19) (W);  Leadership: leaders supporting interprofessional collaboration in the organisation (17) (W);  Finance: develop financially sustainable models for integrated primary care (20) (S);  Knowledge of roles of each service (23) (W) | Cross-sectoral care, primary care | Early years; vulnerable groups | Complex care needs, chronic health condition, early years | Long-term complex medical conditions | Australia |
| Kodish 2019  (116) | Coordination leads to co-location and leads to integration (1) (W);  Training of specialist staff, pediatricians, child and adolescent psychiatrists, and other behavioural health providers develop new skills and evolve practice processes to engage youth with evidence-based care approaches (7) (W) | Primary care depression screening | CYP with MH problems | Major depressive disorder (MDD) , MH | Mental health | US |
| Kolko 2020  (117) | Physician-parent shared decision-making during treatment planning (2) (U; W);  Educational/medication support strategies (2; 6) (S);  A culture of improvement and evaluation (7) (S);  Training and on-going supervision (7) (W);  Consultation, infrastructure support and technology (8) (S);  Tailored and individualised goals (23) (S) | Paediatric primary care | School years; CYP with ADH; CYP with MH problems | Attention deficit hyperactivity disorder (ADHD); MH | Mental health; LD & Autism | US |
| Kolko 2022  (118) | Delivery system redesign (2) (S; W);  self-management support (3) (U);  monitoring (4; 6) (S; U; W);  Use of evidence-based interventions (9) (S); features that include team-based care (15) (W) | Primary Care | 5-12 years old, Children with behavioral needs | ADHD; DBD | LD & Autism | US |
| Lail 2017  (119) | Medical provider available 24 hours per day (5) (U; W);  Promotion of adherence between staff and patients (5) (U; W);  Use of population registry helps prioritising severity of medical needs (6) (S);  Staff training (7) (W);  Regular practice-team meeting to problem solve and assess progress (7) (W);  Use of “plan-do-study-act cycles” strategy (9) (S);  Development of electronic tracking to prompt visits (10) (S) | Paediatric primary care | CYP with Complex needs; Early years | Medical complexities | Long-term complex medical conditions | US |
| Lambert 2017  (120) | Shared caseload (1) (S; W);  Shared decision-making (2) (S; W);  Collaboration with social work (2) (S);  Case management (2) (W);  Staff training (diagnosis-specific training) (4) (W);  Early detection (4) (S; U);  “No drop-out" policy (5) (S; U);  Home treatment (8) (U; W);  Extended hours (8) (S);  High frequency face-to face contacts (11) (U; W) | Youth psychiatry care | CYP with MH problems | MH | Mental health | Germany |
| Lambert 2018  (121) | Interventions to improve service utilization; early detection and pathways-to-care (4) (S);  Implementation of a cross-age and interdisciplinary mobile early detection team (4) (S; U);  Four-year trialogue interventions to improve mental health literacy, stigma and service utilization (a trialogue ‘awareness campaign’ including cinema spots, city-light posters) (7) (S);  (Expansion of the early detection service for psychosis to a cross-age and interdisciplinary early detection service for all mental disorders (aged 12–29 years)); expansion of the pre-existing catchment area network to improve the service utilization (7) (S; U) | Therapeutic assertive community treatment | CYP with MH problems | MH | Mental health | Germany |
| Lamson 2022  (122) | Primary care/behavioural healthcare collaboration (basic collaboration from a distance (separate systems) - basic collaboration on-site (consult model only; regular communication) - close collaboration in a partly IC system – close collaboration in a fully IC system (shared systems and facilities) (2) (S; W);  Screening (4) (S);  Brief intervention and/or referrals for specialized services (4) (S);  Telehealth (10) (S);  “Champion” within the system (17) (W);  Voice and equal participation to all team members (21) (W) | Primary care / behavioural healthcare | CYP with complex care needs | Complex health care needs | Long-term complex medical conditions | US |
| Lauerer 2018  (123) | Collaborative consultation (2) (S; W);  Off-site mental health provision (8; 13) (S; U; W);  Phone consultations (8) (S);  Referral model (13) (S; W) | Primary health | CYP with MH problems | Behavioural health disorders, MH | Mental health | US |
| Lavigne 2016  (124) | ‘Information exchange (1; 10) (W);  ‘Care process’ (2) (S);  ‘Child’s environment’ (3) (U);  Individual: person-centred care in a single process across time, place, and discipline (5) (U; W);  ‘Preconditions’ (8; 12) (S);  Broad assessment of problems and needs (9) (W);  ‘Interprofessional collaboration’ (14) (W);  Safe network (15) (W);  (S);  ‘Expertise’ (22) (W);  Clear clinical pathways (21) (S; U);  ‘Expertise’ (22) (W);  ‘Professional identity’ (23) (W) | Paediatric Primary Care | Early years focus; CYP with MH problems | MH | Mental health | US |
| Lawler 2017  (125) | Horizontal integration (2) (S);  Training of staff (7) (W) | Primary care | Early years; CYP with MH problems | MH | Mental health | US |
| Lawn 2018  (126) | Quality use of medicine to minimise medication errors in the community (2) (S; W);  Self-management interventions aim to help individuals better manage their medical treatment and cope with the impact of the condition on their physical and mental well-being (3) (U);  On-going monitoring and follow-up (5) (S; W);  Chronic condition self-management education (5) (U);  Improved discharge processes (13) (S) | Primary care and hospital systems | CYP with long-term conditions | Chronic conditions (heart disease, diabetes, respiratory, and behavioural disorders) | Long-term complex medical conditions | Australia |
| Leahy 2019  (127) | Patients presenting before surgery with ≥1 predefined medical comorbidity were triaged to the intensive care unit (ICU) postoperatively, while patients without severe systemic disease were triaged to a lower-acuity floor for overnight observation (9) (S; U);  Standardized perioperative (preoperative, intraoperative, and postoperative) protocols were developed, with a focus on preoperative risk stratification (12) (S) | Perioperative Surgical Home (PSH) | CYP with pre and post operative care | Laryngeal cleft undergoing endoscopic surgical repair | Perioperative care | US |
| Lee 2021  (128) | Developing multi-stakeholder partnerships (1; 15) (W);  Developing strong and enduring partnerships (2; 15) (W);  The importance of intervening in multiple settings, and ongoing implementation and evaluation strategies (9) (S);  Strong and complementary, interagency connections and partnerships (15) (W);  Culturally tailored, clearly defined roles (21) (S; W) | Community and primary care | Vulnerable groups, early years | Obesity | Obesity | US |
| Lenton-Brym 2020  (129) | Access to and input from specialised care (2) (U);  Youth-friendly primary care systems (3) (U);  Assessing the need for hospitalisation (4) (S; U);  The role of primary care providers in educating patients and families (5) (U; W);  Enhanced involvement of primary care providers in treating youth with eating disorders (7) (U; W);  Restoring weight and providing health maintenance and follow-up (13) (U);  Monitoring and managing medical complications and making referrals and coordinating an interdisciplinary team (13) (S; W) | Primary care | CYP with compulsive eating disorders; CYP with MH problems, early years focus | Eating disorders; MH | Mental health, Compulsive Eating Disorder | Canada |
| Lin 2021  (130) | A tertiary-care-based nurse practitioner (7; 16) (W);  Coordination across multiple systems of care (12) (W);  Lifelong child-, youth- and family-driven care and supports that optimize health and quality of life (13) (S; U);  Partnership with children, their family and multidisciplinary providers (18) (U) | Tertiary-care; Children’s developmental and rehabilitation services | CYP with complex needs | Complex needs, | Long-term complex medical conditions | Canada |
| Lindsay 2016  (131) | A multidisciplinary team (1) (W);  Organisation of care: lifespan approach (2) (S);  Advocacy for the model and inter-agency partnerships (2) (W);  Personal: transition readiness, self-care, life-social skills (3) (U);  Integrated approach which promotes essential self-advocacy, self-care and life skills (3) (S; U);  Necessary skills through care provision (5) (U);  Developmental process of growing up (6) (S; U);  Availability of care (8) (S; U);  Location of clinics (8) (S);  Evaluation of a new model (9) (S);  Establishing communication channels (11) (W);  Coordination across multiple systems of care (12) (S; W);  Continuity in care from paediatric to adult health care (13) (S; U);  Continuity of care (13) (S);  Relational inter-professional teamwork (15) (W);  Two new introduced specialists: a cross-appointed nurse practitioner and life skills coach (16) (W);  Structural leadership (17) (W);  Funding (20) (S) | Secondary care | Adolescents with disabilities | Spina bifida | Spina bifida | Canada |
| Long 2022  (132) | Parent-directed early developmental screening (9) (S; U); follow-up care (13) (S; U) | Emergency Care, Primary Care | Early years | young children with neurodevelopmental vulnerability | LD & Autism | Australia |
| Lopez-Carmen 2019  (133) | Intervention delivery via community workers (1) (W);  Intervention delivery via external workers (1) (W);  Interdisciplinary delivery (1) (W);  Empowering families (3) (U);  Individual counselling (3) (U; W);  Adaption of care to indigenous sociocultural specificities (3) (U);  Strengthening culture and identity (3; 11) (S; U; W);  Engaging community (18) (U);  Staff and organizational capacity building (21) (W) | Primary care | Adolescents with disabilities; CYP with MH problems | MH | Mental health | Canada, Australia, New Zealand, Norway and/or the United States |
| Loria 2021  (134) | Team of primary care and behavioural health clinicians work together with patients and families (1) (W);  Electronic medical record system (1) (S);  Patient-centred care (1; 5) (S; W);  Interdisciplinary team (medical, mental health and child welfare professionals) (2) (W);  Flexibility of integrated visits depending on a patient's needs (3) (U);  Evidence-based behavioural treatment (4) (S; W);  Revision of health history from birth (13) (U);  Each child is assigned a case worker (16) (U) | Primary care | Early years; Vulnerable groups | Early years; trauma | Early years; Trauma; Mental health | US |
| Love 2022  (135) | Engagement (1) (S; U);  Nursing and social work collaboration and care coordination (1) (W);  Interdisciplinary team (medical, mental health and child welfare professionals) (2) (W);  Produce individualized recommendations (3) (U; W);  Continuous care, and fewer appointments (13) (S);  The electronic medical record (10) (S);  Clinical restructuring of care (12) (S);  Care plan – road map (12) (S; U);  Continuous care, and fewer appointments (13) (S);  Centralized care with access to a single person coordinating clinic visits (16) (S; W) | Primary care; Secondary care | CYP with cerebral palsy | Cerebral palsy | Cerebral palsy | US |
| Luke 2020  (136) | A team of providers working collaboratively across disciplines, settings, levels of care, and sectors (1) (W);  Person-centred and holistic approach (1) (S; U);  Comprehensive delivery of quality services (1) (S);  Access to services and resources (8) (S; U);  Flexibility of communication methods (e.g., email, phone) (8) (S; U);  Reducing barriers to care (8) (S; U) | All | CYP with Complex needs; Early years focus | Complex care | Long-term complex medical conditions | Canada |
| Luzi 2018  (137) | Collaboration: values and ethics (i.e., working with individuals of other professions to maintain a climate of mutual respect and shared values) (2) (W) | All | CYP with ASD and ADHD | ASD (Autistic Spectrum Disorder) and ADHD (Attention-Deficit/Hyperactivity Disorder), MH | LD & Autism; Mental health | Italy |
| Luzi 2019  (138) | Care coordination between patients and family-centred team (1) (W);  Horizontal integration (2) (S);  Medical crisis procedures (4) (S);  Screening, assessment and referral; community based services (4) (S; U);  Managing whole child pathway (6) (U);  Access to care (8) (S; U);  Shared ICT systems (10) (S);  Multi-disciplinary teams (15) (W) | Primary and secondary care | CYP with complex needs | Complex care needs | Long-term complex medical conditions | 30 EU/EEA countries |
| Lyles 2017  (139) | Provider knowledge (1) (W);  Effective communication (1) (U);  Family education (5) (U);  Insurance (19) (S);  Health care capacity (21) (S) | All | CYP with long-term conditions | Special health care needs or chronic conditions | Long-term complex medical conditions | US |
| Lyon 2016  (140) | Receive skills training in mental health interventions (7) (W);  Patient-centred care (1; 5) (U);  Multidisciplinary team (1; 2) (W);  A centralized mental health telephone program (2; 10) (S; W);  Stepped care” : advancement of treatment intensity for service recipients who are not improving (2) (S; U);  Collaboration (CC model): a team of coordinated providers (5) (W);  Accountability through consultation and supervision (6) (S; W);  Measurement-based treatment models (9) (S; W);  “Population-based approach” to tracking outcomes (9) (S);  Use of evidence-based interventions (9) (S);  Client education and engagement (18) (U) | Primary care | CYP with MH problems | MH | Mental health | US |
| Lyon 2018  (141) | Organisational culture (shared values, beliefs, implicit norms that guide behaviour) (1) (S; W);  Organisational climate (employee-shared perceptions of the work environment) (1) (S);  Implementation –specific organisational factors: -implementation climate (staffs’ shared perceptions of the extent to which EBP implementation is expected) (1) (S);  Inter-organizational context (connections among organizations or units of the outer and inner settings) (2) (S);  Implementation leadership-implementation citizenship behaviour (going beyond the call of duty to support implementation) (17) (S; W) | Education sector mental health | CYP with MH problems | MH | Mental health | US |
| Lyon 2019  (142) | Shared treatment plans (1) (W);  Mechanisms for increasing treatment intensity (2; 9) (S);  Horizontal integration (2) (S);  Multidisciplinary teams (2) (W);  Stepped care”: advancement of treatment intensity for service recipients who are not improving (2) (S; U);  Measurement-based care (4) (S);  Early identification and treatment (4) (S; U; W);  Well-defined roles (7) (W);  Accessibility: better access to care (8) (S; U);  Systems to monitor and track progress (9) (S);  Use of evidence-based interventions (9) (S);  Population-based approach (9) (S; U);  Feedback to front-line professionals (11) (W);  Team-based care (15) (W);  Care managers (16) (W);  Established infrastructure (25) (S; W) | All | School years | General, MH | General; Mental health | US |
| Malas 2019  (143) | Strong collaboration (1) (W);  Multimodal approach (1) (S);  Interdisciplinary teams (1) (W);  Vertical integration (although partnerships outside of health care) (2) (S);  Embedded psychiatric care (6) (S);  Training and support (BHCs and PCPs) (7) (W);  Existing resource support (8) (S);  Signposting to local resources (8) (S; U);  Increased access (8) (S; U);  Service flexibility (8) (S);  Needs-based care (9) (S; U);  Responsive consultation (11) (U; W);  Sustainability (19) (S);  High quality service (22) (W) | Primary Care | CYP with MH problems | MH | Mental health | US |
| Manente 2017  (144) | Play therapy and preoperative teaching for families; empowerment of families through teaching (5; 6) (W);  Families awareness (6) (U);  Log keeping (6) (U; W);  Nurse preparation (7) (W) | Paediatric unit | General | General | General | US |
| Mathias 2022  (145) | training of staff (7) (W); one-stop health and socials service delivery (8) (S; U); low barrier services (8) (S; U); solution-focused brief therapy (8) (S; U); walk-in councelling (8) (S; U); co-location of services in one place including physical and sexual health, mental health, substance use, peer support, social services (14) (S); staff collaboration (15) (W) | Primary Care | 12-24 years old | Mental health | Mental Health, Substance use disorder | Canada |
| Mautone 2021  (146) | Referral support and follow up training (3; 5) (U; W);  Medication management (4) (U);  Progress monitoring (4; 6) (S; U; W);  Warm handoff consultation (5) (U; W);  Brief psychotherapy (6) (U);  Evaluation: provider adherence, monitor productivity, monitor service utilization (9) (U);  Intake/treatment (13) (U; W) | Primary care | CYP with MH problems | Behavioural health care, MH | Mental health | US |
| Mazur 2021  (147) | Embedded specialists which provide counselling (1; 2; 8) (U; W);  Coordinating referrals to external specialists (1; 2; 3) (W);  2nd model: collaborative relationships with pediatric primary care teams to provide telephone consultations (1; 2) (W);  3rd model: embedded specialists (1; 2; 21) (W);  Primary care is responsible for detection, initial assessment (4) (S);  Development, implementation of a treatment plan (4) (S);  Coordination of referrals to specialists as needed and monitoring progress (4) (S);  Continuing education (6) (W);  Patient psychiatric evaluation (9) (S; U);  Expedite outpatient psychiatric evaluation (9) (S; U);  Ethical guidance considerations: The very act of delivering team-based interprofessional care will inevitably create conflicts between different guild-specific ethical guidelines (19) (S) | Paediatric Mental Health Care | CYP with MH problems | MH | Mental health | US |
| McLeigh 2022  (148) | co-located services (14) (S);  collaborative care; psychiatrist on as-needed basis (14; 16) (S) | Primary Care; Behavioural Health Clinics | CYP | General | General | US |
| McPherson  2017  (149) | Collective impact-focus on community not individuals (1; 2) (S; W);  Horizontal integration (2) (S);  Relationships as a trigger (place-based and collective impact approaches): a spirit of goodwill, high levels of trust between parties, norms of reciprocity and adaptability, a sense of obligation among group members, embedded ties through strong and enduring relationships (2; 8) (S; W);  Forming professional partnerships (2) (W) | Rural high school | CYP | General | General | Canada |
| Melamed 2022  (150) | Technology-enabled collaborative care-platform for assessment (10) (S);  Consultation (11) (W) | All | CYP with MH problems | Psychotic disorder, MH | Mental health | Canada |
| Messiah 2016  (151) | Coordinated, collective partnerships in multiple sectors such as government, health care, school/afterschool, workplace, and the community (1; 2) (W) | Primary care; community-based care | CYP with obesity | Obesity | Obesity | US |
| Moisan 2021  (152) | The implementation of an integrated youth team through strategic, tactical and operational control rooms to offer coordinated services (the central component of this integration project) (2; 18) (S);  Problem-solving cycle: plan, do, check, adjust (9) (S);  Control rooms for assessing current performance among team members (9) (W);  The status sheet (11) (S; W); | Primary care, community-based care | Vulnerable groups | General | General | Canada |
| Montejo 2021  (153) | Information sharing through email (to pediatricians of the links to the BICP, highlighting the core features of the protocol for the management of the AB) (1; 11) (W);  Families: patient journey map (3; 13) (U);  Information sheets for families (3) (U);  Information provision to families about prevention development of the eSanoweb page (which includes all the AB pathway tools as well as educational videos for parents dissemination by e-mail to pediatricians of feedback on levels of and/or improvement in pharmacologic prescribing) (5) (U);  Display of an educational poster in waiting and consultation rooms with key messages for families (5; 6) (U);  Display of an informative poster in the lobby of the hospital during AB seasons (5; 6) (S; U);  Campaigns on social media with educational messages targeting families drafted by the health care professionals themselves (5; 6) (S; U);  Inclusion in the organization’s computer program of a pop-up window associated with the diagnosis of AB that automatically displays the most important documents associated with pathways (6; 10; 11) (S; W);  Information-training sessions including review of current evidence (7) (W);  Display of posters with the decision tree (11) (W);  Badges for uniform “team on the pathway” (15; 23) (W);  Providers: focus-group methodology (18) (S);  Involving patients and their families in quality teams (18) (U) | Emergency care units | Early years; CYP with bronchiolitis | Acute bronchiolitis | Acute bronchiolitis | Spain |
| Moss 2021  (154) | Coordination (1) (S);  Management of referrals of children and young people to access medical care (1; 4) (S; U);  Co-designing innovation requires sustained commitment long-term (2; 13; 19) (U);  Shared governance and investment are essential to effect and sustain inter-agency change (1; 20) (S; W)  Supervised health checks (4) (S; U);  Health management plan developed (5; 6) (S; U);  Consumer engagement is essential to implement sustainable healthcare interventions (18) (U); | Primary care | Vulnerable groups, early years focus | General | General | Australia |
| Njoroge 2016  (155) | Coordinated/telephonic: increased collaboration between PCPs and behavioral health provider (1; 10) (S; W);  Integrated: direct collaboration and integration in medical team (2) (S);  Potential co-joint treatment planning (2; 14) (S; U);  Warm-handoffs for direct patient engagement (3; 5) (U);  Support for remote consultation, including psychiatry and medication management (3; 10) (S; U);  No face-to-face consultation and integrated care coordination (10) (S; U);  Co-located: Located in the same physical space (ideally leading to an ease in referral and patient comfort) (14) (S; U; W);  Office space in primary care practice (21; 25) (W) | Primary care | CYP with MH problems | MH | Mental health | US |
| Nooteboom 2020  (156) | Multiple professionals collaborating (1) (W);  A holistic, family-centred approach (1; 3; 5) (S; W);  Addressing a broad range of needs in a timely manner (1) (S; U);  Shared decision making (1) (S; W);  Referral (1; 2; 4; 13) (S; U; W);  Coherent, continuous, and coordinated care (1; 13) (S; W);  Interprofessional collaboration (2; 14) (W);  Comprehensive and up-to-date shared care plans (2; 6; 9) (W);  Strengthening the capacity of families (2; 3) (U);  Meeting youth at a location of their choice (3) (U);  Freedom to adapt treatment plans (3) (S; W);  Parental involvement in decision making processes (3; 18) (U);  Early detection and support (4) (S; U; W);  Collaboration between professionals and parents (5) (U; W);  Clear allocation of tasks and responsibilities (6; 23) (W);  Walk-in sessions (8) (U);  Providing up-to-date information on the availability of services (10; 11) (S; U);  Timely support across several life domains tailored to a family’s needs (13) (U);  Co-location of services (14) (S);  Ensure privacy (19) (U);  Privacy of family members during information exchange (19) (U) | Primary care | CYP with MH problems | Multiple health needs | long-term complex medical conditions; Mental health | Netherlands |
| Nooteboom 2021  (157) | ‘Information exchange’ (1; 10; 11) (W);  ‘Care process’ (Screening and assessment, Broad assessment of problems and the use of screening tools, StrongShared Care plan, Several perspectives and goals in a comprehensive care plan, Medium—strongReferral, Transition between care providers, Medium—strongTheme: Expertise Knowledge and training, Extending knowledge by means of training, StrongGuidelines, The use of evidence-based guidelines to support professionals, StrongSelf-efficacy, Confidence and comfort of professionals to provide integrated car) (1; 2) (S);  ‘Child’s environment’ (their description, and strength of evidence: Family-centered focus, A holistic approach on a family’s welfare, medium - strong) (3) (U);  ‘Preconditions’ (Time, Time to address a broad spectrum of problems and for inter-professional collaboration) (6) (S);  ‘Inter-professional collaboration’ (6) (W);  ‘Expertise’ (Expertise Knowledge and training, Extending knowledge by means of training, StrongGuidelines, The use of evidence-based guidelines to support professionals, StrongSelf-efficacy, Confidence and comfort of professionals to provide integrated care) (16) (W);  ‘Professional identity’ (Professional roles and responsibilities, Clarity and expectations about professional roles, sharing responsibility, StrongAttitudes, Attitudes and commitment towards integrated care and collaboration, StrongShared thinking, A shared foundation in thoughts, aims, priorities, and values, Strong—very strong; trust, respect and equality; mutual trust, respect for other professionals and perceived equality, Strong) (17) (W). | Multiple care providers | CYP with long-term conditions; CYP with complex needs | Complex and chronic problems | Long-term complex medical conditions | various |
| Otis 2023  (158) | Active engagement of family members or guardians (2; 4; 5; 6; 11; 18) (S; U);  Risk-prevention psychoeducation for guardians of individuals with suicide-related emergencies (2; 4; 5; 6; 18) (S; U);  Risk -targeted follow-up for caregivers of individuals with suicide-related emergencies (2; 4; 5; 6; 18) (S; U) | Acute Care | 8-25 years old, CYP with MH problems | CYP with Mental Health | Mental Health | US; Canada; Australia |
| Parikh 2021  (159) | Electronic health record (1; 10) (S);  Group and individual psychotherapy (3) (U);  Crisis evaluation (acute suicide risk evaluation) (4) (S; U);  Warm handoffs (5) (S);  Informal consultation (11) (U); | Mental health care, primary care | CYP with MH problems | behavioural health (BH), MH | Mental health | US |
| Parker 2020  (160) | Focus on inherent strengths (strengths-based approach) (1) (S);  Shared plan of care (1) (W);  Provision of necessary resources for the child and family (8; 25) (U);  Effective communication between parents and providers (11) (U);  Family-driven communication tool between parents and providers (11) (U) | Multiple care providers | CYP with ASD | ASD | LD& Autism | US |
| Parkhurst 2023  (161) | hub-spoke approach supporting a small and independently owned primary care setting (2) (S; U; W);  access to evidence-based psychotherapy care (8) (S; U);  private practices unlinked to primary care practices (8) (S);  A regional network of psychotherapy providers to facilitate the navigation of outpatient psychotherapy care with timely psychotherapy referrals and to support the collaborative care model (11; 12; 13; 15) (S; W) | Private primary care | 6-18 years old, CYP with mental health problems | CYP with Mental Health, ADHD | Mental Health, LD& Autism | US |
| Parsons 2021  (162) | Information systems-electronic medical record, electronic prescriptions (1; 10) (S);  Integrated provider systems (1) (S);  Organisation of health care (1; 2) (S);  Coordination with client/family/school health systems (1) (U; W);  Referrals to specialists for consult (1; 2) (S);  Referrals for behaviour therapy (1; 2) (U);  Case management (2) (W);  Decision support (2) (W);  Clinical guidelines-AAP/NICHQ; templates for primary care screening tools (2; 6; 9) (W);  Policies to accommodate persons with ADHD School-Special education program (3; 6) (W);  Individualised education plans (3) (S; U; W);  Community organisations-support groups ADA (3) (U);  Symptom awareness (4; 6) (U);  Health systems: self-management support (5) (U);  Access to care (8) (S; U);  Health care providers-availability (8) (S; W);  Primary care and psychiatric providers (8) (W);  Routine follow-up (13) (S);  Therapists funding:-insurance coverage (20) (W) | Secondary care | CYP with ADHD | ADHD | LD& Autism | US |
| Platt 2018  (163) | On-site IC: interprofessional collaboration and communication (1) (W);  Clear protocols facilitating intervention screening (6) (S);  Institutionalizing interprofessional collaboration via higher levels of integration (with co-located mental health providers systematically incorporated into routine medical care and PCPs/co-located providers developing joint treatment plans) (14) (S; W) | Primary care | CYP with MH problems | MH | Mental health | US |
| Ramos-Gomez 2021  (164) | Collaborative practice between dental, medical, and other pediatric primary care provider (2) (W);  Innovative systems approach through greater prevention and disease management by conducting risk assessments and obtaining a greater awareness in oral health literacy (4) (S);  Infrastructure development (4) (S);  Staff training (7) (W);  Cultural competency (18) (S) | Dentistry care; primary care | General | Dentistry | Dentistry | US |
| Ranade-Kharkar 2017  (165) | Tracking progress (1; 6; 9) (S);  Collaborative planning (2) (S);  Interpersonal communication and information transfer among entities (11) (S; W);  Parents acting as active agents so called “family partners” (2; 3; 5) (U);  IT tools: supporting situation understanding (10) (S);  Interpersonal communication and information transfer among entities (11) (S; W);  Care networking (15) (W);  Participants used workarounds to adapt and overcome limitations in their information environment (18) (U) | Multiple care providers | CYP with complex needs | Complex needs | Long-term complex medical conditions | US |
| Roesler 2019  (166) | Multidisciplinary treatment team (1; 2) (W);  Vertical integration (with some horizontal aspects) (2) (S);  Staff/family partnership (2; 18) (U; W);  Family-based treatment (3) (U);  One treatment environment (‘one stop shop’) (8; 14) (S; U) | Day treatment, family treatment | CYP with complex needs | Complex needs, paediatric illness associated with psychiatric comorbidity | Long-term complex medical conditions | US |
| Romba and Ballard 2020  (167) | Tracking progress (1; 6; 9; 13) (S);  Familiar setting (3) (U);  Evidence-based, patient-centred care (3) (U);  Early intervention (4) (S);  Training and guidance (7) (W);  Electronic records (10) (S);  Co-located care (14) (S);  The need for a ‘champion’ (17; 23) (W) | Primary care | CYP with MH problems | Mental and behavioural health | Mental health | US |
| Rousseau 2017  (168) | Co-location in community health centres (1; 8; 14) (S);  Common care culture (1; 14) (W)  Shared leadership (1; 17) (W);  Direct (with the patient & family) or indirect (case discussion) consultations (11) (U);  Meaningful engagement of staff (14) (W);  Effective team working (14) (W); | Primary care | CYP with MH problems | MH | Mental health | Canada |
| Saia 2020  (169) | Mutual trust (1) (U; W);  Non-hierarchical relations (1; 11; 21) (W);  Active and on-going partnerships (1) (W);  Common understanding, and the “co-creation” effect of problem mapping (1) (W);  Ensuring the full participation of youth and their parents/caregivers (2; 3) (U);  Child-friendly counselling context (2; 3) (U);  Unconditional support and empowerment of the family (5) (U);  Child-appropriate language (8; 18) (U);  Supportive management (11) (W);  Goal setting and problem-solving (12) (W);  Reminders and close contact with case manager appreciated (12; 16) (W);  Use of up-to-date communication devices and tools valued (13) (W);  Provide resources and information (13) (S);  Case manager is the link among networks (16) (W);  Case manager is the single point of contact (16) (W);  The importance of the case manager’s role (16) (W);  Feeling safe and trusted (23) (U) | Social rehabilitation services | CYP with complex needs | Complex needs; Offenders | Long-term complex medical conditions | Estonia |
| Sale 2021  (170) | Screening (4) (S; U);  Consistent and direct communication (5) (U; W);  Trusting peer training (7) (W);  Care coordination (12) (W);  Rapid follow-up to care (13) (S);  Continuity-of-care approach (13) (S);  Care networking (15) (W) | Primary care | CYP with complex needs | Youth Suicidality, MH | Mental health | US |
| Sarkadi 2021  (171) | Electronic health records (1; 10) (S);  Multidisciplinary team: psychologist, physician, speech therapist, physiotherapist, special educator (1; 2) (W);  Incoming referral (4) (U);  Routine follow-up (13) (S) | Primary care | CYP with complex needs; early years | MH (Multiple neurodevelopmental problems (Attention Deficit Hyperactivity Disorder (ADHD), Developmental Coordination Disorder (DCD), autism spectrum disorder (ASD), developmental language disorder, conduct disorder, and learning impairment) | long-term complex medical conditions,; LD& Autism | Sweden |
| Satherley 2021  (172) | Supporting All Sectors to Take on a Meaningful Role in Child Health (1) (U; W);  Families experienced coordination across health, social, and education systems (1; 2; 3) (U);  Looking after the whole person (1) (S; U);  A multidisciplinary team (2) (W);  Beyond the key professional preference for conventional care that families experienced coordination across health, social, and education systems (1; 2) (U; W);  Connecting Services Together (2) (S);  Importance of Formal and Informal Contacts (2) (U; W);  Feeling heard and receiving contextualized support (3) (U);  Material resources that flow (or not) from being heard that professionals involved children and caregivers in treatment (3; 20) (U);  Respect for young person’s autonomy (3) (U);  Trust is built on the recognition of professional learning and enhanced through physical presence (3) (U; W);  Professionals involved children and caregivers in treatment (3; 18; 21) (U; W);  The key health-worker understood the health needs of the family in context (6; 16) (U; W);  Each theme also had sub-themes that the key health-worker understood the health needs of the family in context (6) (W);  Negotiating Point of Independence that holistic care that supported the family unit was provided (11) (U);  Communication in the Context of Uncertainty (11) (U; W);  Holistic care that supported the family unit was provided (11) (S; U);  Importance of relationships with key professional who coordinates care (16) (W);  Initial distrust of intrusion (19) (U);  Time facilitates personal disclosures (19) (U);  Challenges of Working Alongside a Stretched Workforce (25) (W) | Multiple health providers, schools | CYP with complex needs, CYP with long-term conditions, early years focus | Asthma; Eczema; Epilepsy; Constipation | Long-term complex medical conditions | UK |
| Schurman and Friesen 2021  (173) | Each professional contributes to the care of the patient or an interdisciplinary program where professionals also directly interact with shared decisions taking into account the numerous factors contributing to outcomes (1) (W);  Electronic medical record (1; 10) (S);  Young adult clinic (3) (U);  Acknowledging to a patient that when we take on the responsibility to care for a patient with IBD (3) (U);  Accepting the responsibility to identify and treat all relevant factors which can affect outcome (9) (W);  Need for a psychologist as an integral member of the healthcare team; electronic medical record (16) (W);  Emphasis on finances that need to be assessed as total inflow minus total outflow (20) (S) | Primary care | CYP with inflammatory bowel disease | Inflammatory bowel disease | Inflammatory bowel disease | US |
| Scott 2021  (174) | Need-driven care (6) (S);  Provision of support and resources; realistic expectations (6) (S; W);  Trauma-informed care (7; 19) (S);  Evidenced-based care (7; 9) (S) | Primary care | CYP with MH problems, early years focus | MH (children with disruptive behaviour disorders and/or children and parents who have established a cycle of negative relational interaction, placing them on a trajectory toward an externalized behavioural diagnosis) | Mental Health | US |
| Sengupta 2017  (175) | Coordinated care (information exchange) (1; 6) (S; W);  Sharing common electronic health records (1; 10) (S: W);  Screening of risks (4; 9) (S; W);  Prevention of treatment (4) (S);  Reinforce anticipatory guidance (4; 6) (S);  Fast-track appointments (8) (S); | Primary care | General; Early years | Dentistry | Dentistry | US |
| Shahidullah 2018  (176) | Coordinated/telephonic models to fully integrated models (i.e. behavioral health care delivered in clinic with significant collaboration and coordination of treatment plan between behavioural health clinician and other sites) (1; 12; 13; 15) (S);  Postdoctoral fellows in clinical/school psychology serving as collaborative care therapists with a local community member serving as a community health partner (partnership with community through a local citizen) (2) (W);  Care managers to facilitate IC Foreign language specialists (Spanish –speaking SWs) (3; 16; 24) (W);  Staff training (7) (W) | Primary care | CYP with ADHD | MH, Attention- deficit /hyperactivity disorder (ADHD) | Mental health; LD& Autism | US |
| Shaligram 2022  (177) | shared care (1) (S; W); collaborative care (1) (S; W); coordinated care (1) (S; W); stepped care (2) (S; U); co-located care (14) (S) | Primary Care | CYP with Mental Health | CYP with Mental Health | Mental Health | UK; Spain; US; Canada; Brazil; Uruguay; Kenya; Micronesia |
| Shannon 2023  (178) | provisioning education programs for nursing staff (7) (S; W); implementing nurse-led BH response teams to assist staff with psychiatric patient escalations (16) (S; W); Increased hospital security (19) (S); constant observer care workforce (19) (S) | Primary care | CYP with Mental Health | CYP with Mental Health | Behavioural health, Mental Health | US |
| Shippee 2018  (179) | Emphasising patient-reported outcomes (3; 6) (W);  Registries and systematic screening (4) (S);  Evidence-based protocols (9) (S);  Collaboration: Team driven (15) (W) | Primary care, psychiatric care | CYP with MH problems | Depression without a diagnosis of bipolar disorder, MH | Mental health | US |
| Smith 2016  (180) | Electronic health records (1; 10) (S);  Family-centred care coordination (3; 5) (U);  Access to specialised care (8) (S; U);  Comprehensive assessments (9) (S);  Feeling safe and trusted (19) (U; W);  Onsite mental health professional (22) (W) | Primary care | General; CYP with MH problems; Early years focus | General; MH | Mental health; General | US |
| Stadnick 2020  (181) | Cross-system communication (11) (S; W);  Efficient communication between primary and mental health care providers (2; 11; 15) (W);  Importance of comprehensive screening and successful mental health linkage (2; 4; 6) (S);  Patient-centred care (3) (U);  Timely identification of mental health needs (4) (S);  Dedicated case management or navigation to help families access and engage with mental health care (5; 16) (W) | Primary care | CYP with MH problems; CYP with ASD | ASD, MH | Mental health; LD& Autism; | US |
| Stadnick 2022a  (182) | Shared responsibility (1) (W);  Evaluation (9) (S) | Primary care | CYP with MH problems; CYP with ASD | ASD, MH | Mental health; LD& Autism | US |
| Stadnick 2022b  (183) | Mental health screening (9) (S; U) | Primary Care | CYP with ASD | CYP with ASD | Mental health  LD& Autism | US |
| Suen 2021  (184) | Interdisciplinary team (1) (W);  Prolonged visits (3) (S);  Care delivery within and outside clinic (6; 8) (S);  Care coordination (12) (S; W) | Community-based paediatric practices | CYP with MH problems; CYP with ASD | ASD, MH | Mental health; LD& Autism | US |
| Sultan 2018  (185) | Shared care or collaborative care (1) (S; W);  Multidisciplinary collaboration and enhancement of primary care providers’ capacity (2) (W);  Joint responsibility (2) (W);  Liaise between primary care providers and a decision support panel (2) (W);  Multidisciplinary collaboration (4) (W);  Evaluation of patients’ psychological progress (4; 9) (S; U);  Psychoeducational and skills training for families-education to parents (5) (U);  Triple P training (7) (S);  Expedited access to outpatient psychiatric consultation (8) (S; U);  Phone consultations (8) (S; U; W);  Motivational interviewing (11) (W) | Primary care; Mental health providers | CYP with ADHD | ADHD, MH | Mental health; LD& Autism | US |
| Talbott 2021  (186) | Integrated electronic health record (1; 10) (S);  Strong collaboration (1) (S; W);  Parents have freedom to select best treatment for their child multi-informant assessment (3) (U);  Use of data from multiple informants (4; 6) (S; W);  Implementation science (9) (S);  Electronic survey (feedback from parents and teachers) (10; 18) (U; W);  E-mail-based platform for sharing data between parents and teachers (10; 11) (U; W);  Communication (11) (W);  Web service design to facilitate communication (11) (S);  Teamwork -> effective teamwork have been identified by researchers in both health care and education (15) (W);  Effective leadership (17) (W);  Language (24) (W); | Education and Health Care Settings | CYP with ADHD; CYP with MH problems | ADHD, MH | Mental health; LD& Autism | US |
| Tennant 2020  (187) | Knowledge transfer activities and shared learning among collaborators (1) (W);  Development and implementation of shared assessment tools and referral criteria (1; 9) (S);  Leveraging other family, social, and organisational relationships (2; 18) (U);  Building trust (3) (U; W);  Meeting clients on their own terms (3) (U);  Client empowerment (3) (U);  Flexible service delivery (3) (S);  Informal knowledge transfer (7) (W);  Co-design and co-production of the initiative in partnership with families and service partners (5) (U);  Implementation of family assessment and engagement tools that can be used over the long-term to monitor the health and wellbeing of family members (9; 18) (U);  Co-location of multidisciplinary and/or interagency staff and cultivating faith in positive change among staff (14) (W); | Community-based care | CYP with complex health needs | Complex health and social care need | long-term complex medical conditions | Australia |
| Toros 2021  (188) | Information exchange between professionals within interprofessional groups (1) (W);  Settings, levels of care, and sectors (1; 2) (S);  Stronger connections and partnerships: forming professional partnerships (2) (W);  Stakeholders working collaboratively across disciplines (2) (W);  Need for shared vision across multiple professional disciplines (2) (W);  Comprehensive support system for nursery schools (2; 3) (S);  Early assessment and intervention to prevent crises (4) (S; W);  CPS monitoring on sight (4; 9; 13) (S);  Regular practice-team meeting to problem solve and assess progress; Training of education staff (informed knowledge of CPS roles) and joint training (7) (W) | Primary care | Vulnerable Groups; Early years | General | General | Estonia |
| Ufer 2018  (189) | Client empowerment (3) (U; W);  Coordination (15) (W);  Key manager (16) (W); | Medical Home | CYP with SHCN | Special Health Care Needs | Special Health Care Needs | US |
| Vaggers and Anderson 2021  (190) | Thinking systemically (2; 7; 12; 15) (W);  Build and rebuild relationships (2) (W);  Alternative approaches to solving complex problems (8) (U);  Self-actualization (15) (U);  Collaborative leadership: building shared meaning (17) (W) | Primary care | General | General | General | UK |
| van Dongen 2020  (191) | Strength-based and culturally competent, accessible language (1) (U);  Participation of adolescents and parents (3) (U);  Personalised, family-centred approach (5) (U);  A solution-focused approach (6) (S);  A neutral chairperson (16) (U);  The presence of specialist practitioners (22) (W) | Primary care; Secondary care | CYP with complex needs | Multiple complex needs; MH | long-term complex medical conditions; Mental health | Belgium |
| Versloot 2021  (192) | Population-centred care (3) (U);  Systematic screening and monitoring with treatment to target (4; 6; 9) (S);  Collaborative Inter-professional care that is patient-centred (5) (W);  Evidence-based protocols (9) (S);  Measurement based (9) (S);  Teams and teamwork (15) (W) | Paediatric mental health care, primary care | CYP with MH problems; CYP with diabetes | Diabetes; MH | Diabetes; mental health | US |
| Walter 2021  (193) | Integration of services (physical and mental health support/services) (2) (S);  Accessibility/quality of appropriate care for behavioural health (mental health/emotional wellbeing) needs (8) (S) | Primary care | CYP with MH problems | MH | Mental health | US |
| Ward 2021  (194) | Effective communication (1) (W);  Shared decision-making (2) (W);  The time needed to build professional relationships (2) (W);  Patient-centred focus (3) (U);  Better understanding of roles and responsibilities (8) (W) | Paediatric psychology service | CYP with MH problems; Early years focus | MH | Mental health | US |
| Wolfe 2020  (195) | Electronic health records (1; 10) (S);  Shared care records between the secondary and primary care providers (2) (W);  Education for caregivers (including psychoeducational) (7) (U); | Secondary care; Primary care providers | CYP with asthma | Asthma | Asthma | US |
| Wolfe 2023  (196) | self-management support and health promotion (3) (U); resilience building and mental health first aid (3) (U); health checks (9) (U); electronic decision support (10) (S); a primary care hotline (10) (S) | Primary Care | children ≥16 | asthma, eczema, constipation | asthma, eczema, constipation | UK |
| Yonek 2020  (197) | Measurement-based care (4) (S);  Delivery of evidence-based mental health services (4) (S);  Population-based care (6) (S); | Paediatric Integrated Mental Health Care | CYP with MH problems | MH disorders | Mental health | US |
| Yu 2017  (198) | Coordination (e.g. consultation between physical and behavioural health care providers) (1) (W);  Collaboration (e.g. the primary care practice has its own integrated behavioural health provider who collaborates with practice staff to serve all patients) (2) (W);  Vertical integration (2) (S);  “Back up” of psychiatrists services (4) (W);  Progress monitoring (4; 6) (W);  Use of individual financial records (6) (U);  Training of specialists (7) (W);  Service supervision (13) (W);  Co-location (e.g. a separate behavioural health provider receives a facilitated referral to treat a patient from a partnered primary care practice down the hall) (14) (S); | Primary care | CYP with MH problems | Paediatric Behaviour Disorders, MH, attention-deficit/hyperactivity disorder | Mental health; LD& Autism | UK |
| Zanello 2017  (199) | Development of care coordination measurement instrument (identity record and encounter record) (1; 12) (W);  Family paediatrician as a key figure coordinating care (17) (U; W); | Primary care | Early years focus; CYP with SHCN | Special health care needs; newborns | Special Health Care Needs | Italy |
| Zima 2018  (200) | Consult on the scientific evidence for care processes (1) (S; W);  Strengthen foundation-clinic-academic partnership (2) (W);  Create work flow analyses prior to implementation (2; 6; 12) (S);  Advise on measurement-based care (6; 9) (S);  Integrate evaluation planning during care model development (6) (S);  Incorporating evaluation process in the mental health delivery processes: shared learning, identifying common care processes (9) (S); | Primary and mental health care services | Vulnerable groups; CYP with MH problems; early years focus | MH | Mental health | US |

**Table S3: List of the 47 studies excluded from this review at full paper screening stage, and reasons for their exclusion.**

| 1 | Alley DE, Ashford NC, Gavin AM. Payment innovations to drive improvements in pediatric care—the integrated care for kids model*. JAMA Pediatrics*. 2019; 173(8): 717-718. DOI: https://doi.org/10.1001/jamapediatrics.2019.1703. | **Publication Type**  A paper overview |
| --- | --- | --- |
| 2 | Banerjee J, Aloysius A, Mitchell K, Silva I, Rallis D, Godambe SV, et al. Improving infant outcomes through implementation of a family integrated care bundle including a parent supporting mobile application. *Archives of Disease in Childhood: Fetal and Neonatal Edition.*2020;105(2):172-177. DOI: https://doi.org/10.1136/archdischild-2018-316435. | **Population**  Not our population focus (CYP). |
| 3 | Benjumea-Bedoya D, Villegas Arbeláez E, Martínez-Peñaloza D, Beltrán-Arroyave CP, Restrepo Gouzy AV, Marín D, et al. Implementation of an integrated care strategy for child contacts of tuberculosis patients: a quasi-experimental study protocol. BMC Pediatrics. 2023; 23(1): 28. DOI: https://doi.org/10.1186/s12887-022-03798-x. | **Country**  Study was conducted in Columbia (UMIC) |
| 4 | Benzies KM, Aziz K, Shah V, Faris P, Isaranuwatchai W, Scotland J, et al. Effectiveness of Alberta Family Integrated Care on infant length of stay in level II neonatal intensive care units: a cluster randomized controlled trial. *BMC Pediatrics.* 2020; 20(1): 535. DOI: https://doi.org/10.1186/s12887-020-02438-6. | **Population**  Not our population focus (CYP). |
| 5 | Bert F, Camussi E, Gili R, Corsi D, Rossello P, Scarmozzino A, et al. Transitional care: A new model of care from young age to adulthood. *Health Policy.* 2020;124(10):1121-1128. DOI: https://doi.org/10.1016/j.healthpol.2020.08.002. | **ICS or ICS component evidence**  Not an integrated care model focus. |
| 6 | Boesveld IC, Bruijnzeels MA, Hitzert M, Hermus MAA, van der Pal-de Bruin KM, van den Akker-van Marle ME, et al. Typology of birth centres in the Netherlands using the Rainbow model of integrated care: results of the Dutch Birth Centre Study. *BMC Health Services Reseach*. 2017; 17(1): 426. DOI: https://doi.org/10.1186/s12913-017-2350-9. | **Population**  Not our population focus (CYP). |
| 7 | Borer MS, McDaniel SH. Child Psychiatrists and Psychologists: Enhanced Collaboration in Primary Care. *Child and Adolescent Psychiatric Clinics of North America*. 2021; 30(4): 809-826. DOI: https://doi.org/10.1016/j.chc.2021.06.007 | **Pubication Type** Commentary. |
| 8 | Brenner M, O'Shea M, Larkin PJ, Kamionka SL, Berry J, Hiscock H, et al. Exploring Integration of Care for Children Living with Complex Care Needs across the European Union and European Economic Area. *International Journal of Integrated Care.* 2017; 17(2): 1-5. DOI: https://doi.org/10.5334/ijic.2544. | **Pubication Type**  Brief report. |
| 9 | Cohen E, Coller RJ. Evaluating Integrated Care for Children: A Clarion Call or a Call for Clarity? *Pediatrics.* 2020; 145(1): e20193282. DOI: https://doi.org/10.1542/peds.2019-3282. | **Pubication Type**  Commentary. |
| 10 | Costello AG, Nugent BD, Conover N, Moore A, Dempsey K, Tersak JM. Shared Care of Childhood Cancer Survivors: A Telemedicine Feasibility Study. *Journal of Adolescent and Young Adult Oncology.* 2017; 6(4): 535-541. DOI: https://doi.org/10.1089/jayao.2017.0013. | **Population**  Age group: 18 years old or older. |
| 11 | Dhar V. Integrated care pathways for controlling early childhood caries. *Pediatric Dentistry*. 2020; 42(1): 10-11. Available at: https://pubmed.ncbi.nlm.nih.gov/32075703/ (accessed 28th November 2023, no doi). | **Pubication Type**  Letter to the editor. |
| 12 | Eilander MMA, van Mil MMA, Koetsier LW, Seidell J, Halberstadt. Preferences on how to measure and discuss health related quality of life within integrated care for children with obesity. *Journal of Patient Reported Outcomes*. 2021; 5: 106. DOI: https://doi.org/10.1186/s41687-021-00381-3. | **ICS or ICS component evidence**  Study uses a method to collect data that informs development and implementation of an ICS but does not report ICS components. |
| 13 | Feehan K, Kehinde F, Sachs K, Mossabeb R, Berhane Z, Pachter LM, et al. Development of a Multidisciplinary Medical Home Program for NICU Graduates. *Maternal and Child Health Journal.* 2020; 24(1): 11-21. DOI: https://doi.org/10.1007/s10995-019-02818-0. | **Population**  Not our population focus (CYP). |
| 14 | Fontanella CA, Warner LA, Steelesmith DL, Brock G, Bridge JA, Campo JV. Association of Timely Outpatient Mental Health Services for Youths After Psychiatric Hospitalization With Risk of Death by Suicide. *JAMA Network Open*. 2020; 3(8): e2012887. DOI: https://doi.org/10.1001/jamanetworkopen.2020.12887. | **ICS or ICS component evidence**  Not an integrated care service/no evidence of integrated care. |
| 15 | Franck LS, O'Brien K. The evolution of family-centered care: From supporting parent-delivered interventions to a model of family integrated care. *Birth Defects Research*. 2019; 111(15): 1044-1059. DOI: https://doi.org/10.1002/bdr2.1521. | **Population**  Integrated care within a neo-natal care unit only, not a wider system. |
| 16 | Franck LS, Waddington C, O'Brien K. Family Integrated Care for Preterm Infants. *Critical Care Nursing Clinics of North America*. 2020; 32(2): 149-165. DOI: https://doi.org/10.1016/j.cnc.2020.01.001 | **Population**  Not our population focus (CYP). |
| 17 | Fritz GK. Integrated care promotes professional communication. *The Brown University Child and Adolescent Behavior Letter*. 2016; 32(10): 8-8. Available at: <https://onlinelibrary.wiley.com/doi/10.1002/cbl.30160> (accessed 28th November 2023, no doi) | **Publication**  Editor’s comment. |
| 18 | Ghosh S, Yasmin M, Sen K, Goswami S, Das TC, Swar SC, et al. Integrated Care for Type 1 Diabetes: The West Bengal Model. Indian Journal of Endocrinology and Metabolism. 2023; 27(5): 398-403. DOI: https://doi.org/10.4103/ijem.ijem_124_23. | **Country**  Study was conducted in India (LMIC) |
| 19 | Green JG, Comer JS, Donaldson AR, Elkins RM, Nadeau MS, Reid G, et al. School Functioning and Use of School-Based Accommodations by Treatment-Seeking Anxious Children. *Journal of Emotional and Behavioral Disorders*. 2017; 25(4): 220-232. DOI: https://doi.org/10.1177/1063426616664328 | **Population**  Not our population focus (CYP) nor the outcomes we are particularly interested in. |
| 20 | Grimes K, Webster C, Coffey S, Hagan G. Integrated care for children: a shared training model. *Medical Education*. 2017; 51(5): 553. DOI: https://doi.org/10.1111/medu.13308. | **Publication Type**  (Commentary) |
| 21 | Hayward LM, Meleis W, Mahanna J, Ventura SH. Interprofessional Collaboration Among Physical Therapy, Speech-Language Pathology, and Engineering Faculty and Students to Address Global Pediatric Rehabilitation Needs: A Case Report. *Journal of Physical Therapy Education.* 2016; 30(4): 24-34. DOI: : https://doi.org/10.1097/00001416-201630040-00005 | **Country**  Case study focusing on Ecuador children. |
| 22 | Hilty DM, Sunderji N, Suo S, Chan S, McCarron RM. Telepsychiatry and other technologies for integrated care: evidence base, best practice models and competencies. *International Review of Psychiatry.* 2018; 30(6): 292-309. DOI: https://doi.org/10.1080/09540261.2019.1571483. | **Population**  Not focusing specifically on children, looks at all population |
| 23 | Koetsier LW, van Mil MMA, Eilander MMA, van den Eynde E, Baan CA, Seidell JC, et al. Conducting a psychosocial and lifestyle assessment as part of an integrated care approach for childhood obesity: experiences, needs and wishes of Dutch healthcare professionals. *BMC Health Services Research.* 2021; 21: 611. DOI: https://doi.org/10.1186/s12913-021-06635-6 | **Publication**  ICS measurement tool development paper |
| 24 | LaVille Thoren K, Vista-Wayne J. The collaborative care model: Improving access to children's mental health care. *Journal of Child and Adolescent Psychiatric Nursing.* 2021; 34(2): 83-87. DOI: https://doi.org/10.1111/jcap.12309. | **ICS or ICS component evidence**  Article explores the experiences of using a certain type of assessment within an ICS. Does not describe the ICS components. |
| 25 | Lee NC, Chien YH, Hwu WL. Integrated care for Down syndrome. *Congenital Anomalies (Kyoto).* 2016; 56(3): 104-6. DOI: https://doi.org/10.1111/cga.12159. | **ICS or ICS component evidence**  Mini review, Taiwan, the need for ICS is highlighted |
| 26 | LeRoux K, Piatak J, Romzek B, Johnston J. Informal Accountability in Children’s Service Networks: The Role of Frontline Workers. *Human Service Organizations: Management, Leadership & Governance*. 2019; 43: 3, 188-204. DOI: https://doi.org/10.1080/23303131.2019.1637804 | **ICS or ICS component evidence**  Provides an overview on the current situation in children’s services without a discussion about any IC initiative |
| 27 | Lindner S, Kubitschke L, Lionis C, Anastasaki M, Kirchmayer U, Giacomini S, et al. Can Integrated Care Help in Meeting the Challenges Posed on Our Health Care Systems by COVID-19? Some Preliminary Lessons Learned from the European VIGOUR Project. *International Journal of Integrated Care*. 2020; 20(4): 4. DOI: https://doi.org/10.5334/ijic.5596. | **Population**  This paper highlights challenges arising in the care for older and vulnerable populations. |
| 28 | Lorenzo A, Robinson L, DeJong SM. Teaching Pediatric Integrated Care: A Review of Best Practices for Structuring, Implementing, and Evaluating the Educational Experience for Trainees (abstract). *Journal of the American Academy of Child & Adolescent Psychiatry (The Scientific Proceedings of the 65th Annual Meeting)*. 2018; 57 Supplement 10: S46-S46. DOI: https://doi.org/10.1016/j.jaac.2018.07.197 | **Publication**  An overview of a workshop. |
| 29 | Marcu MI, Knapp CA, Brown D, Madden VL, Wang H. Assessing the impact of an integrated care system on the healthcare expenditures of children with special healthcare needs. *American Journal of Managed Care.* 2016; 22(4): 272-280. (could not find DOI, accessed at: [AJMC_04_2016_Marcu%20(final).pdf (sanity.io)](https://cdn.sanity.io/files/0vv8moc6/ajmc/139848b5ddd23700ecb7e70084d08039eb29cd64.pdf/AJMC_04_2016_Marcu%2520(final).pdf) 28^th^ November 2023). | **ICS or ICS component evidence**  Paper focuses on expenditures based on ICS integration |
| 30 | Martinussen M, Kaiser S, Adolfsen F, Patras J, Richardsen AM. Reorganisation of healthcare services for children and families: Improving collaboration, service quality, and worker well-being. *Journal of Interprofessional Care*. 2017; 31(4): 487-496. DOI: https://doi.org/10.1080/13561820.2017.1316249. | **ICS or ICS component evidence**  Focuses on care workers |
| 31 | McKeown A, Cliffe C, Arora A, Griffin A. Ethical challenges of integration across primary and secondary care: a qualitative and normative analysis. *BMC Medical Ethics.* 2019; 20: 42. DOI: https://doi.org/10.1186/s12910-019-0386-6. | **ICS or ICS component evidence**  Explores ethical issues associated with integrated care rather than components – not outcome of interest |
| 32 | Moore SA, Cooper JM, Malloy J, Lyon AR. Core Components and Implementation Determinants of Multilevel Service Delivery Frameworks Across Child Mental Health Service Settings. Administration and Policy in Mental Health and Mental Health Services Research. 2024; 51(2): 172-195. DOI: https:/doi.org/10.1007/s10488-023-01320-8. | **ICS or ICS component evidence**  Conceptual review (cited in the introduction to this paper) which did not identify core components but instead identified commonalities and variation among components across three exemplar frameworks. |
| 33 | Pomare C, Long JC, Ellis LA, Churruca K, Braithwaite J. Interprofessional collaboration in mental health settings: a social network analysis. *Journal of Interprofessional Care.* 2019; 33(5): 497-503. DOI: https://doi.org/10.1080/13561820.2018.1544550. | **ICS or ICS component evidence**  Measurement of one component of a small scale ICS (isolated pathway) - not outcome of interest |
| 34 | Pygott N, Hartley A, Seregni F, Ford TJ, Goodyer IM, Necula A, et al. Research Review: Integrated healthcare for children and young people in secondary/tertiary care - a systematic review. Journal of Child Psychology and Psychiatry. 2023; 64(9): 1264-1279. DOI: https://doi.org/10.1111/jcpp.13786. | **ICS or ICS component evidence**  Comprehensive systematic review (cited in the introduction to this paper) which synthesised and evaluated the evidence  for effectiveness and cost-effectiveness of integrated care for CYP. It did not describe the components of individual models per se. |
| 35 | Rickerby ML, DerMarderosian D, Nassau J, Houck C. Family-Based Integrated Care (FBIC) in a Partial Hospital Program for Complex Pediatric Illness: Fostering Shifts in Family Illness Beliefs and Relationships. *Child and Adolescent Psychiatric Clinics of North America.* 2017; 26(4): 733-759. DOI: https://doi.org/10.1016/j.chc.2017.06.006. | **Publication Type**  An overview of the paper |
| 36 | Ross A, Arnold J, Gormley A, Locke S, Shanske S, Tardiff C. Care coordination in pediatric health care settings: the critical role of social work, Social Work in Health Care. 2019; 58(1): 1-13. DOI: https://doi.org/10.1080/00981389.2018.1514352 | **ICS or ICS component evidence**  Explores time spent on tasks for one type of health care worker - not outcome of interest |
| 37 | Saggu H, Jones C, Lewis A, Baynam G. mEDUrare: Supporting Integrated Care for Rare Diseases by Better Connecting Health and Education Through Policy. Yale Journal of Biology and Medicine. 2021; 94(4): 693-702. Available at: [yjbm_94_4_693.pdf (nih.gov)](https://www.ncbi.nlm.nih.gov/pmc/articles/PMC8686785/pdf/yjbm_94_4_693.pdf) (no doi found, accessed 28th November 2023). | **ICS or ICS component evidence**  A review of existing national and state health and education policies, to inform the advancement of health and education sector integration in rare disease care provision NOT a review of ICSs |
| 38 | Satherley R, Green J, Sevdalis N, Newham JJ, Elsherbiny M, Forman J, et al. The Children and Young People’s Health Partnership Evelina London Model of Care: process evaluation protocol. BMJ Open. 2019; 9: e027302. DOI: https://doi.org/10.1136/bmjopen-2018-027302 | **Publication Type**  Protocol |
| 39 | Schweitzer J, Bird A, Bowers H, Carr-Lee N, Gibney J, Schellinger K, et al. Developing an innovative pediatric integrated mental health care program: interdisciplinary team successes and challenges. Front Psychiatry. 2023 Nov 16;14:1252037. doi: 10.3389/fpsyt.2023.1252037. | **ICS or ICS component evidence**  Describes the development, challenges and success story of the Primary Care Mental Health Integration (PCMHI) program, but not the systematic identification of components. |
| 40 | Stadnick NA, Brookman-Frazee L, Mandell DS, Kuelbs CL, Coleman KJ, Sahms T, et al. A mixed methods study to adapt and implement integrated mental healthcare for children with autism spectrum disorder. *Pilot and Feasibility Studies.* 2019; 5: 51. DOI: https://doi.org/10.1186/s40814-019-0434-5. | **Publication Type**  Protocol |
| 41 | Sydney Hsiung K, Hart J, Kelleher KJ, Kolko D. 2.40 The role of stressful climate and provider perceptions of integrated behavioral health services in pediatric primary care (abstract)). *Journal of the American Academy of Child & Adolescent Psychiatry (The Scientific Proceedings of the 66th Annual Meeting)*. 2019; 58 Supplement 10: S183‐S184. DOI: https://doi.org/10.1016/j.jaac.2019.08.133 | **Publication**  Conference paper. |
| 42 | Thompson H, Faig W, Gupta N, Lahey R, Golden R, Pollack M, et al. Collaborative Care for Depression of Adults and Adolescents: Measuring the Effectiveness of Screening and Treatment Uptake. *Psychiatric Services*. 2019; 70(7): 604-607.  DOI: https://doi.org/10.1176/appi.ps.201800257 | **Publication Type**  Brief report |
| 43 | Todd K, Eastwood JG, Fotheringham P, Salinas-Perez JA, Salvador-Carulla L. Using Geospatial Analysis to Inform Development of a Place-Based Integrated Care Initiative: The Healthy Homes and Neighbourhoods Experience. *International Journal of Integrated Care.* 2021; 21(2): 23. DOI: https://doi.org/10.5334/ijic.5430. | **ICS or ICS component evidence** |
| 44 | Versloot J, Saab H, Minotti SC, Ali A, Ma J, Reid RJ, et al. An Integrated Care Model to Support Adolescents With Diabetes-related Quality-of-life Concerns: An Intervention Study. Canadian Journal of Diabetes. 2023; 47(1): 3-10. DOI: https:/doi.org/10.1016/j.jcjd.2022.05.004. | **ICS or ICS component evidence**  This paper reports an outcome evaluation of an integrated care model which is already an included study (Versloot 2021). |
| 45 | Vlahou CH, Petrovski G, Korayem M, Al Khalaf F, El Awwa A, Mahmood OM, et al. Outpatient clinic-wide psychological screening for children and adolescents with type 1 diabetes in Qatar: An initiative for integrative healthcare in the Gulf region. *Pediatric Diabetes*. 2021; 22(4): 667-674. DOI: https://doi.org/10.1111/pedi.13200. | **ICS or ICS component evidence**  Not an implementation or evaluation of an ICS for CYP |
| 46 | Waldo JA, Henderson C, Dauber S, Hogue A. DIfferences in Treatment Trajectories Between Two Profiles of Adolescent Systems Involvement. *Child and Youth Services Review*. 2021; 121: 105811. DOI: https://doi.org/10.1016/j.childyouth.2020.105811. | **ICS or ICS component evidence**  Duplicate – pre-print identified in original search and already data extracted |
| 47 | Zimmermann M, O'Donohue W, Zepeda M, Woodley A. Examining Caretaker Attitudes Towards Primary Prevention of Pediatric Behavioral Health Problems in Integrated Care. *Journal of Behavioral Health Services & Research.* 2021; 48(1): 120-132. doi: 10.1007/s11414-020-09720-6. Erratum in: *Journal of Behavioral Health Services & Research.* 2020 Sep 13. | **ICS or ICS component evidence**  Article explores compliance/completion rates of an ICS training course. Does not describe the ICS or its components. |

**Table S4: List of the 25 Component Themes and their coded components of integration, as identified from the 170 included studies.**

| **Component Theme (n=25)** | **Components of integration, identified from the included studies, which mapped onto this Component Theme. Each component is coded for the Component Theme(s) it mapped onto and its target(s) of impact** |
| --- | --- |
| **.One:**  **Shared Professional Responsibility and Practices (n=179)** | 1. Care coordination between patients and family-centred team (1) (U; W); 2. Shared decision making and responsibility among providers (1) (W); 3. Support through networking, education and partnership empowerment (1) (S; U; W); 4. Active care (1, 18) (S; W); 5. Shared electronic record (1) (S); 6. Care coordination between patients and family-centred team (1) (U; W); 7. Clear division of responsibilities (1) (W); 8. Support through networking, education and partnership (1; 16) (W); 9. Coordinated pathways to specialist care and legal and governance structures (1) (S); 10. Coordinated pathway to specialist care legal and governance structures (1) (S); 11. Co-location in community health centres (1; 8; 14) (S); 12. Care coordination between patients and family-centred team (1) (U; W); 13. Co-location of multidisciplinary and/or interagency staff (1, 14) (S); 14. Coordinated care (1) (S; W); 15. Co-location (e.g., a separate behavioural health provider receives a facilitated referral to treat a patient from a partnered primary care practice down the hall) (1; 14) (S); 16. Partnerships with families (1; 2) (U; W); 17. Information exchange and individualised care plans (decentralised decision-making) (1) (S); 18. Addressing the child’s and family’s needs (1; 3) (U; W); 19. Sharing the care of the patient and patient/family inclusion in care planning and delivery (1) (U); 20. Coordination of providers with defined roles (1) (W); 21. Collaboration (e.g., the primary care practice has its own integrated behavioural health provider who collaborates with practice staff to serve all patients) (1) (S); 22. Active and on-going partnerships (1) (W); 23. Multiple stakeholder involvement (1) (W); 24. Clinicians and administrators involved as co-developers (1) (W); 25. Care coordination between patients and family-centred team (1) (W); 26. Using a team approach that prioritizes shared goals (1) (S); 27. Mutual trust (1) (W); 28. Effective communication (1; 11) (S; W); 29. Clear division of responsibilities (1) (S; W); 30. Creating partnerships between primary care providers (PCPs) and mental health specialists (1) (W); 31. Matching unmet needs to appropriate resources (1) (S); 32. Strengths-based, whole-of-family-involvement (1) (U); 33. Multi-agency wraparound care (1) (S); 34. Care coordination at a client level (1) (W); 35. Partnerships with families (1; 2) (U); 36. Coordination (between home care services, hospital services, schools and respite care services (1) (S); 37. Care (complex individualised, patient-centred curative and palliative care at home or in hospital settings) (1) (S; U; W); 38. Shared governance and collective decision-making (1) (W); 39. Information exchange between professionals within interprofessional groups (1) (W); 40. High level of density (1) (W); 41. Positive interpersonal relations and group reflection (1) (W); 42. Nurse as a tele-presenter (1) (W); 43. Inter-sectoral co-ordination (1) (S); 44. Social services/welfare services (1) (S); 45. Establishing and maintaining good relationships with staff from a broad range of agencies (1) (W); 46. Sharing common electronic health records (1; 10) (S); 47. Shared care plans (1) (W); 48. Primary care is ideally positioned to promote wellness and a holistic approach to health for the paediatric population (1) (S); 49. The engagement of clinical and professional leads in the process (1, 17) (W); 50. The synthesis of outcomes in joint planning (1) (W); 51. Multiple stakeholder involvement (1) (S); 52. Team-based approach (1) (W); 53. Effective collaboration and communication between providers (1) (W); 54. Decentralised decision-making opportunities (1) (S); 55. Access to a wide range of coordinated services in one place (1) (S); 56. Relationship trust (1; 2) (U; W); 57. Referral to and coordination with social services (1) (S; W); 58. Community-based agencies (1) (W); 59. Knowledge and decision-making sharing (1) (S); 60. Knowledge-sharing practices (1) (W); 61. Information exchange between professionals within interprofessional groups (1) (W); 62. Knowledge transfer (individuals with different professional backgrounds and knowledge and skills working together (1; 7) (W); 63. Individual and organisational values (1) (S; W); 64. Organisational workplace culture (1) (S); 65. Reliable team interactions between physicians and staff (1; 15) (W); 66. Medical and behavioural care providers (1) (S; W); 67. Maintaining a sense of common purpose (1) (W); 68. Multispecialty consultation or morbidity-oriented care (1) (S); 69. Holistic management of disease (1) (S; W); 70. Supervised monitoring (1) (W); 71. Longitudinal coordinated care (1) (S; W); 72. Knowledge and decision-making sharing (1) (S; W); 73. Shared professional responsibility and practices: governance – leadership group (1) (W); 74. Mutual trust (1) (W); 75. Coordination leads to co-location and leads to integration (1) (W); 76. Shared caseload (1) (S; W); 77. ‘Information exchange (1; 10) (W); 78. Developing multi-stakeholder partnerships (1; 15) (W); 79. A multidisciplinary team (1) (W); 80. Intervention delivery via community workers (1) (W); 81. Intervention delivery via external workers (1) (W); 82. Interdisciplinary delivery (1) (W); 83. Team of primary care and behavioural health clinicians work together with patients and families (1) (W); 84. Electronic medical record system (1) (S); 85. Patient-centred care (1; 5) (S; W); 86. Engagement (1) (S; U); 87. Nursing and social work collaboration and care coordination (1) (W); 88. A team of providers working collaboratively across disciplines, settings, levels of care, and sectors (1) (W); 89. Person-centred and holistic approach (1) (S; U); 90. Comprehensive delivery of quality services (1) (S); 91. Care coordination between patients and family-centred team (1) (W); 92. Provider knowledge (1) (W); 93. Effective communication (1) (U); 94. Patient-centred care (1; 5) (U); 95. Multidisciplinary team (1; 2) (W); 96. Organisational culture (shared values, beliefs, implicit norms that guide behaviour) (1) (S; W); 97. Organisational climate (employee-shared perceptions of the work environment) (1) (S); 98. Implementation –specific organisational factors: -implementation climate (staffs’ shared perceptions of the extent to which EBP implementation is expected) (1) (S); 99. Shared treatment plans (1) (W); 100. Strong collaboration (1) (W); 101. Multimodal approach (1) (S); 102. Interdisciplinary teams (1) (W); 103. Embedded specialists which provide counselling (1; 2; 8) (U; W); 104. Coordinating referrals to external specialists (1; 2; 3) (W); 105. 2nd model: collaborative relationships with pediatric primary care teams to provide telephone consultations (1; 2) (W); 106. 3rd model: embedded specialists (1; 2; 21) (W); 107. Interprofessional communication (i.e. communicating with patients, families, communities, and professionals in health and other fields in a responsive and responsible manner that supports a team approach) (1; 11) (U; W); 108. Collective impact-focus on community not individuals (1; 2) (S; W); 109. Coordinated, collective partnerships in multiple sectors such as government, health care, school/afterschool, workplace, and the community (1; 2) (W); 110. Information sharing through email (to pediatricians of the links to the BICP, highlighting the core features of the protocol for the management of the AB) (1; 11) (W); 111. Coordination (1) (S); 112. Management of referrals of children and young people to access medical care (1; 4) (S; U); 113. Shared governance and investment are essential to effect and sustain inter-agency change (1; 20) (S; W); 114. Coordinated/telephonic: increased collaboration between PCPs and behavioral health provider (1; 10) (S; W); 115. Multiple professionals collaborating (1) (W); 116. A holistic, family-centred approach (1; 3; 5) (S; W); 117. Addressing a broad range of needs in a timely manner (1) (S; U); 118. Shared decision making (1) (S; W); 119. Referral (1; 2; 4; 13) (S; U; W); 120. Coherent, continuous, and coordinated care (1; 13) (S; W); 121. ‘Information exchange’ (1; 10; 11) (W); 122. ‘Care process’ (Screening and assessment, Broad assessment of problems and the use of screening tools, StrongShared Care plan, Several perspectives and goals in a comprehensive care plan, Medium—strongReferral, Transition between care providers, Medium—strongTheme: Expertise Knowledge and training, Extending knowledge by means of training, StrongGuidelines, The use of evidence-based guidelines to support professionals, StrongSelf-efficacy, Confidence and comfort of professionals to provide integrated car) (1; 2) (S); 123. Electronic health record (1; 10) (S); 124. Focus on inherent strengths (strengths-based approach) (1) (S); 125. Shared plan of care (1) (W); 126. Information systems-electronic medical record, electronic prescriptions (1; 10) (S); 127. Integrated provider systems (1) (S); 128. Organisation of health care (1; 2) (S); 129. Coordination with client/family/school health systems (1) (U; W); 130. Referrals to specialists for consult (1; 2) (S); 131. Referrals for behaviour therapy (1; 2) (U); 132. On-site IC: interprofessional collaboration and communication (1) (W); 133. Tracking progress (1; 6; 9) (S); 134. Multidisciplinary treatment team (1; 2) (W); 135. Tracking progress (1; 6; 9; 13) (S); 136. Co-location in community health centres (1; 8; 14) (S); 137. Common care culture (1; 14) (W); 138. Shared leadership (1; 17) (W); 139. Mutual trust (1) (U; W); 140. Non-hierarchical relations (1, 11, 21) (W); 141. Active and on-going partnerships (1) (W); 142. Common understanding, and the “co-creation” effect of problem mapping (1) (W); 143. Electronic health records (1; 10) (S); 144. Multidisciplinary team: psychologist, physician, speech therapist, physiotherapist, special educator (1; 2) (W); 145. Supporting All Sectors to Take on a Meaningful Role in Child Health (1) (U; W); 146. Families experienced coordination across health, social, and education systems (1; 2; 3) (U); 147. Looking after the whole person (1) (S; U); 148. Beyond the key professional preference for conventional care that families experienced coordination across health, social, and education systems (1; 2) (U; W); 149. Each professional contributes to the care of the patient or an interdisciplinary program where professionals also directly interact with shared decisions taking into account the numerous factors contributing to outcomes (1) (W); 150. Electronic medical record (1; 10) (S); 151. Coordinated care (information exchange) (1; 6) (S; W); 152. Sharing common electronic health records (1; 10) (S: W); 153. Coordinated/telephonic models to fully integrated models (i.e. behavioral health care delivered in clinic with significant collaboration and coordination of treatment plan between behavioural health clinician and other sites) (1; 12; 13; 15) (S); 154. Electronic health records (1; 10) (S); 155. Shared responsibility (1) (W); 156. Interdisciplinary team (1) (W); 157. Shared care or collaborative care (1) (S; W); 158. Integrated electronic health record (1; 10) (S); 159. Strong collaboration (1) (S; W); 160. Knowledge transfer activities and shared learning among collaborators (1) (W); 161. Development and implementation of shared assessment tools and referral criteria (1; 9) (S); 162. Information exchange between professionals within interprofessional groups (1) (W); 163. Settings, levels of care, and sectors (1; 2) (S); 164. Strength-based and culturally competent, accessible language (1) (U); 165. Effective communication (1) (W); 166. Electronic health records (1; 10) (S); 167. Coordination (e.g. consultation between physical and behavioural health care providers) (1) (W); 168. Development of care coordination measurement instrument (identity record and encounter record) (1; 12) (W); 169. Consult on the scientific evidence for care processes (1) (S; W) 170. Strong network of professionals with a coordianted professional as a connector (1) (S; W) 171. Shared action plan (1) (W; S) 172. Setting up a learning community of professionals to exhange knowledge, experiences and tools (1; 2; 6; 7; 11; 15) (S; W) 173. Four-component model (vision; process; parners; finance) (1) (S; W; U) 174. Care provision through intersectorial teams (1; 2; 12; 15) (S; W) 175. Establishing basic structures for cooperation across interdisciplinary teams (1; 2; 12; 15) (S; W) 176. Integrates mental health services in general services (1; 4; 6; 12) (S; W) 177. shared care (1) (S; W); 178. collaborative care (1) (S; W); 179. coordinated care (1) (S; W). |
| **Two:**  **Stronger connections and partnerships (n=155)** | 1. Shared care records (2) (S); 2. A systems-based and collaborative approach (2) (S); 3. Involvement of cross-sector and multidisciplinary teams (2) (W); 4. Goal dictates the forming of a multidisciplinary team needed to assess the patient’s health issues (2) (W); 5. Consistent communication (2) (W); 6. Quality of care (2) (S); 7. Integrated services (2) (S); 8. Family–professional partnership (2) (U; W); 9. Ongoing hospital/community interface (2) (S); 10. Effective health and multi-agency agreements and funding arrangements (2; 20) (S); 11. Access to and input from specialised care (2) (S); 12. PCP considering consultant recommendations and implementing them when appropriate (2) (W); 13. Formation of partnerships between schools, families, and community health-care providers (2; 13) (S); 14. Decision support (2) (W); 15. Community resources and policies (2) (S); 16. Stepped-care treatment interventions (direct consultation with a child and adolescent psychiatrist) (2) (U); 17. Place-based neighbourhood initiatives (2) (S); 18. Collaborative planning (2) (W); 19. High levels of trust between parties (2) (U; W); 20. Emphasis on rapport building (2) (W); 21. Interpersonal collaboration (2) (W); 22. Community health (2) (S; U); 23. Complex/involved interactions (2) (W); 24. Collaborative models (2) (S); 25. Address social drivers (2) (S); 26. Care coordination and connection to social services (2) (S); 27. Need for shared vision across multiple professional disciplines (2) (W); 28. Relationship-based approach (2) (W); 29. Access to and input from specialised care (2) (S); 30. Previous positive working relationships (2) (W); 31. Bringing pediatric psychology to the “front lines” of community care (2; 6; 22); (W); 32. Improved quality of care (2) (S); 33. Integration of behavioral health into primary care (2) (S); 34. Community-hospital – accountable care organisation partnerships (2) (S); 35. Care coordination (2) (S); 36. Horizontal integration (2) (S); 37. School and medical collaboration (BMI screening) and vending machine removal (2) (S); 38. Consistent communication (2) (W); 39. Collaboration through learning (2; 5) (W); 40. Access to and input from specialised care (2) (S; U); 41. Accountability (2) (S); 42. Community organizations and health policy experts (2) (U; W); 43. The care coordination key elements framework based on triple aim (2) (S); 44. Creation of a state-wide partnership including parents and family advocates (2) (S; U; W); 45. Commitment and willingness to change (2) (W); 46. Perceptions of shared vision and goals (2) (W); 47. Horizontal integration (2) (S); 48. School and medical collaboration (BMI screening) and vending machine removal (2) (S); 49. Perceptions of shared vision, goals, and a common value base (2) (W); 50. High levels of trust between parties (2) (W); 51. Embedded in role descriptions (2) (W); 52. Stronger connections and partnerships: involvement of cross-sector and multidisciplinary team (2) (W); 53. Stakeholders working collaboratively across disciplines, settings, levels of care, and sectors (2) (S); 54. Physician-parent shared decision-making during treatment planning (2) (U; W); 55. Shared decision-making (2) (S; W); 56. Collaboration with social work (2) (S); 57. Case management (2) (W); 58. Primary care/behavioural healthcare collaboration (basic collaboration from a distance (separate systems) - basic collaboration on-site (consult model only; regular communication) - close collaboration in a partly IC system – close collaboration in a fully IC system (shared systems and facilities) (2) (S; W); 59. Collaborative consultation (2) (S; W); 60. ‘Care process’ (2) (S); 61. Horizontal integration (2) (S); 62. Quality use of medicine to minimise medication errors in the community (2) (S; W); 63. Developing strong and enduring partnerships (2; 15) (W); 64. Access to and input from specialised care (2) (U); 65. Organisation of care: lifespan approach (2) (S); 66. Advocacy for the model and inter-agency partnerships (2) (W); 67. Interdisciplinary team (medical, mental health and child welfare professionals) (2) (W); 68. Interdisciplinary team (medical, mental health and child welfare professionals) (2) (W); 69. Collaboration: values and ethics (i.e., working with individuals of other professions to maintain a climate of mutual respect and shared values) (2) (W); 70. Horizontal integration (2) (S); 71. A centralized mental health telephone program (2; 10) (S; W); 72. Stepped care” : advancement of treatment intensity for service recipients who are not improving (2) (S; U); 73. Inter-organizational context (connections among organizations or units of the outer and inner settings) (2) (S); 74. Mechanisms for increasing treatment intensity (2; 9) (S); 75. Horizontal integration (2) (S); 76. Multidisciplinary teams (2) (W); 77. Stepped care”: advancement of treatment intensity for service recipients who are not improving (2) (S; U); 78. Vertical integration (although partnerships outside of health care) (2) (S); 79. Collaboration: values and ethics (i.e. working with individuals of other professions to maintain a climate of mutual respect and shared values) (2) (S); 80. Horizontal integration (2) (S); 81. Relationships as a trigger (place-based and collective impact approaches): a spirit of goodwill, high levels of trust between parties, norms of reciprocity and adaptability, a sense of obligation among group members, embedded ties through strong and enduring relationships (2; 8) (S; W); 82. Forming professional partnerships (2) (W); 83. The implementation of an integrated youth team through strategic, tactical and operational control rooms to offer coordinated services (the central component of this integration project) (2; 18) (S); 84. Co-designing innovation requires sustained commitment long-term (2; 13; 19) (U); 85. Integrated: direct collaboration and integration in medical team (2) (S); 86. Potential co-joint treatment planning (2; 14) (S; U); 87. Interprofessional collaboration (2; 14) (W); 88. Comprehensive and up-to-date shared care plans (2; 6; 9) (W); 89. Strengthening the capacity of families (2; 3) (U); 90. Case management (2) (W); 91. Decision support (2) (W); 92. Clinical guidelines-AAP/NICHQ; templates for primary care screening tools (2; 6; 9) (W); 93. Collaborative practice between dental, medical, and other pediatric primary care provider (2) (W); 94. Collaborative planning (2) (S); 95. Parents acting as active agents so called “family partners” (2; 3; 5) (U); 96. Vertical integration (with some horizontal aspects) (2) (S); 97. Staff/family partnership (2; 18) (U; W); 98. Ensuring the full participation of youth and their parents/caregivers (2; 3) (U); 99. Child-friendly counselling context (2; 3) (U); 100. A multidisciplinary team (2) (W); 101. Connecting Services Together (2) (S); 102. Importance of Formal and Informal Contacts (2) (U; W); 103. Postdoctoral fellows in clinical/school psychology serving as collaborative care therapists with a local community member serving as a community health partner (partnership with community through a local citizen) (2) (W); 104. Efficient communication between primary and mental health care providers (2; 11; 15) (W); 105. Importance of comprehensive screening and successful mental health linkage (2; 4; 6) (S); 106. Multidisciplinary collaboration and enhancement of primary care providers’ capacity (2) (W); 107. Joint responsibility (2) (W); 108. Liaise between primary care providers and a decision support panel (2) (W); 109. Leveraging other family, social, and organisational relationships (2; 18) (U); 110. Stronger connections and partnerships: forming professional partnerships (2) (W); 111. Stakeholders working collaboratively across disciplines (2) (W); 112. Need for shared vision across multiple professional disciplines (2) (W); 113. Comprehensive support system for nursery schools (2; 3) (S); 114. Thinking systemically (2; 7; 12; 15) (W); 115. Build and rebuild relationships (2) (W); 116. Population-centred care (3) (U); 117. Integration of services (physical and mental health support/services) (2) (S); 118. Shared decision-making (2) (W); 119. The time needed to build professional relationships (2) (W); 120. Shared care records between the secondary and primary care providers (2) (W); 121. Collaboration (e.g. the primary care practice has its own integrated behavioural health provider who collaborates with practice staff to serve all patients) (2) (W); 122. Vertical integration (2) (S); 123. Strengthen foundation-clinic-academic partnership (2) (W); 124. Create work flow analyses prior to implementation (2; 6; 12) (S); 125. Educational/medication support strategies (2; 6) (S); 126. Partnerships with families (1; 2) (U; W); 127. Partnerships with families (1; 2) (U); 128. Relationship trust (1; 2) (U; W); 129. Multidisciplinary team (1; 2) (W); 130. Embedded specialists which provide counselling (1; 2; 8) (U; W); 131. Coordinating referrals to external specialists (1; 2; 3) (W); 132. 2nd model: collaborative relationships with pediatric primary care teams to provide telephone consultations (1; 2) (W); 133. 3rd model: embedded specialists (1; 2; 21) (W); 134. Collective impact-focus on community not individuals (1; 2) (S; W); 135. Coordinated, collective partnerships in multiple sectors such as government, health care, school/afterschool, workplace, and the community (1; 2) (W); 136. Referral (1; 2; 4; 13) (S; U; W); 137. ‘Care process’ (Screening and assessment, Broad assessment of problems and the use of screening tools, StrongShared Care plan, Several perspectives and goals in a comprehensive care plan, Medium—strongReferral, Transition between care providers, Medium—strongTheme: Expertise Knowledge and training, Extending knowledge by means of training, StrongGuidelines, The use of evidence-based guidelines to support professionals, StrongSelf-efficacy, Confidence and comfort of professionals to provide integrated car) (1; 2) (S); 138. Organisation of health care (1; 2) (S); 139. Referrals to specialists for consult (1; 2) (S); 140. Referrals for behaviour therapy (1; 2) (U); 141. Multidisciplinary treatment team (1; 2) (W); 142. Multidisciplinary team: psychologist, physician, speech therapist, physiotherapist, special educator (1; 2) (W); 143. Families experienced coordination across health, social, and education systems (1; 2; 3) (U); 144. Beyond the key professional preference for conventional care that families experienced coordination across health, social, and education systems (1; 2) (U; W); 145. Settings, levels of care, and sectors (1; 2) (S); 146. Collaboration between municipalities and Care for obesity programme though developing a national mode (2) (S; W) 147. Setting up a learning community of professionals to exhange knowledge, experiences and tools (1; 2; 6; 7; 11; 15) (S; W) 148. Care provision through intersectorial teams (1; 2; 12; 15) (S; W) 149. Establishing basic structures for cooperation across interdisciplinary teams (1; 2; 12; 15) (S; W) 150. Delivery system redesign (2) (S; W); 151. Active engagement of family members or guardians (2; 4; 5; 6; 11; 18) (S; U) 152. Risk-prevention psychoeducation for guardians of individuals with suicide-related emergencies (2) (S; U); 153. Risk -targeted follow-up for caregivers of individuals with suicide-related emergencies (2) (S; U) 154. Hub-spoke approach supporting a snall and independently owned primary care setting (2) (S; U; W) 155. stepped care (2) (S; U) |
| **Three:**  **Empowerment of service users (n=100)** | 1. Service users empowerment (3) (S; U); 2. Patient involvement and self-management (3) (U); 3. Flexible model (3) (S); 4. Family-centred care coordination (3; 5) (U; W); 5. Building families agency (3) (U); 6. Patient-centred care (3; 5) (U); 7. Child-centred approach (3) (U; W); 8. Capturing the “child’s voice”(3) (U); 9. Patient education and advice (3) (U); 10. Contextualising materials to youth culture (3) (U); 11. Influence recruitment (opt-out and opt-in recruitment for participants) (3) (W); 12. Safe and nurturing environment for families (3) (U); 13. Navigation of system (for patients) (3) (U); 14. Proactive guidance and support (3) (U; W); 15. Programme Flexibility (3) (S); 16. Increased interaction (“crossing of paths”) (3) (U); 17. Trauma-informed care (3) (U; W); 18. Shared decision-making between clinicians and family members (3) (U; W); 19. Flexibility and adaptiveness of staff (3) (W); 20. The need for team members to be available and work differently with each other and with the patient (3) (W); 21. Empowerment through ‘bottom-up’ approaches in the development and improvement of health and social care services in their local area (3) (S; W); 22. Patient-centred delivery system for at-risk youths (3) (U); 23. Development of individualized treatment recommendations (3) (U; W); 24. Clarifying the right diagnosis for youths (3; 24) (U; W); 25. Respectful and open atmosphere to reduce power imbalances (3) (S; U; W); 26. Youth as co-investigators and co-creators of the initiative (3; 18) (U); 27. Family-based care capacity building (3) (U); 28. Caregivers can participate in programming (3) (W); 29. Parental empowerment (3) (U); 30. Person-centredness (3) (S); 31. Families/carers must be empowered and engaged (3) (U); 32. Regular clinical visits: ambulatory consultation and consultations both “on demand” and through electronic media (3) (S; W); 33. Psychological support to family (3) (U); 34. Empowerment of service users’ information (3) (U); 35. Exchange and individualised care plans: individualised care plans, developed with family (family centred) (3; 5) (U; W); 36. ‘Child’s environment’ (3) (U); 37. Self-management interventions aim to help individuals better manage their medical treatment and cope with the impact of the condition on their physical and mental well-being (3) (U); 38. Youth-friendly primary care systems (3) (U); 39. Personal: transition readiness, self-care, life-social skills (3) (U); 40. Integrated approach which promotes essential self-advocacy, self-care and life skills (3) (S; U); 41. Empowering families (3) (U); 42. Individual counselling (3) (U; W); 43. Adaption of care to indigenous sociocultural specificities (3) (U); 44. Strengthening culture and identity (3; 11) (S; U; W); 45. Flexibility of integrated visits depending on a patient's needs (3) (U); 46. Produce individualized recommendations (3) (U; W); 47. Referral support and follow up training (3; 5) (U; W); 48. Families: patient journey map (3; 13) (U); 49. Information sheets for families (3) (U); 50. Warm-handoffs for direct patient engagement (3; 5) (U); 51. Support for remote consultation, including psychiatry and medication management (3; 10) (S; U); 52. Meeting youth at a location of their choice (3) (U); 53. Freedom to adapt treatment plans (3) (S; W); 54. Parental involvement in decision making processes (3; 18) (U); 55. ‘Child’s environment’ (their description, and strength of evidence: Family-centered focus, A holistic approach on a family’s welfare, medium - strong) (3) (U); 56. Group and individual psychotherapy (3) (U); 57. Policies to accommodate persons with ADHD School-Special education program (3; 6) (W); 58. Individualised education plans (3) (S; U; W); 59. Community organisations-support groups ADA (3) (U); 60. Family-based treatment (3) (U); 61. Familiar setting (3) (U); 62. Evidence-based, patient-centred care (3) (U); 63. Feeling heard and receiving contextualized support (3) (U); 64. Material resources that flow (or not) from being heard that professionals involved children and caregivers in treatment (3; 20) (U); 65. Respect for young person’s autonomy (3) (U); 66. Trust is built on the recognition of professional learning and enhanced through physical presence (3) (U; W); 67. Professionals involved children and caregivers in treatment (3; 18; 21) (U; W); 68. Young adult clinic (3) (U); 69. Acknowledging to a patient that when we take on the responsibility to care for a patient with IBD (3) (U); 70. Care managers to facilitate IC Foreign language specialists (Spanish –speaking SWs) (3; 16; 24) (W); 71. Emphasising patient-reported outcomes (3; 6) (W); 72. Family-centred care coordination (3; 5) (U); 73. Patient-centred care (3) (U); 74. Prolonged visits (3) (S); 75. Parents have freedom to select best treatment for their child multi-informant assessment (3) (U); 76. Building trust (3) (U; W); 77. Meeting clients on their own terms (3) (U); 78. Client empowerment (3) (U); 79. Flexible service delivery (3) (S); 80. Client empowerment (3) (U; W); 81. Participation of adolescents and parents (3) (U); 82. Population-centred care (3) (U); 83. Patient-centred focus (3) (U); 84. Addressing the child’s and family’s needs (1; 3) (U; W); 85. A holistic, family-centred approach (1; 3; 5) (S; W); 86. Strengthening the capacity of families (2; 3) (U); 87. Parents acting as active agents so called “family partners” (2; 3; 5) (U); 88. Ensuring the full participation of youth and their parents/caregivers (2; 3) (U); 89. Child-friendly counselling context (2; 3) (U); 90. Comprehensive support system for nursery schools (2; 3) (S); 91. Coordinating referrals to external specialists (1; 2; 3) (W); 92. Families experienced coordination across health, social, and education systems (1; 2; 3) (U) 93. Personal appoach and broad perspective to each family (avoiding 'one size fits all' approach) (3) (U; S) 94. Support in recovery by staff (3) (U; W) 95. Support from caregivers through communication, information provision (3) (U; W) 96. Individualised care with consideration of complexities (3; 6) (U; W) 97. Approaches youth in their environment to improve undertsanding about their context (3; 8) (S; U) 98. Self-management support (3) (U) 99. Self-management support and health promotion (3) (U) 100. Resilience building and mental health first aid (3) (U) |
| **Four:**  **Early detection and prevention (n=92)** | 1. Coordination of preventative care with evidence-based strategies (4) (S); 2. The promotion of routine annual mental health screening in primary care (4) (W); 3. Specify and focus on the overarching goal identified by a patient (4) (U); 4. The treatment plan should support health literacy (4) (U); 5. Early diagnostics (school-based assessment and school-based intervention) (4) (W); 6. Early screening (4) (U); 7. Safeguarding (4) (U); 8. Early identification: proactive and preventive assessment of prenatal problems (4) (S; W); 9. Additional behavioural consultations (4) (W); 10. Screening and early short-term treatment (4) (U; W); 11. Early response based on screening (4) (U; W); 12. Integrated psychology trainees in a safety net hospital's pediatric ED and urgent care (4; 13) (W); 13. Coordination included education for patients and guidance on monitoring symptoms, scheduling a clinic visit (4) (U); 14. Early identification of severe injury (early identification of severe injury) (4) (S); 15. Provision of close supervision of family (4) (S; U); 16. Early screening (4) (U); 17. Increased ambulatory visits (4) (S); 18. Early referral (4) (U; W); 19. Immunisation (4) (S; U); 20. Physical examination and specific screening activity (4) (U); 21. Health supervision (4) (U; W); 22. Reinforce anticipatory guidance (4) (U); 23. Developmental surveillance (4) (U; W); 24. Early Detection and Prevention (Early assessment and intervention to prevent crises) (4) (S; U); 25. Focus on primary care (4) (S); 26. Health sector collaborate with other sectors (4) (S); 27. Early Detection and Prevention (Early assessment and intervention to prevent crises) (4) (S; U); 28. Primary care is important to transformative paediatric payment and delivery models (4) (S); 29. Brief interventions onsite at the primary care clinic (4; 14) (U; W); 30. Integrated in community (4) (S); 31. Screening of risks (4; 9) (S); 32. Prevention in the form of anticipatory guidance and identifying mothers at risk for depression (4) (S; U); 33. Routine assessment (4) (S; U); 34. Early assessment and intervention to prevent crises (4) (S; U); 35. Improved follow-up care in-depth diagnostic and family needs assessment (4; 6; 9) (S; U; W); 36. Measurement-based care (4) (S); 37. Early detection and prevention: screening for health needs (4) (S; U; W); 38. Early assessment and intervention to prevent crises (4) (S; U; W); 39. Staff training (diagnosis-specific training) (4) (W); 40. Early detection (4) (S; U); 41. Interventions to improve service utilization; early detection and pathways-to-care (4) (S); 42. Implementation of a cross-age and interdisciplinary mobile early detection team (4) (S; U); 43. Screening (4) (S); 44. Brief intervention and/or referrals for specialized services (4) (S); 45. Assessing the need for hospitalisation (4) (S; U); 46. Evidence-based behavioural treatment (4) (S; W); 47. Medical crisis procedures (4) (S); 48. Screening, assessment and referral; community based services (4) (S; U); 49. Measurement-based care (4) (S); 50. Early identification and treatment (4) (S; U; W); 51. Medication management (4) (U); 52. Progress monitoring (4; 6) (S; U; W); 53. Primary care is responsible for detection, initial assessment (4) (S); 54. Development, implementation of a treatment plan (4) (S); 55. Coordination of referrals to specialists as needed and monitoring progress (4) (S); 56. Supervised health checks (4) (S; U); 57. Early detection and support (4) (S; U; W); 58. Crisis evaluation (acute suicide risk evaluation) (4) (S; U); 59. Symptom awareness (4; 6) (U); 60. Innovative systems approach through greater prevention and disease management by conducting risk assessments and obtaining a greater awareness in oral health literacy (4) (S); 61. Infrastructure development (4) (S); 62. Early intervention (4) (S); 63. Screening (4) (S; U); 64. Incoming referral (4) (U); 65. Screening of risks (4; 9) (S; W); 66. Prevention of treatment (4) (S); 67. Reinforce anticipatory guidance (4; 6) (S); 68. Registries and systematic screening (4) (S); 69. Timely identification of mental health needs (4) (S); 70. Multidisciplinary collaboration (4) (W); 71. Evaluation of patients’ psychological progress (4; 9) (S; U); 72. Use of data from multiple informants (4; 6) (S; W); 73. Early assessment and intervention to prevent crises (4) (S; W); 74. CPS monitoring on sight (4; 9; 13) (S); 75. Systematic screening and monitoring with treatment to target (4; 6; 9) (S); 76. Measurement-based care (4) (S); 77. Delivery of evidence-based mental health services (4) (S); 78. “Back up” of psychiatrists services (4) (W); 79. Progress monitoring (4; 6) (W); 80. Management of referrals of children and young people to access medical care (1; 4) (S; U); 81. Importance of comprehensive screening and successful mental health linkage (2; 4; 6) (S); 82. Referral (1; 2; 4; 13) (S; U; W); 83. Screening for housing instability and food insecurity (4) (W; U); 84. Introduction of performance measures: kindergarden readiness promotion bundle (4;awareness 9) (S; U; W); 85. Introduction of performance measures: screening for clinical depression (4; 9) (S; U; W); 86. Integrates mental health services in general services (1; 4; 6; 12) (S; W) 87. Enhances early intervention in municipalities and supports prioritisation of care for the specialist sector (4) (S) 88. Progress monitoring (4; 6) (S; U; W); 89. Early Identification (4) (S) 90. Active engagement of family members or guardians (2; 4; 5; 6; 11; 18) (S; U) 91. Risk-prevention psychoeducation for guardians of individuals with suicide-related emergencies (2; 4) (S; U); 92. Risk -targeted follow-up for caregivers of individuals with suicide-related emergencies (2; 4) (S; U) |
| **Five:**  **Training of parents (n=76)** | 1. Individual care record system (5) (S); 2. Single-point of access (5) (S); 3. Clinical sustainability of the model (5) (S); 4. Family education (5) (U); 5. Individualised care plans (5) (S); 6. A named paediatrician with appropriate expertise as a central person (leader) (5) (W); 7. Meaningful involvement of parents and families (5) (W); 8. Collaboration between professionals and parents (5) (S; W); 9. Warm hand-off (5) (W); 10. Parents involved in child’s treatment (psycholeducation) (5) (U); 11. Person-centred and holistic (5) (U; W); 12. Co-development and co-design (5) (U; W) 13. Self-management support (5) (U); 14. Safe transitions of care (5) (U); 15. Continuous improvement (5) (S); 16. Scope of the familiar emergency response role (5; 23) (W); 17. Providing a single point of contact (5) (S); 18. Re-traumatizing family (5) (U; W); 19. Self-management support (5) (U); 20. Single point of contact (5) (W); 21. Training of lay public and peers (5) (U); 22. Capacity building (5) (S); 23. Services and practitioners working together in a coordinated and client-focused way (5) (W); 24. Culturally appropriate care (5) (U); 25. Early childhood education (5) (S; U); 26. Individualized hand-offs to treatment resources (reduce the need for families to start over) (5) (U; W); 27. Co-design and co-production of the initiative in partnership with families and service partners (5) (S; U; W); 28. Co-development and co-design (5) (S; U; W); 29. Face-to-face interactions (5) (U; W); 30. Specialized asthma education to families (5) (U); 31. Incorporate community health workers and provide in-home asthma education (5) (U); 32. Family involvement (5) (U); 33. Partial hospitalization (PHP) (5) (S; W); 34. Intensive outpatient programs (IOP) (5) (S; U); 35. Providing a single point of contact (5) (S; U; W); 36. Medical provider available 24 hours per day (5) (U; W); 37. Promotion of adherence between staff and patients (5) (U; W); 38. “No drop-out" policy (5) (S; U); 39. Individual: person-centred care in a single process across time, place, and discipline (5) (U; W); 40. On-going monitoring and follow-up (5) (S; W); 41. Chronic condition self-management education (5) (U); 42. The role of primary care providers in educating patients and families (5) (U; W); 43. Necessary skills through care provision (5) (U); 44. Family education (5) (U); 45. Collaboration (CC model): a team of coordinated providers (5) (W); 46. Play therapy and preoperative teaching for families; empowerment of families through teaching (5; 6) (W); 47. Warm handoff consultation (5) (U; W); 48. Information provision to families about prevention development of the eSanoweb page (which includes all the AB pathway tools as well as educational videos for parents dissemination by e-mail to pediatricians of feedback on levels of and/or improvement in pharmacologic prescribing) (5) (U); 49. Display of an educational poster in waiting and consultation rooms with key messages for families (5; 6) (U); 50. Display of an informative poster in the lobby of the hospital during AB seasons (5; 6) (S; U); 51. Campaigns on social media with educational messages targeting families drafted by the health care professionals themselves (5; 6) (S; U); 52. Health management plan developed (5; 6) (S; U); 53. Collaboration between professionals and parents (5) (U; W); 54. Warm handoffs (5) (S); 55. Health systems: self-management support (5) (U); 56. Unconditional support and empowerment of the family (5) (U); 57. Consistent and direct communication (5) (U; W); 58. Dedicated case management or navigation to help families access and engage with mental health care (5; 16) (W) 59. Psychoeducational and skills training for families-education to parents (5) (U); 60. Co-design and co-production of the initiative in partnership with families and service partners (5) (U); 61. Personalised, family-centred approach (5) (U); 62. Collaborative Inter-professional care that is patient-centred (5) (W); 63. Family-centred care coordination (3; 5) (U; W); 64. Patient-centred care (3; 5) (U); 65. Collaboration through learning (2; 5) (W); 66. Exchange and individualised care plans: individualised care plans, developed with family (family centred) (3; 5) (U; W); 67. Patient-centred care (1; 5) (S; W); 68. Patient-centred care (1; 5) (U); 69. Referral support and follow up training (3; 5) (U; W); 70. Warm-handoffs for direct patient engagement (3; 5) (U); 71. Family-centred care coordination (3; 5) (U); 72. A holistic, family-centred approach (1; 3; 5) (S; W); 73. Parents acting as active agents so called “family partners” (2; 3; 5) (U) 74. Active engagement of family members or guardians (2; 4; 5; 6; 11; 18) (S; U) 75. Risk-prevention psychoeducation for guardians of individuals with suicide-related emergencies (2; 4; 5) (S; U); 76. Risk -targeted follow-up for caregivers of individuals with suicide-related emergencies (2; 4; 5) (S; U) |
| **Six:**  **Awareness (n=68)** | 1. Meeting the medication needs of parents (6) (U; W); 2. Addressing the social determinants of health (6) (S); 3. Iterative and reflective development and implementation cycles (6) (S; W); 4. Evidence-informed interventions (6; 7; 9) (S); 5. Achievement of a high level of diagnostic clarity sooner (6) (S); 6. Longer appointment times may be a necessary component of high-quality transitional care (6) (S; U); 7. Comprehensiveness in implementation planning (6; 7) (S); 8. Timely response (6) (S); 9. Equality (6) (S); 10. Education (6) (W); 11. Educational/medication support strategies (2; 6) (S); 12. Use of population registry helps prioritising severity of medical needs (6) (S); 13. Developmental process of growing up (6) (S; U); 14. Managing whole child pathway (6) (U); 15. Accountability through consultation and supervision (6) (S; W); 16. Embedded psychiatric care (6) (S); 17. Families awareness (6) (U); 18. Log keeping (6) (U; W); 19. Brief psychotherapy (6) (U); 20. Continuing education (6) (W); 21. Inclusion in the organization’s computer program of a pop-up window associated with the diagnosis of AB that automatically displays the most important documents associated with pathways (6; 10; 11) (S; W); 22. Clear allocation of tasks and responsibilities (6; 23) (W); 23. ‘Preconditions’ (Time, Time to address a broad spectrum of problems and for inter-professional collaboration) (6) (S); 24. ‘Inter-professional collaboration’ (6) (W); 25. Clear protocols facilitating intervention screening (6) (S); 26. The key health-worker understood the health needs of the family in context (6; 16) (U; W); 27. Each theme also had sub-themes that the key health-worker understood the health needs of the family in context (6) (W); 28. Need-driven care (6) (S); 29. Provision of support and resources; realistic expectations (6) (S; W); 30. Care delivery within and outside clinic (6; 8) (S); 31. A solution-focused approach (6) (S); 32. Population-based care (6) (S); 33. Use of individual financial records (6) (U); 34. Advise on measurement-based care (6; 9) (S); 35. Integrate evaluation planning during care model development (6) (S); 36. Bringing pediatric psychology to the “front lines” of community care (2; 6; 22); (W); 37. Improved follow-up care in-depth diagnostic and family needs assessment (4; 6; 9) (S; U; W); 38. Play therapy and preoperative teaching for families; empowerment of families through teaching (5; 6) (W); 39. Progress monitoring (4; 6) (S; U; W); 40. Display of an educational poster in waiting and consultation rooms with key messages for families (5; 6) (U); 41. Display of an informative poster in the lobby of the hospital during AB seasons (5; 6) (S; U); 42. Campaigns on social media with educational messages targeting families drafted by the health care professionals themselves (5; 6) (S; U); 43. Health management plan developed (5; 6) (S; U); 44. Comprehensive and up-to-date shared care plans (2; 6; 9) (W); 45. Clinical guidelines-AAP/NICHQ; templates for primary care screening tools (2; 6; 9) (W); 46. Policies to accommodate persons with ADHD School-Special education program (3; 6) (W); 47. Symptom awareness (4; 6) (U); 48. Tracking progress (1; 6; 9) (S); 49. Tracking progress (1; 6; 9; 13) (S); 50. Coordinated care (information exchange) (1; 6) (S; W); 51. Reinforce anticipatory guidance (4; 6) (S); 52. Emphasising patient-reported outcomes (3; 6) (W); 53. Use of data from multiple informants (4; 6) (S; W); 54. Systematic screening and monitoring with treatment to target (4; 6; 9) (S); 55. Progress monitoring (4; 6) (W); 56. Create work flow analyses prior to implementation (2; 6; 12) (S); 57. Importance of comprehensive screening and successful mental health linkage (2; 4; 6) (S) 58. Gaining perspective on their illness through completing measures (U; S) (6) 59. Group treatment (6) (U; W) 60. Individualised care with consideration of complexities (3; 6) (U; W) 61. Increasing awareness measures (6) (S; W) 62. Setting up a learning community of professionals to exhange knowledge, experiences and tools (1; 2; 6; 7; 11; 15) (S; W) 63. Integrates mental health services in general services (1; 4; 6; 12) (S; W) 64. Risk stratification (6) (S; W) 65. Progress monitoring (4; 6) (S; U; W) 66. Active engagement of family members or guardians (2; 4; 5; 6; 11; 18) (S; U) 67. Risk-prevention psychoeducation for guardians of individuals with suicide-related emergencies (2; 4; 5; 6) (S; U); 68. Risk -targeted follow-up for caregivers of individuals with suicide-related emergencies (2; 4; 5; 6) (S; U) |
| **Seven:**  **Workforce development**  **(n=65)** | 1. Training of staff (7) (W); 2. An investment of resources (7) (S); 3. A culture of evaluation and improvement (7) (S); 4. Training of staff (7) (W); 5. Therapists to receive e-learning training (7) (W); 6. Nurse preparation (7) (W); 7. Standardising specialist training (7) (W); 8. Training and on-going supervision (7) (W) 9. Evidence –based behavioural treatment (7) (S; W); 10. Pediatric-trained staff (7) (W); 11. Guidelines regarding confidential information (7; 19) (S); 12. A specific role of a psychologist (7) (W); 13. The CCM delivery system design (The CCM model urges practices to define roles for members of the care team with doctors and other highly trained staff addressing acute problems, and training other team members who do more patient engagement and routine work) (7; 23) (S); 14. Training and on-going supervision (7) (W); 15. A culture of evaluation and improvement (7) (W); 16. Advanced interprofessional training and education in integrated care (7) (W); 17. Ongoing training and monitoring (7) (W); 18. Evidenced-based care (7; 9) (S; W); 19. Learning of staff (7) (W); 20. Personal and professional training (7) (W); 21. Trauma-focused training and learning (7) (W); 22. Division of care coordination tasks based on care coordinators’ training (7) (W); 23. Learning from practice and learning from families (7) (U; W); 24. Training of behavioral health providers (7) (W); 25. Knowledge of psychological principles (7) (W); 26. Evaluation of outcomes (7) (S); 27. Dialogue conferences (7) (W); 28. Involvement of staff in the development of policies and procedures (7; 21) (W); 29. Guidelines regarding confidential information (7; 19) (S); 30. Accessibility: better access to care (7) (S; U; W); 31. Training of specialist staff, pediatricians, child and adolescent psychiatrists, and other behavioural health providers develop new skills and evolve practice processes to engage youth with evidence-based care approaches (7) (W); 32. A culture of improvement and evaluation (7) (S); 33. Training and on-going supervision (7) (W); 34. Staff training (7) (W); 35. Regular practice-team meeting to problem solve and assess progress (7) (W); 36. Four-year trialogue interventions to improve mental health literacy, stigma and service utilization (a trialogue ‘awareness campaign’ including cinema spots, city-light posters) (7) (S); 37. (Expansion of the early detection service for psychosis to a cross-age and interdisciplinary early detection service for all mental disorders (aged 12–29 years)); expansion of the pre-existing catchment area network to improve the service utilization (7) (S; U); 38. Training of staff (7) (W); 39. Enhanced involvement of primary care providers in treating youth with eating disorders (7) (U; W); 40. A tertiary-care-based nurse practitioner (7; 16) (W); 41. Receive skills training in mental health interventions (7) (W); 42. Well-defined roles (7) (W); 43. Training and support (BHCs and PCPs) (7) (W); 44. Nurse preparation (7) (W); 45. Roles and responsibilities (i.e., using the knowledge of one’s own role and those of other professions to appropriately assess and address healthcare needs) (7; 21) (S; W); 46. Information-training sessions including review of current evidence (7) (W); 47. Staff training (7) (W); 48. Training and guidance (7) (W); 49. Trusting peer training (7) (W); 50. Trauma-informed care (7; 19) (S); 51. Evidenced-based care (7; 9) (S); 52. Staff training (7) (W); 53. Triple P training (7) (S); 54. Informal knowledge transfer (7) (W); 55. Regular practice-team meeting to problem solve and assess progress; Training of education staff (informed knowledge of CPS roles) and joint training (7) (W); 56. Education for caregivers (including psychoeducational) (7) (U); 57. Training of specialists (7) (W); 58. Evidence-informed interventions (6; 7; 9) (S); 59. Comprehensiveness in implementation planning (6; 7) (S); 60. Knowledge transfer (individuals with different professional backgrounds and knowledge and skills working together (1; 7) (W); 61. Thinking systemically (2; 7; 12; 15) (W); 62. Specilist training for a nurse as a coordinator (7) (W); 63. Setting up a learning community of professionals to exhange knowledge, experiences and tools (1; 2; 6; 7; 11; 15) (S; W) 64. training of staff (7) (W) 65. Provisioning education programs for nursing staff (7) (W; S) |
| **Eight:**  **Accessibility and availability**  **(n= 70)** | 1. Accessible care: equitable availability of consistently high-quality, prompt, and accessible services (8) (S); 2. Geographical variation (8) (S); 3. Community-based services and support (8) (S; U); 4. Accessible accommodation, equipment and transport (8) (S); 5. Access to primary and tertiary care (8) (U); 6. Logistical medical support (8) (S); 7. Better access to care (8) (S; U); 8. Improving access (8) (S; U); 9. Home visiting and a place-based service (8) (W); 10. Accessibility, flexibility, and service navigation (8) (S); 11. Local level non-governmental organisations (8) (S); 12. Service flexibility (8) (S); 13. Facilitating access to internal/external services (8) (S); 14. Linking families with services (8) (U); 15. Adaptability of the model (8) (S); 16. One step access to walk-in services (8) (S); 17. Extended hours of operation and cost free (8) (S); 18. Accessibility, flexibility, and service navigation (8) (S; U); 19. Multiple entry points (self-referral, drop-in) (8) (S; U); 20. Better access to care (8) (S; W); 21. Home visiting programs (8) (S; U); 22. Consultation, infrastructure support and technology (8) (S); 23. Home treatment (8) (U; W); 24. Extended hours (8) (S); 25. Off-site mental health provision (8; 13) (S; U; W); 26. Phone consultations (8) (S); 27. ‘Preconditions’ (8; 12); 28. Availability of care (8) (S; U); 29. Location of clinics (8) (S); 30. Access to services and resources (8) (S; U); 31. Flexibility of communication methods (e.g., email, phone) (8) (S; U); 32. Reducing barriers to care (8) (S; U); 33. Access to care (8) (S; U); 34. Accessibility: better access to care (8) (S; U); 35. Existing resource support (8) (S); 36. Signposting to local resources (8) (S; U); 37. Increased access (8) (S; U); 38. Service flexibility (8) (S); 39. Walk-in sessions (8) (U); 40. Provision of necessary resources for the child and family (8; 25) (U); 41. Access to care (8) (S; U); 42. Health care providers-availability (8) (S; W); 43. Primary care and psychiatric providers (8) (W); 44. One treatment environment (‘one stop shop’) (8; 14) (S; U); 45. Child-appropriate language (8; 18) (U); 46. Fast-track appointments (8) (S); 47. Access to specialised care (8) (S; U); 48. Expedited access to outpatient psychiatric consultation (8) (S; U); 49. Phone consultations (8) (S; U; W); 50. Alternative approaches to solving complex problems (8) (U); 51. Measurement based (9) (S); 52. Accessibility/quality of appropriate care for behavioural health (mental health/emotional wellbeing) needs (8) (S); 53. Better understanding of roles and responsibilities (8) (W); 54. Co-location in community health centres (1; 8; 14) (S); 55. Relationships as a trigger (place-based and collective impact approaches): a spirit of goodwill, high levels of trust between parties, norms of reciprocity and adaptability, a sense of obligation among group members, embedded ties through strong and enduring relationships (2; 8) (S; W); 56. Co-location in community health centres (1; 8; 14) (S); 57. Care delivery within and outside clinic (6; 8) (S); 58. Creating commitment (by presentations, conferences, etc) (8; 19) (S; U); 59. Easy access provision (phone, short waiting time, limited need for assessment forms to fill out) (8) (S; U) 60. Care reaches beyond the age of 18 (8) (S; U) 61. Care includes housing; shared responsibilities across different levels of care by avoid unequal access to services (8) (S; U; W); 62. Systematic care delivery provision (8) (S); 63. Flexible and low threshhold care (8) (S); 64. Approaches youth in their environment to improve undertsanding about their context (8) (S; U) 65. One-stop health and socials service delivery (8) (S; U) 66. Low barrier services (8) (S; U) 67. Solution-focused brief therapy (8) ( S; U) 68. Walk-in counselling (8) (S; U) 69. Access to evidence-based psychotherapy care (8) (S; U); 70. Private practices unlinked to primary care practices (8) (S) |
| **Nine:**  **Evaluation**  **(n=61)** | 1. Multi-sectoral economic evaluation (whole system strategy) (9) (S); 2. Goal evaluation (patient-driven evaluation of goal-attainment) (9) (U); 3. Quality assurance: service providers and parents or guardians (9) (S; U; W) 4. Evidenced-based care (9) (S); 5. Evaluation (audit of relevant outcomes) (9) (S); 6. Child and family psychosocial assessment (9) (U); 7. Ongoing quality assurance (9) (S); 8. Evidence-based protocols (9) (S); 9. Evaluation of care for future care provision (9) (S); 10. Evaluation (annual and performance reporting) (9) (S; W); 11. Outcome-based reimbursement (9; 20) (W); 12. Evidenced-based interventions and behavioural strategies (9) (S); 13. Ongoing evaluation (9) (S); 14. Use of “plan-do-study-act cycles” strategy (9) (S); 15. Broad assessment of problems and needs (9) (W); 16. Patients presenting before surgery with ≥1 predefined medical comorbidity were triaged to the intensive care unit (ICU) postoperatively, while patients without severe systemic disease were triaged to a lower-acuity floor for overnight observation (9) (S; U); 17. The importance of intervening in multiple settings, and ongoing implementation and evaluation strategies (9) (S); 18. Evaluation of a new model (9) (S); 19. Measurement-based treatment models (9) (S; W); 20. “Population-based approach” to tracking outcomes (9) (S); 21. Use of evidence-based interventions (9) (S); 22. Systems to monitor and track progress (9) (S); 23. Use of evidence-based interventions (9) (S); 24. Population-based approach (9) (S; U); 25. Needs-based care (9) (S; U); 26. Evaluation: provider adherence, monitor productivity, monitor service utilization (9) (U); 27. Patient psychiatric evaluation (9) (S; U); 28. Expedite outpatient psychiatric evaluation (9) (S; U); 29. Problem-solving cycle: plan, do, check, adjust (9) (S); 30. Control rooms for assessing current performance among team members (9) (W); 31. Accepting the responsibility to identify and treat all relevant factors which can affect outcome (9) (W); 32. Evidence-based protocols (9) (S); 33. Comprehensive assessments (9) (S); 34. Evaluation (9) (S); 35. Implementation science (9) (S); 36. Implementation of family assessment and engagement tools that can be used over the long-term to monitor the health and wellbeing of family members (9; 18) (U); 37. Evidence-based protocols (9) (S); 38. Incorporating evaluation process in the mental health delivery processes: shared learning, identifying common care processes (9) (S); 39. Evidenced-based care (7; 9) (S; W); 40. Screening of risks (4; 9) (S); 41. Mechanisms for increasing treatment intensity (2; 9) (S); 42. Evidenced-based care (7; 9) (S); 43. Screening of risks (4; 9) (S; W); 44. Evaluation of patients’ psychological progress (4; 9) (S; U); 45. Development and implementation of shared assessment tools and referral criteria (1; 9) (S); 46. CPS monitoring on sight (4; 9; 13) (S); 47. Advise on measurement-based care (6; 9) (S); 48. Improved follow-up care in-depth diagnostic and family needs assessment (4; 6; 9) (S; U; W); 49. Comprehensive and up-to-date shared care plans (2; 6; 9) (W); 50. Clinical guidelines-AAP/NICHQ; templates for primary care screening tools (2; 6; 9) (W); 51. Tracking progress (1; 6; 9) (S); 52. Tracking progress (1; 6; 9; 13) (S); 53. Systematic screening and monitoring with treatment to target (4; 6; 9) (S); 54. Evidence-informed interventions (6; 7; 9) (S) 55. Measurement based (9) (S); 56. Introduction of performance measures: screening for clinical depression (9) 57. Introduction of performance measures: kindergarden readiness promotion bundle (9) (S; U; W); 58. Use of evidence-based interventions (9) (S) 59. Parent-directed early developmental screening (9) (S; U) 60. Health checks (9) (U) 61. Mental health screening (9) (S; U) |
| **Ten:**  **Effective and efficient IT systems**  **(n=48)** | 1. Co-created CYP networks (Tailored care) (10) (U); 2. Referral-discharge interface (examination of structures and processes) (10; 12) (S); 3. Social care interface (10) (S); 4. Effective information exchange between primary care, speciality care, and patients/families (10; 11) (U; W); 5. Telemedicine (10) (S); 6. Phone screening (10) (S); 7. Clinical information systems (10) (S); 8. Improving the flow of information between hospitals, specialists, community and primary care providers (10) (S); 9. Delivery of health information and services via electronic communication technologies (tele-visits) (10) (S); 10. Tele-consultations (10) (S); 11. Telehealth (10) (S); 12. Electronic scheduling platform (10) (S); 13. Report system by patients on medical journey not outcomes (tool PREM) (10) (S; U); 14. Proactive telephone outreach to socially high-risk newborns (10) (S; U); 15. Telemedicine (10) (S); 16. Creation of their own electronic health records –registry using REDCap (10) (S); 17. Effective organisational systems, support and structure (10) (S); 18. Information exchange between organisations (10) (S) 19. Telemedicine “medical reviewer NE-coordinator” which supervises patient-oriented care (10) (S; W) 20. Data sharing (10) (S; W); 21. Development of electronic tracking to prompt visits (10) (S) 22. Telehealth (10) (S); 23. The electronic medical record (10) (S); 24. Shared ICT systems (10) (S); 25. Technology-enabled collaborative care-platform for assessment (10) (S); 26. No face-to-face consultation and integrated care coordination (10) (S; U); 27. Providing up-to-date information on the availability of services (10; 11) (S; U); 28. IT tools: supporting situation understanding (10) (S); 29. Electronic records (10) (S); 30. Electronic survey (feedback from parents and teachers) (10; 18) (U; W); 31. E-mail-based platform for sharing data between parents and teachers (10; 11) (U; W); 32. sharing common electronic health records (1; 10) (S); 33. ‘Information exchange (1; 10) (W); 34. A centralized mental health telephone program (2; 10) (S; W); 35. Inclusion in the organization’s computer program of a pop-up window associated with the diagnosis of AB that automatically displays the most important documents associated with pathways (6; 10; 11) (S; W); 36. Coordinated/telephonic: increased collaboration between PCPs and behavioral health provider (1; 10) (S; W); 37. Support for remote consultation, including psychiatry and medication management (3; 10) (S; U); 38. ‘Information exchange’ (1; 10; 11) (W); 39. Electronic health record (1; 10) (S); 40. Information systems-electronic medical record, electronic prescriptions (1; 10) (S); 41. Electronic health records (1; 10) (S); 42. Electronic medical record (1; 10) (S); 43. Sharing common electronic health records (1; 10) (S: W); 44. Electronic health records (1; 10) (S); 45. Integrated electronic health record (1; 10) (S); 46. Electronic health records (1; 10) (S) 47. electronic decision support (10) (S); 48. a primary care hotline (10) (S) |
| **Eleven:**  **Communication (n=47)** | 1. Establishing communication channels (11) (U; W);   1. Facilitating communication among all healthcare- and community-based treatment team members (11) (W); 2. Communication between families (11) (U); 3. Communication between PCPs and specialists (11) (W); 4. Effective inter- and intra- service cooperation (11) (W); 5. Promotion of communication between obesity specialists and PCPs (11) (W); 6. Interdisciplinary meetings (11) (W); 7. Communication and language with service users (11) (U); 8. Telephone consultation line for pediatric medical providers across the state (11; 21; 22) (U; W); 9. Ongoing operational and clinical consultation (11) (U; W); 10. Provide integrated behavioural health consultants (11) (U; W); 11. Building cooperation in the multidisciplinary cross-sectoral team (11) (S; W); 12. Important information about families - picture of family life (11) (U); 13. High frequency face-to face contacts (11) (U; W); 14. Establishing communication channels (11) (W); 15. Feedback to front-line professionals (11) (W); 16. Responsive consultation (11) (U; W); 17. Consultation (11) (W); 18. The status sheet (11) (S; W); 19. Display of posters with the decision tree (11) (W); 20. Informal consultation (11) (U); 21. Effective communication between parents and providers (11) (U); 22. Family-driven communication tool between parents and providers (11) (U); 23. Interpersonal communication and information transfer among entities (11) (S; W); 24. Direct (with the patient & family) or indirect (case discussion) consultations (11) (U); 25. Supportive management (11) (W); 26. Negotiating Point of Independence that holistic care that supported the family unit was provided (11) (U); 27. Communication in the Context of Uncertainty (11) (U; W); 28. Holistic care that supported the family unit was provided (11) (S; U); 29. Cross-system communication (11) (S; W); 30. Motivational interviewing (11) (W); 31. Communication (11) (W); 32. Web service design to facilitate communication (11) (S); 33. Effective information exchange between primary care, speciality care, and patients/families (10; 11) (U; W); 34. Effective communication (1; 11) (S; W); 35. Strengthening culture and identity (3; 11) (S; U; W); 36. Interprofessional communication (i.e. communicating with patients, families, communities, and professionals in health and other fields in a responsive and responsible manner that supports a team approach) (1; 11) (U; W); 37. Information sharing through email (to pediatricians of the links to the BICP, highlighting the core features of the protocol for the management of the AB) (1; 11) (W); 38. Providing up-to-date information on the availability of services (10; 11) (S; U); 39. Non-hierarchical relations (1, 11, 21) (W); 40. Efficient communication between primary and mental health care providers (2; 11; 15) (W); 41. E-mail-based platform for sharing data between parents and teachers (10; 11) (U; W); 42. Inclusion in the organization’s computer program of a pop-up window associated with the diagnosis of AB that automatically displays the most important documents associated with pathways (6; 10; 11) (S; W); 43. ‘Information exchange’ (1; 10; 11) (W) 44. Setting up a learning community of professionals to exhange knowledge, experiences and tools (1; 2; 6; 7; 11; 15) (S; W) 45. Active engagement of family members or guardians (2; 4; 5; 6; 11; 18) (S; U) 46. A regional network of psychotherapy providers to facilitate the navigation of outpatient psychotherapy care with timely psychotherapy referrals and to support the collaborative care model (11) (W; S) |
| **Twelve:**  **Reduction in care fragmentation (n=51)** | 1. ‘Joined-up’ UEC system (12) (S); 2. “Bottom up” approach (12) (S); 3. Linking integrated health model to pediatric centre (decreasing fragmentation) embedded a screener and care coordinator into the practice to reduce potential time-related issues related to assessment and care coordination (12) (S; W); 4. Screening instrument (12) (S); 5. Coordination across multiple systems of care (12) (S); 6. Timeline map: “individualized Patient Pathway” (12) (U); 7. Appropriate and effective care package (12) (W); 8. Reduction of fragmentation for children through assisting child/family with communicating clinical issues (12) (U; W); 9. Continuity of care (12) (S); 10. School-based illness clinics (12) (S); 11. Coordination is driven by patient needs for the interaction on clinical management issues such as questions and concerns related to symptoms and medications (12) (S; U); 12. A multi-stepped intervention that addressed the major gaps in the referral system (12) (S); 13. “Step-down care” (12) (S; U); 14. Primary care case management (in which primary care providers receive an enhanced payment to coordinate services, and integrated managed care) (12; 20) (W); 15. Health care organization (12) (W); 16. Integration of services/reducing care fragmentation (active care coordination and management) (12) (S); 17. Coordination across multiple systems of care (12) (W); 18. Centred on working with families around their needs (12) (U); 19. Measurement-based treatment to target (12) (S); 20. Decreasing fragmentation (12) (S, U); 21. Promoting/facilitating integration of care (12) (S); 22. Interagency system change (12) (S); 23. Service integration at a systemic level, trust and favourable interpersonal relations (12) (S); 24. Comprehensive access to ambulatory-based urgent visits is associated with better coordination (12) (S); 25. Hub-and-spoke model (12) (U; W); 26. Care coordination (12) (S); 27. Information sharing (detailed information exchange between organisations/staff members) (12) (S); 28. Active care coordination and management (12) (W); 29. Systematic approach to newborn care (12) (S) 30. Focus on the whole patient (12) (U); 31. Case management (12) (W); 32. Standardized perioperative (preoperative, intraoperative, and postoperative) protocols were developed, with a focus on preoperative risk stratification (12) (S); 33. Coordination across multiple systems of care (12) (W); 34. Coordination across multiple systems of care (12) (S; W); 35. Clinical restructuring of care (12) (S); 36. Care plan – road map (12) (S; U); 37. Goal setting and problem-solving (12) (W); 38. Reminders and close contact with case manager appreciated (12, 16) (W); 39. Care coordination (12) (W); 40. Care coordination (12) (S; W); 41. Referral-discharge interface (examination of structures and processes) (10; 12) (S); 42. ‘Preconditions’ (8; 12); 43. Coordinated/telephonic models to fully integrated models (i.e. behavioral health care delivered in clinic with significant collaboration and coordination of treatment plan between behavioural health clinician and other sites) (1; 12; 13; 15) (S); 44. Development of care coordination measurement instrument (identity record and encounter record) (1; 12) (W); 45. Create work flow analyses prior to implementation (2; 6; 12) (S); 46. Thinking systemically (2; 7; 12; 15) (W) 47. Care provision through intersectorial teams (1; 2; 12; 15) (S; W); 48. Establshing basic structures for cooperation across interdisciplinary teams (1; 2; 12; 15) (S; W); 49. Integrates mental health services in general services (1; 4; 6; 12) (S; W); 50. Service integration (12) (S) 51. A regional network of psychotherapy providers to facilitate the navigation of outpatient psychotherapy care with timely psychotherapy referrals and to support the collaborative care model (11; 12; 13; 15) (W; S) |
| **Thirteen:**  **Continuity of care**  **(n=46)** | 1. Family preparedness for transitioning to home (13) (U); 2. Effective discharge planning procedures (13) (W); 3. The formation of partnerships between primary care clinicians and child and adolescent psychiatrists (13) (W); 4. Supporting and revising care plans (longitudinal plans toward transition from specialty back to primary care and pediatric to adult care) (13) (W); 5. Ensuring successful linkages with community-based resources (13) (W); 6. Intake visit (13) (W); 7. Continuity of care (13) (S); 8. Ongoing follow-up (13) (U; W); 9. Improved receipt of preventive care leads to continuity of care (13) (W); 10. Acute and chronic care management (13) (S; W); 11. (Two-generation approaches) - transformative models (13) (S); 12. Gradual transition model: taking time for patient care (13) (S); 13. Frequent return visits (13) (S; U); 14. On-going follow-up (13) (U; W); 15. Continuity of staff/leadership (13) (W); 16. Referral model (13) (S; W); 17. Improved discharge processes (13) (S); 18. Restoring weight and providing health maintenance and follow-up (13) (U); 19. Monitoring and managing medical complications and making referrals and coordinating an interdisciplinary team (13) (S; W); 20. Lifelong child-, youth- and family-driven care and supports that optimize health and quality of life (13) (S; U); 21. Continuity in care from paediatric to adult health care (13) (S; U); 22. Continuity of care (13) (S); 23. Revision of health history from birth (13) (U); 24. Continuous care, and fewer appointments (13) (S); 25. Intake/treatment (13) (U; W); 26. Timely support across several life domains tailored to a family’s needs (13) (U); 27. Routine follow-up (13) (S); 28. Use of up-to-date communication devices and tools valued (13) (W); 29. Provide resources and information (13) (S); 30. Rapid follow-up to care (13) (S); 31. Continuity-of-care approach (13) (S); 32. Routine follow-up (13) (S); 33. Service supervision (13) (W); 34. Integrated psychology trainees in a safety net hospital's pediatric ED and urgent care (4; 13) (W); 35. Formation of partnerships between schools, families, and community health-care providers (2; 13) (S); 36. Off-site mental health provision (8; 13) (S; U; W); 37. Families: patient journey map (3; 13) (U); 38. Co-designing innovation requires sustained commitment long-term (2; 13; 19) (U); 39. Coherent, continuous, and coordinated care (1; 13) (S; W); 40. CPS monitoring on sight (4; 9; 13) (S); 41. Coordinated/telephonic models to fully integrated models (i.e. behavioral health care delivered in clinic with significant collaboration and coordination of treatment plan between behavioural health clinician and other sites) (1; 12; 13; 15) (S); 42. Referral (1; 2; 4; 13) (S; U; W); 43. Tracking progress (1; 6; 9; 13) (S); 44. Tailored care to children and youth who need it most (13) (S; U) 45. Follow-up care (13) (S; U); 46. A regional network of psychotherapy providers to facilitate the navigation of outpatient psychotherapy care with timely psychotherapy referrals and to support the collaborative care model (11; 12; 13) (W; S) |
| **Fourteen:**  **Network/team**  **(n=32)** | 1. Confluence of physical and mental health (14) (S); 2. Co-location (Located in the same physical space) (14) (S); 3. Tele-visits overcome the barriers of co-location (virtual co-location) (14) (S); 4. Co-located services in the pediatric primary care office (14) (S); 5. Resource mapping; training for primary care providers, and a collocated MSW intern training program (14) (S; W); 6. Assessment through A5 application: "assess-advise-agree-assist-arrange"-"Gather info"-"Screening"-"Telephonic outreach " - "Change motivation" (14) (S); 7. MD BHIPP has four components: a child psychiatry access program (CPAP) (14) (S); 8. Co-location (14) (S); 9. Interprofessional approaches (14) (S; W); 10. Co-location in certain services (14) (S); 11. ‘Interprofessional collaboration’ (14) (W); 12. Co-located: Located in the same physical space (ideally leading to an ease in referral and patient comfort) (14) (S; U; W); 13. Co-location of services (14) (S); 14. Institutionalizing interprofessional collaboration via higher levels of integration (with co-located mental health providers systematically incorporated into routine medical care and PCPs/co-located providers developing joint treatment plans) (14) (S; W); 15. Co-located care (14) (S); 16. Meaningful engagement of staff (14) (W); 17. Effective team working (14) (W); 18. Co-location of multidisciplinary and/or interagency staff and cultivating faith in positive change among staff (14) (W); 19. Co-location (e.g. a separate behavioural health provider receives a facilitated referral to treat a patient from a partnered primary care practice down the hall) (14) (S); 20. Co-location of multidisciplinary and/or interagency staff (1, 14) (S); 21. Co-location (e.g., a separate behavioural health provider receives a facilitated referral to treat a patient from a partnered primary care practice down the hall) (1; 14) (S); 22. Brief interventions onsite at the primary care clinic (4; 14) (U; W); 23. Potential co-joint treatment planning (2; 14) (S; U); 24. Interprofessional collaboration (2; 14) (W); 25. One treatment environment (‘one stop shop’) (8; 14) (S; U); 26. Common care culture (1; 14) (W); 27. Co-location in community health centres (1; 8; 14) (S); 28. Co-location in community health centres (1; 8; 14) (S) 29. Co-location of services in one place including physical and sexual health, mental health, substance use, peer support, social services (14) (S) 30. co-located services (14) (S); 31. collaborative care; psychiatrist on as-needed basis (14; 16) (S) 32. Psychiatrist on as-needed basis (14) (S) |
| **Fifteen:**  **Co-Location**  **(n=31)** | 1. The team is responsible for care delivery (15) (W); 2. Teams and teamwork (15) (W); 3. Team-based care (15) (W); 4. Coordinated services by a team of professionals (family partners and peer supports can also be an integral part of the care team) (15) (U; W); 5. Building connections (15) (U; W); 6. Safe network (15) (W); 7. Strong and complementary, interagency connections and partnerships (15) (W); 8. Relational inter-professional teamwork (15) (W); 9. Multi-disciplinary teams (15) (W); 10. Team-based care (15) (W); 11. Teams and teamwork (i.e. applying relationship-building values and the principles of team dynamics to perform effectively in different team roles (15) (W); 12. Badges for uniform “team on the pathway” (15; 23) (W); 13. Care networking (15) (W); 14. Care networking (15) (W); 15. Collaboration: Team driven (15) (W); 16. Teamwork -> effective teamwork have been identified by researchers in both health care and education (15) (W); 17. Coordination (15) (W); 18. Self-actualization (15) (U); 19. Teams and teamwork (15) (W); 20. Reliable team interactions between physicians and staff (1; 15) (W); 21. Developing multi-stakeholder partnerships (1; 15) (W); 22. Developing strong and enduring partnerships (2; 15) (W); 23. Efficient communication between primary and mental health care providers (2; 11; 15) (W); 24. Thinking systemically (2; 7; 12; 15) (W); 25. Coordinated/telephonic models to fully integrated models (i.e. behavioral health care delivered in clinic with significant collaboration and coordination of treatment plan between behavioural health clinician and other sites) (1; 12; 13; 15) (S) 26. Setting up a learning community of professionals to exhange knowledge, experiences and tools (1; 2; 6; 7; 11; 15) (S; W) 27. Care provision through intersectorial teams (1; 2; 12; 15) (S; W) 28. Establsihing basic structures for cooperation across interdisciplinary teams (1; 2; 12; 15) (S; W) 29. Features that include team-based care (15) (W); 30. Staff collaboration (15) (W) 31. A regional network of psychotherapy providers to facilitate the navigation of outpatient psychotherapy care with timely psychotherapy referrals and to support the collaborative care model (11; 12; 13; 15) (W; S) |
| **Sixteen:**  **Designated coordinator/ navigator/ key worker**  **(n=30)** | 1. Key worker delivered individually tailored family support and education (16) (W); 2. Single point of contact that communicates and coordinates with patients, families, clinicians, and services (16) (U); 3. Care coordination: leadership and key coordinator (16) (W); 4. A central role of nurses (16) (W); 5. Provision of all patients with a dedicated oncologist (16) (S; U); 6. Designated Coordinator/Navigator/Key Worker/Case Manager: help guide patients/caregivers navigate through the healthcare system (16) (W); 7. Trusted member of the community (16; 19) (W); 8. Two new introduced specialists: a cross-appointed nurse practitioner and life skills coach (16) (W); 9. Each child is assigned a case worker (16) (U); 10. Centralized care with access to a single person coordinating clinic visits (16) (S; W); 11. Care managers (16) (W); 12. ‘Expertise’ (Expertise Knowledge and training, Extending knowledge by means of training, StrongGuidelines, The use of evidence-based guidelines to support professionals, StrongSelf-efficacy, Confidence and comfort of professionals to provide integrated care) (16) (W); 13. Case manager is the link among networks (16) (W); 14. Case manager is the single point of contact (16) (W); 15. The importance of the case manager’s role (16) (W); 16. Importance of relationships with key professional who coordinates care (16) (W); 17. Need for a psychologist as an integral member of the healthcare team; electronic medical record (16) (W); 18. Key manager (16) (W); 19. A neutral chairperson (16) (U); 20. Support through networking, education and partnership (1; 16) (W); 21. A tertiary-care-based nurse practitioner (7; 16) (W); 22. Reminders and close contact with case manager appreciated (12, 16) (W); 23. The key health-worker understood the health needs of the family in context (6; 16) (U; W); 24. Care managers to facilitate IC Foreign language specialists (Spanish –speaking SWs) (3; 16; 24) (W); 25. Dedicated case management or navigation to help families access and engage with mental health care (5; 16) (W); 26. Nurse as a coordinated professional to provide stepped and matched care (16), (W; S) 27. Coordinated professionals (16) (W; S) 28. Centralised model (16) (W; S) 29. Psychiatrist on as-needed basis (14; 16) (S) 30. Implementing nurse-led BH response teams to assist staff with psychiatric patient escalations (16) (W; S) |
| **Seventeen:**  **Leadership**  **(n=26)** | 1. Leadership (community lead, family lead, clinician lead) (17) (W); 2. Leadership (community lead, family lead, clinician lead) (17) (W); 3. Leadership buy-in (17) (W); 4. Preparedness to share power and information (17) (W); 5. Interprofessional leadership (17) (W); 6. Leadership (community lead, family lead, clinician lead (17) (W); 7. Strategic planning (17) (W); 8. Leadership is in relation to the management of budgets, and the ability to realise financial (rather than just structural) integration health and social care integration (17) (S); 9. Health and social care integration (17) (S); 10. Leadership (community lead, family lead, clinitian lead (17) (W); 11. Leadership (community lead, family lead, clinician lead) (17) (W); 12. The need for a ‘champion’ (17; 23) (W); 13. Clear professional roles and responsibilities (17) (W); 14. Leaders supporting interprofessional collaboration in the organisation (17) (W); 15. Strong and supportive management (17) (W); 16. Leadership: leaders supporting interprofessional collaboration in the organisation (17) (W); 17. “Champion” within the system (17) (W); 18. Structural leadership (17) (W); 19. Implementation leadership-implementation citizenship behaviour (going beyond the call of duty to support implementation) (17) (S; W); 20. ‘Professional identity’ (Professional roles and responsibilities, Clarity and expectations about professional roles, sharing responsibility, StrongAttitudes, Attitudes and commitment towards integrated care and collaboration, StrongShared thinking, A shared foundation in thoughts, aims, priorities, and values, Strong—very strong; trust, respect and equality; mutual trust, respect for other professionals and perceived equality, Strong) (17) (W); 21. The need for a ‘champion’ (17; 23) (W); 22. Effective leadership (17) (W); 23. Collaborative leadership: building shared meaning (17) (W); 24. Family paediatrician as a key figure coordinating care (17) (U; W); 25. The engagement of clinical and professional leads in the process (1, 17) (W); 26. Shared leadership (1; 17) (W) |
| **Eighteen:**  **Family engagement (n=26)** | 1. Co-creation of care with parents (18) (U); 2. Involving the voices of young people (18) (U); 3. Youth partners engaged to bring “patient” voice (18) (U); 4. Involving families (18) (U); 5. A whole of family care approach (18) (U); 6. Partnership with children, their family and multidisciplinary providers (18) (U); 7. Engaging community (18) (U); 8. Client education and engagement (18) (U); 9. Providers: focus-group methodology (18) (S); 10. Involving patients and their families in quality teams (18) (U); 11. Consumer engagement is essential to implement sustainable healthcare interventions (18) (U); 12. Cultural competency (18) (S); 13. Participants used workarounds to adapt and overcome limitations in their information environment (18) (U); 14. Active care (1, 18) (S; W); 15. Youth as co-investigators and co-creators of the initiative (3; 18) (U); 16. The implementation of an integrated youth team through strategic, tactical and operational control rooms to offer coordinated services (the central component of this integration project) (2; 18) (S); 17. Parental involvement in decision making processes (3; 18) (U); 18. Staff/family partnership (2; 18) (U; W); 19. Child-appropriate language (8; 18) (U); 20. Professionals involved children and caregivers in treatment (3; 18; 21) (U; W); 21. Electronic survey (feedback from parents and teachers) (10; 18) (U; W); 22. Leveraging other family, social, and organisational relationships (2; 18) (U); 23. Implementation of family assessment and engagement tools that can be used over the long-term to monitor the health and wellbeing of family members (9; 18) (U) 24. Active engagement of family members or guardians (2; 4; 5; 6; 11; 18) (S; U) 25. Risk-prevention psychoeducation for guardians of individuals with suicide-related emergencies (2; 4; 5; 6; 18) (S; U); 26. Risk -targeted follow-up for caregivers of individuals with suicide-related emergencies (2; 4; 5; 6; 18) (S; U) |
| **Nineteen:**  **Safety and security**  **(n=23)** | 1. Robust clinical governance, quality and safety policies (19) (S); 2. Privacy to parents and families (19) (S; U); 3. The terms and conditions of existing staff in newly integrated structures (19) (W); 4. Safe and youth friendly environment (19) (S; U); 5. Recreational and hang-out space (19) (S; U); 6. Ensure privacy (19) (S; U); 7. Governance and workload balance (19) (S; W); 8. Insurance (19) (S); 9. Sustainability (19) (S); 10. Ethical guidance considerations: The very act of delivering team-based interprofessional care will inevitably create conflicts between different guild-specific ethical guidelines (19) (S); 11. Ensure privacy (19) (U); 12. Privacy of family members during information exchange (19) (U); 13. Initial distrust of intrusion (19) (U); 14. Time facilitates personal disclosures (19) (U); 15. Feeling safe and trusted (19) (U; W); 16. Guidelines regarding confidential information (7; 19) (S); 17. Guidelines regarding confidential information (7; 19) (S); 18. Trusted member of the community (16; 19) (W); 19. Trauma-informed care (7; 19) (S); 20. Co-designing innovation requires sustained commitment long-term (2; 13; 19) (U) 21. Creating commitment (by presentations, conferences, etc) (8; 19) (S; U); 22. Increased hospital security (19) (S) 23. Constant observer care workforce (19) (S) |
| **Twenty:**  **Finance/ budgeting (n=16)** | 1. Payment and performance measurement based on child health outcomes (20) (S); 2. Value-based payment models (20) (S); 3. Fundraising (lack of resources) (20) (S); 4. Value-based payment models (defined as those in which providers are paid based on patient outcomes) (20) (S); 5. Implementation of cost measures: a ‘net present value of care’ measure that includes actual short term healthcare savings and predicted savings over a specified set of years on the basis of intermediate health outcomes achieved (20) (S); 6. Investment of resources from partnering organisations (20) (S); 7. Re-structuring existing services rather than building new (20) (S); 8. Finance: develop financially sustainable models for integrated primary care (20) (S); 9. Funding (20) (S); 10. Therapists funding:-insurance coverage (20) (W); 11. Emphasis on finances that need to be assessed as total inflow minus total outflow (20) (S); 12. Effective health and multi-agency agreements and funding arrangements (2; 20) (S); 13. Primary care case management (in which primary care providers receive an enhanced payment to coordinate services, and integrated managed care) (12; 20) (W); 14. Outcome-based reimbursement (9; 20) (W); 15. Shared governance and investment are essential to effect and sustain inter-agency change (1; 20) (S; W); 16. Material resources that flow (or not) from being heard that professionals involved children and caregivers in treatment (3; 20) (U) |
| **Twenty one:**  **Empowering staff**  **(n=15)** | 1. Developing new ways of working (21) (S); 2. Motivation of professionals (21) (W); 3. Tailored to the developmental needs of children (21) (S; U; W); 4. Voice and equal participation to all team members (21) (W); 5. Clear clinical pathways (21) (S; U); 6. Culturally tailored, clearly defined roles (21) (S; W); 7. Staff and organizational capacity building (21) (W); 8. Health care capacity (21) (S); 9. Office space in primary care practice (21; 25) (W); 10. Telephone consultation line for pediatric medical providers across the state (11; 21; 22) (U; W); 11. Involvement of staff in the development of policies and procedures (7; 21) (W); 12. Roles and responsibilities (i.e., using the knowledge of one’s own role and those of other professions to appropriately assess and address healthcare needs) (7; 21) (S; W); 13. 3rd model: embedded specialists (1; 2; 21) (W); 14. Non-hierarchical relations (1, 11, 21) (W); 15. Professionals involved children and caregivers in treatment (3; 18; 21) (U; W) |
| **Twenty two:**  **Qualification/ expertise (n=9)** | 1. Qualification of staff (22) (W); 2. Experienced and skilful clinicians (22) (W); 3. Complementary expertise (22) (W); 4. ‘Expertise’ (22) (W); 5. High quality service (22) (W); 6. Onsite mental health professional (22) (W); 7. The presence of specialist practitioners (22) (W); 8. Bringing pediatric psychology to the “front lines” of community care (2; 6; 22); (W); 9. Telephone consultation line for pediatric medical providers across the state (11; 21; 22) (U; W) |
| **Twenty three: Professional identity**  **(n=12)** | 1. The need for ‘champions’ (23) (W); 2. Role clarity (23) (W); 3. Knowledge of roles of each service (23) (W); 4. Tailored and individualised goals (23) (S); 5. ‘Professional identity’ (23) (W); 6. Feeling safe and trusted (23) (U); 7. Scope of the familiar emergency response role (5; 23) (W); 8. The CCM delivery system design (The CCM model urges practices to define roles for members of the care team with doctors and other highly trained staff addressing acute problems, and training other team members who do more patient engagement and routine work) (7; 23) (S); 9. The need for a ‘champion’ (17; 23) (W); 10. Badges for uniform “team on the pathway” (15; 23) (W); 11. Clear allocation of tasks and responsibilities (6; 23) (W); 12. The need for a ‘champion’ (17; 23) (W); |
| **Twenty four: Role of language and culture**  **(n=9)** | 1. Enhanced professional networks (24) (W); 2. Clearly defined goals (24) (W); 3. A defined scope of services (24) (S); 4. Additional training and support to non-English speaking families (24) (U); 5. Communication (rising awareness among hospital teams) (24) (W); 6. Clearly defined goals (24) (W); 7. Language (24) (W); 8. Clarifying the right diagnosis for youths (3; 24) (U; W); 9. Care managers to facilitate IC Foreign language specialists (Spanish –speaking SWs) (3; 16; 24) (W) |
| **Twenty five:**  **Facilities**  **(n=4)** | 1. Established infrastructure (25) (S; W); 2. Challenges of Working Alongside a Stretched Workforce (25) (W); 3. Office space in primary care practice (21; 25) (W); 4. Provision of necessary resources for the child and family (8; 25) (U); |

**Table S5: Brief description of the 25 Component Themes of integration identified in this review.**

| **Component Theme (n=25)** | **Brief description of Component Theme** |
| --- | --- |
| 1. **Shared professional responsibility and practices** | The inteded targets of impact of the ‘Shared professional responsibility and practices’ were the workforce and the system.  This Component Theme was mainly focussed on changing or improving the organisational culture and structure towards multidisciplinary teams, collective responsibility, mutual knowledge exchange and shared care records. Subcomponents which shifted ways of working towards shared practices included shared caseload, assessment tools and shared referral criteria; shared decision-making processes and responsibilities among providers; prioritising shared goals via a team approach. It is noteworthy that, although shared caseload was mentioned as an important subcomponent, a clear division of responsibilities as well as decentralised decision making were also part of shared responsibility. Some underpinning principles which are suggested in order to achieve shared professional responsibility and practices in the context of integration include maintenance of a sense of common purpose and non-hierarchical relationships.  The importance of mutual/relationship trust in shared professional responsibility and practices was highlighted in four subcomponents. |
| 1. **Stronger connections and partnerships** | This intended target of impact of this Compoment Theme was the system, users and workforce.  Collective impact, meaningful involvement and a spirit of mutual professional understanding were seen as desirable outcomes which could be achieved through the formation of partnerships between multidisciplinary teams, families, schools and other sectors/agencies, and communities. Involvement of multidisciplinary teams, partnering with young people, mutual commitment and willingness to change, cross-organisational relationships, consistent communication, widely advocated norms of reciprocity and adaptability, face-to-face interactions and a sense of obligation among group members all contributed to building strong connections and partnerships.  The importance of high levels of trust in building strong partnership/relationships was highlighted in four subcomponents. |
| 1. **Empowerment of service users** | The ‘Empowerment of Service Users’ Component Theme intended to target service users by changing organisational processes.  Meeting families and carers on their own terms, using a child-centred approach, building families agency, creating a safe and nurturing environment, creating an open and respectful atmosphere to reduce power imbalances, respect for the young person’s autonomy, developing transparent care plans that were culturally approriate, being mindful of health literacy and contextualising materials to youth culture, and preparedness to share information and power, were all intended to shape the empowering ethos of integrated care. The location of services was also identified as an important subcomponent. For example, meeting in a familiar setting, meeting youth at a location of their choice, providing a young adult clinic, and providing a comprehensive support system for nursery schools. Valuing the voice of CYPF was identified as an important subcomponent, for example ’Feeling heard and receiving contextualized support’.  The importance of building trust was highlighted in two subcomponents with one recogising that trust is built on the recognition of professional learning and enhanced through physical presence. |
| 1. **Early detection and prevention** | The ’Early Detection and Prevention’ Component Theme reflected a shift towards prevention rather than a diagnosis-driven approach to healthcare. The intended targets of impact for this were the system, users and workforce.  The preventative components included a range of early assessment tools such as (1) early screening and routine screening of risk factors; (2) reinforcement of anticipatory guidance; (3) provision of close family supervision; (4) staff training (diagnosis-specific training); (5) development of identity records and (6) early identification of severe injuries. The impetus to develop preventative components have been triggered by health inequalities in communities often leading children to be more prone to suffering from chronic diseases, exhibiting psychological or social and/or behavioural difficulties, suffering from injuries or at risk of crisis.  Subcomponents that targeted the system included ’’Health sector collaborate with other sectors’, ’Integrated in community’ and ’Implementation of a cross-age and interdisciplinary mobile early detection team’. Subcomponents that targeted users included ’Medication management’, ’symptonm awareness’ and ‘safeguarding’. Subcomponents that targeted the workforce included ’Early diagnostics (school-based assessment and school-based intervention)’, ’proactive and preventive assessment of prenatal problems’ and ’”Back up” of psychiatrists services’. |
| 1. **Training of parents** | The ’Training of Parents’ Component Theme was targeted at the system, users and workforce, not just users.  Subcomponents that targeted the system focussed on individual care plans and record systems, capacity building, and in one study a ’No drop-out’ policy.  For parents and careers (users), the focus of the training was on education to support self-management and safe transitions of care, particularly for chronic health conditions. One study described this training in term of ’parents acting as active agents, so called family partners. The training, in some studies, led the active involvement in the treatment of their child which included co-development and co-design of the treatment plan. One study highlight that this training should involve culturally appropriate care. For the workforce, a family-centred approach and individualised care plans were at the centre of the training which provided warm-handoffs for direct patient engagement. One study highlighted training around ’play therapy and preoperative teaching for families’ and another the usefulness of ’collaboration through learning’. |
| 1. **Awareness** | The ’Awareness’ Component Theme was targeted at the system, users and workforce.  For the system this included an awareness for ’Addressing the social determinants of health’ and ’Equality’, providing ’Evidence-informed interventions’ / evidence-based care and factoring ’Time to address a broad spectrum of problems for inter-professional collaboration’. For the workforce, subcomponents included ’the key health-worker understood the health needs of the family in context’ and ’Emphasising patient-reported outcomes’ alongside awareness of clinical guidelines and care protocols. One study identified ’Bringing pediatric psychology to the “front lines” of community care’. For service users, ’Symptom awareness’, risk prevention and screening were highlighted. This was particularly true for studies that focussed on mental health and the prevention of suicide; ‘Risk-prevention psychoeducation for guardians of individuals with suicide-related emergencies’ and ’Risk-targeted follow-up for caregivers of individuals with suicide-related emergencies’. |
| 1. **Workforce development** | The ’Workforce Development’ Component Theme targeted the system and workforce. Most of the subcomponents focussed training of various types, for all or certain members of the workforce. Studies acknowledged that this would require an investment in resources (including time). A number of studies highlighted the need for this training to include a culture of evaluation and improvement. One study had implemented four-year trialogue interventions to improve mental health literacy, stigma and service utilization (a trialogue ‘awareness campaign’ including cinema spots, city-light posters). Knowledge transfer (individuals with different professional backgrounds and knowledge and skills working together) and setting up a learning community of professionals to exhange knowledge, experiences and tools were subcomponents.  One study highlight the importance of trusting peer-led training. |
| 1. **Accessibility and availability** | This Component Theme was targeted at the system. Common subcomponents included community-based services, location and co-location of services, ’flexibility and adaptability of services including home visiting’ and ’Walk-in sessions’. Examples included ’One step access to walk-in services’, ’Extended hours of operation and cost free’, ’Multiple entry points (self-referral, drop-in)’, ’One treatment environment (‘one stop shop’)’ and ’One-stop health and social service delivery’. The availability of healthcare professions was also deemed important with a shift towards short waiting times and the limited need for assessment forms to fill out being desirable.  One study highlighted that high levels of trust between parties was important for this Component Theme. |
| 1. **Evaluation** | The ’Evaluation’ Component Themes targeted the system. The type and focus of evaluations varied between different studies; economic evaluations, goal evaluations, audit of relevant outcomes, quality assurance, screening of risks, performance measures, implementation evaluations, provider adherence and service utilisation. One study used a ’plan-do-study-act cycles’ strategy for evaluation and another used a ’Problem-solving cycle: plan, do, check, adjust’. One study highlighted the importance of ’intervening in multiple settings, and ongoing implementation and evaluation strategies’. |
| 1. **Effective and efficient IT systems** | The ’ Effective and efficient IT systems’ Component Theme targeted the system. The majority of subcomponents focussed on effective data sharing and information exchange systems at the interface of one part of the health service with another, or another sector including schools and social care. Systems included IT, phone, telemedicine including tele-visits and tele-consultations, and other types of electronic information systems. Examples included ’A centralized mental health telephone program’, ’Inclusion in the organization’s computer program of a pop-up window associated with the diagnosis of AB that automatically displays the most important documents associated with pathways’ and ’Coordinated telephonic service to increase collaboration between primary care providers and behavioral health provider’.  Introduction of new developments in IT systems were seen to play a significant role in improving outreach to families and socially high-risk populations. Also, regarding telemedicine, it was seen as helping to overcome the existing barriers relating to challenges with co-location. Other developments such as electronic tracking and shared common electronic health records were intended to improve the flow of information between hospitals, specialists, community and primary care providers. |
| 1. **Communication** | The target of the ’Communication’ Component Theme was the system, users and workforke. Subcomponents included techniques and practicies which aimed to establish smooth and effective information exchange and between all healthcare and community-based team members and service users. Examples included ’Communication in the context of uncertainty’, ’Web service design to facilitate communication’ and ’Holistic care that supported the family unit was provided’.  Of note, this Component Theme was ranked higher for integrated care systems and models that focussed on CYP living with learning disabilities & autism or obesity compared with all studies. |
| 1. **Reduction of care fragmentation** | The ’Reduction of Care Fragmentation’ targeted the system and workforce.  The system changes were focussed on coordination across multiple systems of care and included the introduction of a ’bottom-up’ approach, joined-up care, ’step-down care’, ’clinical restructuring of care’ and ’a multi-stepped intervention that addressed the major gaps in the referral system’. One study specifically mentioned ’Interagency system change’. Service integration at a systems level was a common theme and one study highlighted the importance of trust and favourable interpersonal relations in this regard.  Subcomponents that targeted the workforce included ’active care coordination and management’ and ’Coordination across multiple systems of care’. |
| 1. **Continuity of care** | The ’Continuity of Care’ Component Theme was closely linked with ”The reduction of ’Fragmentation of care’ component theme. The underlying difference in the two Component Themes was that whilst reduction of ’Fragmentation of care’ focused on linking various aspects of care into one uninterrupted treatment journey, ’Continuity of care’ focussed on ensuring that care and support would carry on through that journey and beyond ‘the treatment walls’, particularly after discharge from hospital. Examples included ’Family preparedness for transitioning to home’, ’Effective discharge planning procedures’, ’Ensuring successful linkages with community-based resources’, ’Formation of partnerships between schools, families, and community health-care providers’, ’Ongoing follow-up’ and also ’Continuity in care from paediatric to adult health care’.  The intended targets of impact of this Component Theme was the system, users and workforce. |
| 1. **Co-location** | The ’Co-location’ Component Theme focussed locating different member of the multidisciplinary team and inter-agency staff in the same physical or virtual space where the intended targets of impact were the system and the workforce. One study suggested that this ’ideally leads to an ease in referral and patient comfort’ and another suggested this leads to ’potential co-joint treatment planning’. The underlying principle of cco-location in this context was creating one treatment environment (a ‘one stop shop’) with a ’common care culture’. One example involved co-location of services in one place that included physical and sexual health, mental health, substance use, peer support and social services. |
| 1. **Network / team** | The main intended target of impact for this Component theme was the workforce - developing and building strong and enduring networks or teams, and partnerships, across different sectors. For example ’Strong and complementary interagency connections and partnerships’ and ’applying relationship-building values and the principles of team dynamics to perform effectively in different team roles’. The importance of these networks being ’safe’ and ’reliable’ was highlighted by some subcomponents. One study mentioned that ’family partners and peer supports can also be an integral part of the team’. One study suggested the use of badges for uniforms containing the slogan ’team on the pathway’. |
| 1. **Designated coordinator/ navigator/ key worker** | The ’Designated coordinator/navigator/key worker’ Component Theme was built around the practice of care coordination by a dedicated coordinator to enable service users to navigate the integrated care system efficiently and effectively. Different to a leadership role, the introduction of this single point of contact was suggested as being an important point of contact between service users, staff, services and networks. One study highlighted that this single point of contact should operate as a ’Trusted member of the community’. Another study highlighted the importance of staff in this role having extended knowledge by means of training, self-efficacy and confidence. Another study highlighted the importance for the key health-worker to understood the health needs of the family in context. |
| 1. **Leadership** | The subcomponents of the ’Leadership’ Component Theme focused on critical characteristics and features that constituted a good and strong leader or a leadership team. However, the subcomponents often contradicted one another depending on the type of integrated care model, health condition, and other factors. In some studies, leadership was seen as a group of leaders who support interprofessional collaboration, with non-hierarchical relations, sharing managerial responsibilities and power across the team with an adaptive leadership style. Other studies favoured one central figure, a so-called ‘champion’ or a neutral ‘chairperson’, who would coordinate all processes.  Interprofessional support and collaboration alongside a willingness to share thinking, information, power and strategic planning were seen as underpinning principles of effective leaders or leadership teams. One study highlighted that strong leadership required mutual trust, respect for other professionals and perceived equality. |
| 1. **Family engagement** | The ”Family Engagement” Component Theme focussed on CYPF involvement and engagement; most subcomponents focussed on engagement rather than involvement. The intended target of most subcomponents was the service users. Examples of subcomponents that focussed on engagement where the intended target of impact was the service user include ’Consumer engagement is essential to implement sustainable healthcare interventions’, ‘Involving the voices of young people’ and ’Youth partners engaged to bring the “patient” voice’. Examples of CYPF involvement included ’Co-creation of care with parents’, ’Youth as co-investigators and co-creators of the initiative’, ’Parental involvement in decision making processes’ and ’Professionals involved children and caregivers in treatment’. The use of ’Child-appropriate language’ was highlighted in one study.  A few components targeted the workforce and examples include providing ’active care’ or ’active engagement’. Others targeted the system and included ’Cultural competency’ and ’The implementation of an integrated youth team through strategic, tactical and operational control rooms to offer coordinated services’. |
| 1. **Safety and security** | Ensuring safety, trust and privacy of service users and their family members during information exchange were highlighted as important components when it came to safety and security provision of service users. This was often listed in the context of good governance, ethics, clinical guidelines and policies. The intended targets of impact were users and the system.  This Component Theme also included subcomponents that focused on safe and secure social spaces for young people; 'Safe and youth friendly environment’ and ’Recreational and hang-out space’. Three subcomponents specifically mentioned ’trust’. One focused on ’Feeling safe and trusted’, for both staff and CYPF, one on ’Initial distrust of intrusion’ and one on ’Trusted member of the community’. |
| 1. **Finance/ budgeting** | The ”Finance/Budgeting” Component Theme included a number of subcomponents that focussed on the challenges of funding and particularly the challenges of shared budgets between agencies, for example ‘Effective health and multi-agency agreements and funding arrangements’, ’Shared governance and investment are essential to effect and sustain inter-agency change’ and ’Emphasis on finances that need to be assessed as total inflow minus total outflow’. The intended target of impact was the system. The importance of developing ’financially sustainable models for integrated primary care’ and ’Investment of resources from partnering organisations’ were also highlighted. A number of other subcomponents focused on different payment models including reimbursement. |
| 1. **Empowering of Staff** | The ’Empowerment of Staff’ Component Theme included a range of subcomponents which promoted equality across the workforce. The intended targets of impact of this Component Theme were the workforce, users and the system. The establishment of horizontal organisational structure (instead of hierachical), awareness of everyones’ roles and responsibilities, availability of training and development opportunities, celebrating cultural diversity and opportinities to hear staff ’voices’ all contributed to staff empowerment. Subcomponents such as ’development of new ways of working’ and ’clear clinical pathways’ targeted at the system were viewed as allowing staff to increase their capacity and capability within their roles. Encouraging staff to provide services that were ’Tailored to the developmental needs of children’ and being ’involved in the treatment of CYPF’ were intended to benefit the service users. |
| 1. **Qualification/ expertise** | The ”Qualification/expertise” Component Theme had a focus on the importance of specialist training and the need for clinicians to have appropriate experience and skills. Closely linked to the ’Workforce development’ Component Theme, staff with approriate qualifications and expertise were viewed as playing an important role throughout the integrated care journey for CYPF. Mental health specialists were highlighted as critical to the success of integrated care for this health condition and should be embedded into primary and community care. |
| 1. **Professional identity** | The ”Professional identity” Component Theme focussed on clarity of roles and responsibilities, the feeling of security and safety in these roles and the need for ”champions” of integrated care. The clarity in roles and responsibilities enabled staff to shape their professional identity and overcome a sense of uncertainty in relation to their duties and the changing structure. Here, it is suggested that a significant role is played by the care coordinator who should be responsible for navigating the staff and patients through the system. One study highlighted the role that professional identity plays in service users ’feeling safe and trusted’. |
| 1. **Role of language and culture** | ’The Role of Language’ was seen as an intersectional component targeting CYPF as well as staff working across different sectors and systems that provide care and support. The intended targets of impact were the workforce and the system.  For CYPF, subcomponents such as “child-appropriate language”, “child-friendly counselling service” and “communication through accessible language” were intended to help to meet CYPF on their terms, provide a child-friendly environment, encourage engagement between service providers and CYPF families, and help empower CYPF. By addressing the inequalities for CYPF where English was not their first language, subcomponents such as “culturally competent, accessible language”, “additional training and support for non-English speaking families” was seen as an important mechanism in the empowerment of CYPF families and promotion of accessible care.  Fot staff, an approach to make workforce terminology more universal (and avoid jargon) across different roles and sectors was intended to help promote a better understanding of everyone’s roles and responsibilities as well as increase a sense of a community and one-team approach. |
| 1. **Facilities** | This was the least commonly identified Component Theme. It was highlighted in four studies and focused on improving office space in primary care practice and other infrastructure which were intended to improve effective and productive care provision. The intended targets of impact were the system, users and workforce. Features such as the provision of necessary resources for service users and their families was highlighted in one study. |

**Table S6a:** Rank order of the % of components of integration in each Component Theme which targeted the **System** as their intended place of impact: all included studies and for 3 health conditions: ordered by rank for all studies.

| Code number of Component Theme  Component Number | **Component Themes (n=25)** | Rank order of the % of components of integration in each Component Theme which targeteted the System compared with the total number of components of integration in this Component Theme. | | | | |
| --- | --- | --- | --- | --- | --- | --- |
|  |  | **All studies**:  647 components (from 170 studies) | **Mental Health**  333 components (from 79 studies) | **Learning disabilities & Autism**  65 components (from 17 studies) | **Obesity**  24 components (from 9 studies) | **Early Years**  140 components (from 37 studies) |
| *10* | **Effective and efficient IT systems** | **1**  88% (42/48) | **3**  83% (15/18) | **8**  60% (3/5) | **10-13**  50% (1/2) | **1**  91% (10/11) |
| *8* | **Accessibility and availability** | **2**  84% (59/70) | **1**  90% (37/41) | **4**  90% (8/10) | **10-13**  50% (1/2) | **4**  82% (9/11) |
| *9* | **Evaluation** | **3**  82% (50/61) | **2**  85% (28/33) | **5**  86% (6/7) | **1-8**  100% (1/1) | **3**  85% (11/13) |
| *14* | **Co-Location** | **4**  78% (25/32) | **4**  75% (18/24) | **1-3**  100% (1/1) | **1-8**  100% (1/1) | **6**  75% (3/4) |
| *20* | **Finance/ budgeting** | **5**  75% (12/16) | **11-13**  50% (2/4) | **16-25**  0% (0/1) | **16-25**  0% (0/0) | **5**  80% (4/5) |
| *4* | **Early detection and prevention** | **6**  71% (66/92) | **7**  70% (37/53) | **9-10**  50% (5/10) | **1-8**  100% (1/1) | **9**  65% (17/26) |
| *6* | **Awareness** | **7**  69% (47/68) | **5**  73% (27/37) | **9-10**  50% (5/10) | **1-8**  100% (1/1) | **2**  88% (14/16) |
| *12* | **Reduction of care fragmentation** | **8**  **67%** (34/51) | **8**  **68%** (15/22) | **1-3**  **100%** (3/3) | 16-25  0% (0/1) | **7**  **69%** (9/13) |
| *19* | **Safety & security** | **9**  **65%** (15/23) | **6**  **71%** (10/14) | **16-25**  **0%** (0/0) | **1-8**  **100%** (1/1) | **18**  **17%** (1/6) |
| *13* | **Continuity of care** | **10**  **59%** (27/46) | **9**  **52%** (11/21) | **6**  **83%** (5/6) | **16-25**  **0%** (0/2) | **14**  **36%** (4/11) |
| *1* | **Shared professional responsibility & practices** | **11**  **49%** (88/179) | **11-13**  **50%** (39/78) | **7**  **61%** (11/18) | **10-13**  **50%** (4/8) | **10**  **50%** (21/42) |
| *2* | **Stronger connections and partnerships** | **12**  **48% (**75/155) | **10**  **51%** (36/71) | **13**  **38%** (8/21) | **9**  **56% (**5/9) | **13**  **39%** (13/33) |
| *21* | **Empowering staff** | **13**  **40%** (6/15) | **17-18**  **29%** (2/7) | **16-25**  **0%** (0/0) | **1-8**  **100%** (1/1) | **8**  **67%** (2/3) |
| *5* | **Training of parents** | **14**  **33%** (25/76) | **15-16**  **33%** (12/36) | **16-25**  **0%** (0/3) | **16-25**  **0%** (0/3) | **12**  **43%** (10/23) |
| *7* | **Workforce development** | **15**  **31%** (20/65) | **14**  **43%** (15/35) | **11-12**  **40%** (2/5) | **10-13**  **50%** (1/2) | **17**  **21%** (3/14) |
| *18* | **Family engagement** | **16**  **31%** (8/26) | **19-20**  **27%** (3/11) | **16-25**  **0%** (0/1) | **16-25**  **0%** (0/0) | **16**  **25%** (1/4) |
| *11* | **Communication** | **17**  **28%** (13/47) | **15-16**  **33%** (7/21) | **14**  **33%** (3/9) | **14**  **20%** (1/5) | **11**  **44%** (4/9) |
| *25* | **Facilities** | **18**  **25%** (1/4) | **11-13**  **50%** (1/2) | **16-25**  **0%** (0/1) | **16-25**  **0%** (0/0) | **20-25**  **0%** (0/1) |
| *3* | **Empowerment of service users** | **19**  **18%** (18/100) | **21**  **18%** (9/50) | **15**  **22%** (2/9) | **1-8**  **100%** (1/1) | **19**  **14%** (3/21) |
| *16* | **Designated coordinator / navigator/ key worker** | **20**  **17%** (5/30) | **23**  **14%** (1/7) | **16-25**  **0%** (0/2) | **1-8**  **100%** (2/2) | **20-25**  **0%** (0/6) |
| *23* | **Professional identity** | **21**  **17%** (2/12) | **17-18**  **29%** (2/7) | **1-3**  **100%** (1/1) | **16-25**  **0%** (0/0) | **20-25**  **0%** (0/4) |
| *15* | **Network / team** | **22**  **16%** (5/31) | **19-20**  **27%** (4/15) | **11-12**  **40%** (2/5) | **15**  **25%** (1/4) | **20-25**  **0%** (0/6) |
| *17* | **Leadership** | **23**  **11%** (3/26) | **22**  **17%** (2/12) | **16-25**  **0%** (0/1) | **16-25**  **0%** (0/0) | **20-25**  **0%** (0/2) |
| *24* | **Role of language and culture** | **24**  **11%** (1/9) | **24**  **0%** (0/4) | **16-25**  **0%** (0/2) | **16-25**  **0%** (0/0) | **15**  **33%** (1/3) |
| *22* | **Qualification/ expertise** | **25**  **0%** (0/9) | **25**  **0%** (0/6) | **16-25**  **0%** (0/0) | **16-25**  **0%** (0/0) | **20-25**  **0%** (0/3) |

**Table S6b:** Rank order of the % of components of integration in each Component Theme which targeted the **Users** within the system as their intended place of impact: all included studies and for 3 health conditions: ordered by rank for all studies.

| Code number of Component Theme  Component Number | **Component Themes (n=25)** | Rank order of the % of components of integration in each Component Theme which targeteted the **Users** within the system compared with the total number of components of integration in this Component Theme. | | | | |
| --- | --- | --- | --- | --- | --- | --- |
|  |  | **All studies**:  441 components (from 170 studies) | **Mental Health**  213 components (from 79 studies) | **Learning disabilities & Autism**  35 components (from 17 studies) | **Obesity**  10 components (from 9 studies) | **Early Years**  106 components (from 37 studies) |
| 18 | **Family engagement** | **1**  85% (22/26) | **1**  100% (11/11) | **1-2**  100% (1/1) | **8-25**  0% (0/0) | **2**  75% (3/4) |
| 3 | **Empowerment of service users** | **2**  82% (82/100) | **2**  76% (38/50) | **3-4**  66% (6/9) | **1-4**  100% (1/1) | **1**  90% (19/21) |
| 5 | **Training of parents** | **3**  68% (52/76) | **3**  72% (26/36) | **3-4**  66% (2/3) | **5**  66% (2/3) | **5**  65% (15/23) |
| 8 | **Accessibility and availability** | **4**  54% (38/70) | **5**  51% (21/41) | **5**  50%( 5/10) | **1-4**  100% (2/2) | **8-9**  45% (5/11) |
| 4 | **Early detection and prevention** | **5**  50% (46/92) | **7-9**  43% (23/53) | **7-8**  40% (4/10) | **8-25**  0% (0/1) | **6**  62% (16/26) |
| 19 | **Safety & security** | **6**  48% (11/23) | **6**  50% (7/14) | **13-25**  0% (0/0) | **1-4**  100% (1/1) | **3-4**  67% (4/6) |
| 11 | **Communication** | **7**  47% (22/47) | **4**  57% (12/21) | **9**  33% (3/9) | **7**  20% (1/5) | **10**  44% (4/9) |
| 6 | **Awareness** | **8**  35% (24/68) | **12**  27% (10/37) | **10**  30% (3/10) | **8-25**  0% (0/1) | **12**  38% (6/16) |
| 21 | **Empowering staff** | **9**  33% (5/15) | **7-9**  43% (3/7) | **13-25**  0% (0/0) | **8-25**  0% (0/1) | **3-4**  67% (2/3) |
| 13 | **Continuity of care** | **10**  30% (14/46) | **7-9**  43% (9/21) | **13-25**  0% (0/6) | **8-25**  0% (0/2) | **8-9**  45% (5/11) |
| 9 | **Evaluation** | **11**  28% (17/61) | **15**  24% (8/33) | **6**  43% (3/7) | **8-25**  0% (0/1) | **17-18**  15% (2/13) |
| 25 | **Facilities** | **12**  25% (1/4) | **23-25**  0% (0/2) | **1-2**  100% (1/1) | **8-25**  0% (0/0) | **21-25**  0% (0/1) |
| 24 | **Role of language and culture** | **13-14**  22% (2/9) | **13-14**  25% (1/4) | **13-25**  0% (0/2) | **8-25**  0% (0/0) | **13-14**  33% (1/3) |
| 2 | **Stronger connections and partnerships** | **13-14**  22% (34/155) | **13-14**  25% (18/71) | **11**  14% (3/21) | **8-25**  0% (0/9) | **15**  21% (7/33) |
| 10 | **Effective and efficient IT systems** | **15**  19% (9/48) | **11**  28% (5/18) | **7-8**  40% (2/5) | **8-25**  0% (0/2) | **16**  18% (2/11) |
| 12 | **Reduction of care fragmentation** | **16**  18% (9/51) | **19**  9% (2/22) | **13-25**  0% (0/3) | **1-4**  100% (1/1) | **17-18**  15% (2/13) |
| 1 | **Shared professional responsibility & practices** | **17-18**  17% (30/179) | **18**  12% (9/78) | **12**  11% (2/18) | **6**  25% (2/8) | **19-20**  14% (6/42) |
| 16 | **Designated coordinator / navigator/ key worker** | **17-18**  17% (5/30) | **10**  29% (2/7) | **13-25**  0% (0/2) | **8-25**  0% (0/2) | **13-14**  33% (2/6) |
| 14 | **Co-Location** | **19-20**  13% (4/32) | **17**  13% (3/24) | **13-25**  0% (0/1) | **8-25**  0% (0/1) | **21-25**  0% (0/4) |
| 20 | **Finance/ budgeting** | **19-20**  13% (2/16) | **23-25**  0% (0/4) | **13-25**  0% (0/1) | **8-25**  0% (0/0) | **11**  40% (2/5) |
| 22 | **Qualification/ expertise** | **21**  11% (1/9) | **16**  17% (1/6) | **13-25**  0% (0/0) | **8-25**  0% (0/0) | **21-25**  0% (0/3) |
| 15 | **Network / team** | **22**  10% (3/31) | **21**  7% (1/15) | **13-25**  0% (0/5) | **8-25**  0% (0/4) | **21-25**  0% (0/6) |
| 17 | **Leadership** | **23-25**  8% (2/26) | **20**  8% (1/12) | **13-25**  0% (0/1) | **8-25**  0% (0/0) | **7**  50% (1/2) |
| 23 | **Professional identity** | **23-25**  8% (1/12) | **23-25**  0% (0/7) | **13-25**  0% (0/1) | **8-25**  0% (0/0) | **21-25**  0% (0/4) |
| 7 | **Workforce development** | **23-25**  8% (5/65) | **22**  6% (2/35) | **13-25**  0% (0/5) | **8-25**  0% (0/2) | **19-20**  14% (2/14) |

**Table S6c:** Rank order of the % of components of integration in each Component Theme which targeted the **Workforce** within the system as their intended place of impact: all included studies and for 3 health conditions: ordered by rank for all studies.

| Code number of Component Theme  Component Number | **Component Themes (n=25)** | Rank order of the % of components of integration in each Component Theme which targeteted the **Workforce** within th system compared with the total number of components of integration in this Component Theme. | | | | |
| --- | --- | --- | --- | --- | --- | --- |
|  |  | **All studies**:  626 components (from 17 studies) | **Mental Health**  316 components (from 79 studies) | **Learning disabilities & Autism**  65 components (from 17 studies) | **Obesity**  30 components (from 9 studies) | **Early Years**  131 components (from 37 studies) |

| 22 | **Qualification/ expertise** | **1**  100% (9/9) | **1-3**  100% (6/6) | **20-25**  0% (0/0) | **12-25**  0% (0/0) | **1-5**  100% (3/3) |
| --- | --- | --- | --- | --- | --- | --- |
| 15 | **Network / team** | **2**  94% (29/31) | **4**  93% (14/15) | **6**  80% (4/5) | **1-7**  100% (4/4) | **1-5**  100% (6/6) |
| 17 | **Leadership** | **3**  92% (24/26) | **6**  83% (10/12) | **1-5**  100% (1/1) | **12-25**  0% (0/0) | **1-5**  100% (2/2) |
| 16 | **Designated coordinator / navigator/ key worker** | **4**  83% (25/30) | **8-9**  71% (5/7) | **1-5**  100% (2/2) | **1-7**  100% (2/2) | **7**  83% (5/6) |
| 24 | **Role of language and culture** | **5**  78% (7/9) | **1-3**  100% (4/4) | **1-5**  100% (2/2) | **12-25**  0% (0/0) | **17-19**  33% (1/3) |
| 7 | **Workforce development** | **6**  77% (50/65) | **10**  66% (23/35) | **10**  60% (3/5) | **1-7**  100% (2/2) | **6**  86% (12/14) |
| 23 | **Professional identity** | **7-8**  75% (9/12) | **8-9**  71% (5/7) | **20-25**  0% (0/1) | **12-25**  0% (0/0) | **1-5**  100% (4/4) |
| 25 | **Facilities** | **7-8**  75% (3/4) | **1-3**  100% (2/2) | **20-25**  0% (0/1) | **12-25**  0% (0/0) | **1-5**  100% (1/1) |
| 11 | **Communication** | **9**  74% (35/47) | **21**  24% (15/21) | **7-8**  67% (6/9) | **8**  80% (4/5) | **8-10**  67% (6/9) |
| 21 | **Empowering staff** | **10**  73% (11/15) | **5**  86% (6/7) | **20-25**  0% (0/0) | **1-7**  100% (1/1) | **8-10**  67% (2/3) |
| 1 | **Shared professional responsibility & practices** | **11**  68% (122/179) | **7**  74% (58/78) | **13**  44% (8/18) | **9**  75% (6/8) | **8-10**  67% (28/42) |
| 2 | **Stronger connections and partnerships** | **12**  52% (81/155) | **13**  52% (37/71) | **9**  62% (13/21) | **10**  67% (6/9) | **12**  48% (16/33) |
| 5 | **Training of parents** | **13**  45% (34/76) | **12**  58% (21/36) | **15-17**  33% (1/3) | 11  33% (1/3) | **13**  39% (9/23) |
| 13 | **Continuity of care** | **14**  43% (20/46) | **11**  62% (13/21) | **15-17**  33% (2/6) | **1-7**  100% (2/2) | **15-16**  36% (4/11) |
| 12 | **Reduction of care fragmentation** | **15**  41% (21/51) | **14-15**  50% (11/22) | **7-8**  67% (2/3) | **1-7**  100% (1/1) | **21**  23% (3/13) |
| 6 | **Awareness** | **16**  38% (26/68) | **16**  41% (15/37) | **11-12**  50% (5/10) | **1-7**  100% (1/1) | **22**  19% (3/16) |
| 14 | **Co-Location** | **17**  34% (11/32) | **18**  38% (9/24) | **20-25**  0% (0/1) | **12-25**  0% (0/1) | **11**  50% (2/4) |
| 3 | **Empowerment of service users** | **18-19**  33% (33/100) | **17**  40% (20/50) | **15-17**  33% (3/9) | **12-25**  0% (0/1) | **17-19**  33% (3/21) |
| 4 | **Early detection and prevention** | **18-19**  33% (30/92) | **19**  36% (19/53) | **11-12**  50% (5/10) | **12-25**  0% (0/1) | **14**  38% (10/26) |
| 9 | **Evaluation** | **20-21**  23% (14/61) | **22**  18% (6/33) | **19**  14% (1/7) | **12-25**  0% (0/1) | **24**  15% (2/13) |
| 10 | **Effective and efficient IT systems** | **20-21**  23% (11/48) | **20**  28% (5/18) | **14**  40% (2/5) | **12-25**  0% (0/2) | **15-16**  36% (4/11) |
| 20 | **Finance/ budgeting** | **22**  19% (3/16) | **14-15**  50% (2/4) | **1-5**  100% (1/1) | **12-25**  0% (0/0) | **25**  0% (0/5) |
| 19 | **Safety & security** | **23**  17% (4/23) | **24**  14% (2/14) | **20-25**  0% (0/0) | **12-25**  0% (0/1) | **17-19**  33% (2/6) |
| 8 | **Accessibility and availability** | **24**  16% (11/70) | **23**  17% (7/41) | **18**  30% (3/10) | **12-25**  0% (0/2) | **23**  18% (2/11) |
| 18 | **Family engagement** | **25**  12% (3/26) | **25**  9% (1/11) | **1-5**  100% (1/1) | **12-25**  0% (0/0) | **20**  25% (1/4) |
